# Migration Patterns of Subgenus *Alnus* in Europe since the Last Glacial Maximum: A Systematic Review

**DOI:** 10.1371/journal.pone.0088709

**Published:** 2014-02-21

**Authors:** Jan Douda, Jana Doudová, Alena Drašnarová, Petr Kuneš, Věroslava Hadincová, Karol Krak, Petr Zákravský, Bohumil Mandák

**Affiliations:** 1 Department of Ecology, Faculty of Environmental Sciences, Czech University of Life Sciences Prague, Prague, Czech Republic; 2 Institute of Botany, Academy of Sciences of the Czech Republic, Průhonice, Czech Republic; 3 Department of Botany, Faculty of Science, Charles University in Prague, Prague, Czech Republic; Centre National de la Recherche Scientifique, France

## Abstract

**Background/Aims:**

Recently, new palaeoecological records supported by molecular analyses and palaeodistributional modelling have provided more comprehensive insights into plant behaviour during the last Quaternary cycle. We reviewed the migration history of species of subgenus *Alnus* during the last 50,000 years in Europe with a focus on (1) a general revision of *Alnus* history since the Last Glacial Maximum (LGM), (2) evidence of northern refugia of *Alnus* populations during the LGM and (3) the specific history of *Alnus* in particular European regions.

**Methodology:**

We determined changes in *Alnus* distribution on the basis of 811 and 68 radiocarbon-dated pollen and macrofossil sites, respectively. We compiled data from the European Pollen Database, the Czech Quaternary Palynological Database, the Eurasian Macrofossil Database and additional literature. Pollen percentage thresholds indicating expansions or retreats were used to describe patterns of past *Alnus* occurrence.

**Principal Findings:**

An expansion of *Alnus* during the Late Glacial and early Holocene periods supports the presence of alders during the LGM in southern peninsulas and northerly areas in western Europe, the foothills of the Alps, the Carpathians and northeastern Europe. After glaciers withdrew, the ice-free area of Europe was likely colonized from several regional refugia; the deglaciated area of Scandinavia was likely colonized from a single refugium in northeastern Europe. In the more northerly parts of Europe, we found a scale-dependent pattern of *Alnus* expansion characterised by a synchronous increase of *Alnus* within individual regions, though with regional differences in the times of the expansion. In southern peninsulas, the Alps and the Carpathians, by contrast, it seems that *Alnus* expanded differently at individual sites rather than synchronously in whole regions.

**Conclusions:**

Our synthesis supports the idea that northern LGM populations were important sources of postglacial *Alnus* expansion. The delayed *Alnus* expansion apparent in some regions was likely a result of environmental limitations.

## Introduction

The recent distribution of species in the Northern Hemisphere has been significantly influenced by processes occurring in the last Quaternary cycle, during the last glacial period and subsequent Holocene warming [1], [2]. The ‘classic’ paradigm states that during the Last Glacial Maximum (LGM, i.e., from 26.5 to 19 to 20 thousand years before present (kyr BP) [3]), temperate plant species, particularly climate-sensitive trees, were harboured in low-latitude refugia. In Europe, southern peninsulas (i.e., Iberian, Italian and Balkan) served as refugial areas for many species [4], [5].

Recently, new palaeoecological records supported by molecular analyses and palaeodistributional modelling have provided more comprehensive insights into plant behaviour during the last Quaternary cycle [6], [7]. In eastern Europe, more northerly distributions of many temperate and boreal plants during the last glacial period have been confirmed, although fossil records directly from the LGM are scarce. The eastern Alps, northern Dinaric Alps, the Carpathians and the Pannonian region probably served as northern refugia for many temperate tree species, namely *Abies alba*, *Carpinus betulus*, *Fagus sylvatica*, *Taxus baccata* and *Ulmus* [6], [8], [9], [10]. Open taiga and hemiboreal forests dominated by *Larix*, *Pinus*, *Picea* and *Betula* likely occurred in the northern Carpathians, Belarus and the northwestern Russian plains [11], [12], [13].

The early postglacial expansion of trees in northern areas thus need not reflect migration from southern regions but may be the result of the population growth and expansion of small tree populations persisting in scattered refugia relatively close to the margin of the ice sheet [14], [15], [16]. Climatic warming has been determined as the most important driver initiating the expansion of trees [1]. However, regional differences in climatic and environmental conditions recorded for the Late Glacial and early Holocene periods could have resulted in nontrivial species-specific and regionally dependent patterns of expansion [17], [18], [19].

### Taxonomic Status

The genus *Alnus* Mill. belongs to the family Betulaceae [20], [21]. The oldest macrofossil records assigned to *Alnus* have been reported from the middle Eocene, but fossil pollen grains of *Alnus* from the Late Cretaceous have also been found [22]. The genus *Alnus* comprises about 29 to 35 species of monoecious trees and shrubs distributed throughout the Northern Hemisphere and along the Andes in South America [23]. In Europe, three species of subgenus *Alnus* (i.e. *Alnus cordata*, *A*. *glutinosa* and *A*. *incana*) and one species of subgenus *Alnobetula* (*A*. *alnobetula* (Ehrh.) K. Koch) occur [24]. It has been estimated using molecular methods that the subgenera *Alnus* and *Alnobetula* diverged in the Eocene, 48.6 million years (Myr) BP [25]. *A. cordata* separated from the *A. glutinosa-incana* complex in the Oligocene (22.9 Myr BP), and *A. glutinosa* and *A*. *incana* diverged in the Pliocene (7.9 Myr BP) [25].

### Upper Pleistocene and Holocene *Alnus* History

Pleistocene pollen and macrofossil data indicate repeated population increases and decreases of *Alnus* in Europe, reflecting climate oscillations between glacial periods and interglacials, particularly noticeable in the Middle and Upper Pleistocene [5], [26], [27]. The majority of Upper Pleistocene pollen profiles support a common presence of *Alnus* in the Eemian interglacial (Marine Isotope Stage, MIS 5e–5d) throughout Europe (i.e., 125–115 kyr BP) and its disappearance after the start of the last glacial period [i.e., Hollerup (DK) – [28]; Tenaghi Philipon (GR) – [26]; Valle di Castiglione (IT) – [29]; Les Echets (FR), La Grande Pile (FR) – [30], [31]; Praclaux Crater (FR), Ribains (FR) – [32]; Ioannina (GR) – [5]; Jammertal (DE) – [33]].

In their classic study, Huntley and Birks [1] assumed that the main source refugia for the *Alnus* expansion after the LGM lay in the eastern Alps, the Carpathians and the Ukrainian lowlands. Other LGM refugia were located in Corsica, western France, northern Spain and northwestern Russia. The authors supposed that the Holocene migration of *Alnus* likely began somewhere in eastern Europe and continued by the northward expansion of *A*. *glutinosa* (L.) Gaertn. and *A*. *incana* (L.) Moench to the Baltic and Scandinavia and by the westward expansion of *A. glutinosa* along the southern shore of the North Sea as far as the British Isles [1].

A large-scale genetic survey that included Europe and Turkey and focused on the postglacial history of *Alnus glutinosa* accepted Huntley and Birks’ migration patterns [34]. King and Ferris [34] revealed 13 cpDNA haplotypes of *A*. *glutinosa* mainly associated with southern European peninsulas. They suggested that two of these haplotypes colonized northern and temperate Europe from LGM refugia located in the Carpathians. While the first haplotype expanded primarily into western Europe, the second mainly colonized northern Europe [34]. However, the presence of only two haplotypes in the northern part of Europe limits a more detailed determination of *A. glutinosa* migration patterns. Surprisingly, no follow-up study focusing on postglacial migration pattern of *A*. *glutinosa* has since been published. No phylogeographic study has been performed for *A. incana* thus far, either.

Since the seminal studies of Huntley and Birks [1] and King and Ferris [34] were published, many palaeoecological studies have presented new knowledge about the history of *Alnus* in Europe during the last glacial period and Holocene. We reviewed the migration history of species of subgenus *Alnus*, including *A. glutinosa* and *A. incana*, during the last 50,000 years in Europe based on large numbers of pollen records and macrofossil remains. Pollen of different species of subgenus *Alnus* (further collectively referred to as “*Alnus*”) is indistinguishable in palaeoecological studies, but alder species can be identified based on macrofossil remains. In particular, we focused on (1) a general revision of *Alnus* history since the LGM, (2) evidence of northern refugia of *Alnus* during the LGM and (3) the specific history of *Alnus* in particular European regions.

*Alnus* species are keystones of alluvial and wetland habitats [35], [36] distributed through the European forest zones from the northern treeline to the Mediterranean. Understanding their last glacial occurrence and postglacial migration pattern may shed light upon the resistance and resilience of wetland forest habitats in the course of global climate change. The results of our study allow us to propose guidelines for the sampling design and interpretation of a future detailed phylogeographic and population-genetic survey of *Alnus* species in Europe.

## Materials and Methods

### Study Species

**Figure 1:**
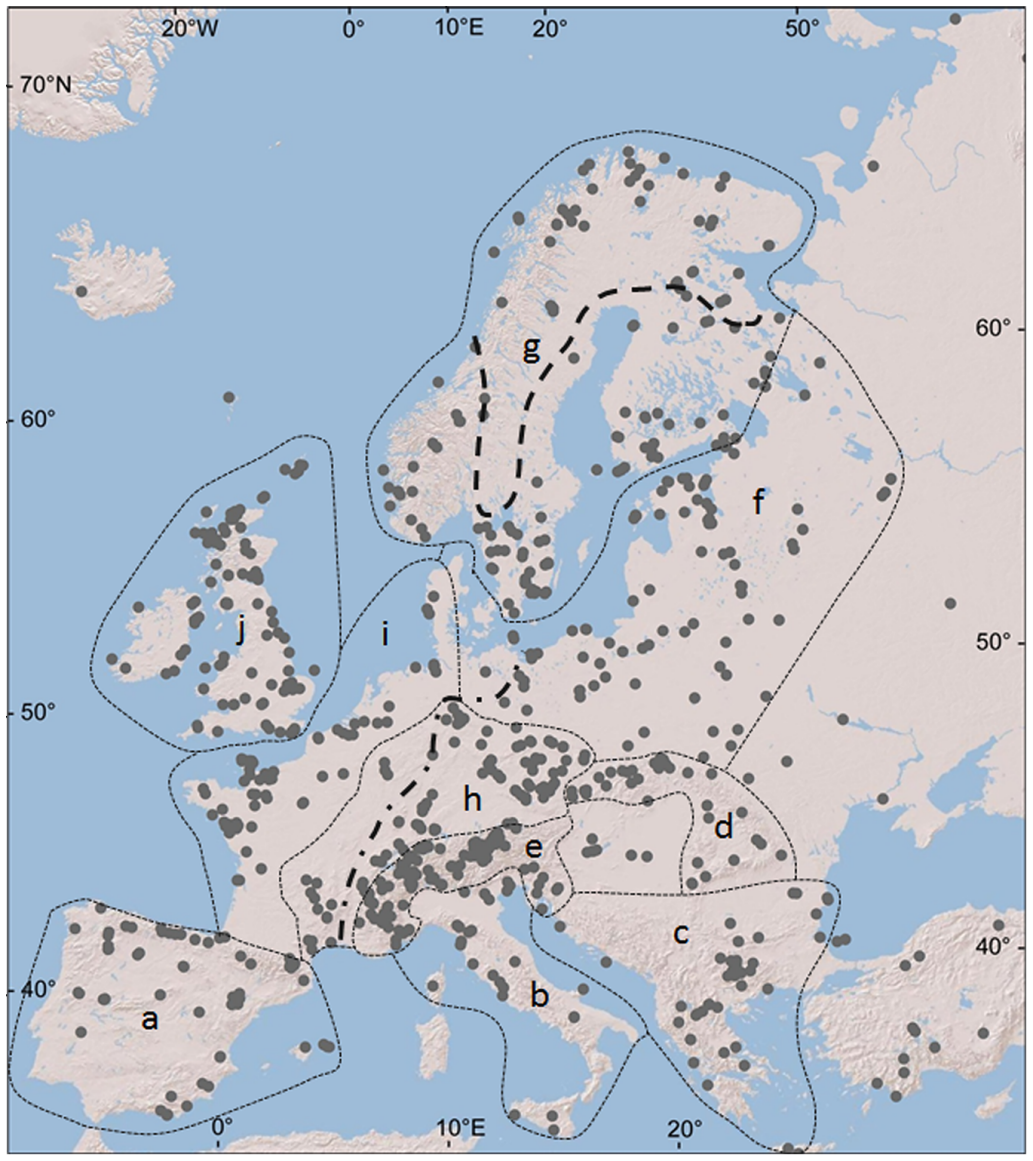
Pollen sites (dots) and European regions (dotted lines) included in this study. a, Iberian region; b, Italian region; c, Balkan region; d, the Carpathians; e, the Alps; f, Baltic and northeastern European plains; g, Scandinavia; h, Hercynian Mountains and Massif Central; i, western European plain; j, British Isles. Bold dashed and dashed-dotted lines show the northern boundary of *Alnus glutinosa* in Scandinavia and the western range boundary of *A. incana* in Western Europe, respectively [24].

Two common tree species of *Alnus* grow natively in Europe [24]. Black alder (*A. glutinosa*) is considered a temperate tree. It commonly occurs in the lowlands and mountains across Europe except Scandinavia, where it is associated with a coastal oceanic climate in southern areas [37] (Figure 1). The cold-climate limitation also likely affects its distribution in high-elevation mountainous areas, where black alder populations are often absent. Scarce distributions are found in the Mediterranean region and in the arid Great Hungarian plains, the Ukraine and the Russian steppe zone. Outside Europe, the distribution extends as far as western Siberia and the mountains of Turkey, Iran and North Africa [24], [38]. In Corsica and southern Italy, *A. glutinosa* grows sympatrically with *A. cordata* (Loisel.) Duby.

Grey alder (*Alnus incana*) is considered a boreal and mountain tree. Similar to Norway spruce (*Picea abies*), the range of *A. incana* is divided into a northern and a southern area, which meet in the Polish lowlands. In northern Europe, *A. incana* continuously covers the east Baltic region and all of Scandinavia with a northern margin at latitudes greater than 70°N [24], [37]. In northern Scandinavia, the nominal subspecies grows sympatrically with *A. incana* subsp. *kolaensis* (Orlova) Á. Löve & D. Löve [24]. The distribution of grey alder continues eastwards across European Russia to western Siberia, which contrasts with its patchy mountain occurrence in the southern part of the range linked to the Alps, the northern Apennines, the Hercynian Mountains, the Carpathians, the Bulgarian Mountains, the Dinaric Alps, the Caucasus and Turkey [24].

*Alnus glutinosa* and *A. incana* dominate in floodplain and swamp forests. These species are indifferent to soil nutrient conditions, except for extremely poor peat bogs. Seeds are dispersed effectively by water, while wind dispersal is commonly limited to the vicinity of the parent tree [38]. Under unfavourable environmental conditions, such as in cold climates, *A. incana* is able to survive and reproduce by clonal growth [39]. Compared with the relatively short-lived *A. incana* (c. 20–50 years), *A. glutinosa* is a long-lived tree (c. 100–120 years), although the age of reproduction is similar for the two species (i.e., 10–20 years) [37], [38].

### Pollen Data

This systematic review follows the PRISMA (Preferred Reporting Items for Systematic Reviews and Meta-Analyses) statement as a guide [40] (see Checklist S1). We compiled freely available data from the European Pollen Database (EPD, http://europeanpollendatabase.net, [41]), the Czech Quaternary Palynological Database (PALYCZ, http://botany.natur.cuni.cz/palycz, [42]) and additional literature (Figure 1). The search for additional literature was performed in September 2011 in Web of Science and augmented by Google Scholar. The search included combinations and derivations of the following terms: radiocarbon dates, pollen, wood remains, macrofossils, glacial, vegetation, LGM, Holocene and Europe. To guarantee the chronological accuracy of changes in *Alnus* distribution, we used only pollen data with radiocarbon dating. In total, we used 553 and 258 pollen profiles from databases and the literature, respectively. The list of original publications and sites available in September 2011 is provided in Tables S1 and S3.

Age-depth models were constructed and radiocarbon dates were calibrated (cal.) for all profiles in the EPD [43], PALYCZ and publications using the CLAM code [44] in R [45]. The age-depth models were constructed using smoothing-spline fitting with a default smoothing factor of 0.3 or linear interpolation with preferences for a smoothing spline. Possible errors in the pollen diagram chronology were minimised in several ways. We excluded parts of the chronology outside the marginal ^14^C dates. Additionally, to determine the oldest unquestionable time of expansion, we checked parts of the pollen diagrams indicating the start of the *Alnus* expansion (i.e., ≥2.5% pollen threshold) to determine whether i) the nearest radiocarbon date is closer than 2,000 years to time of the expansion, ii) there is no presence of reworked pollen or iii) the expansion does not start at the end of the previous 1,000-year interval. Reworking was assumed when an isolated pollen spectrum with *Alnus* ≥2.5% was recorded or when the basal spectra of *Alnus* ≥2.5% were followed by a steep decrease in pollen.

To describe the temporal patterns of *Alnus* occurrence at particular sites, we recorded the pollen percentage of *Alnus* at 1,000-year intervals in the time period from the present to 26 cal. kyr BP (i.e., the start of the LGM) and at 5,000-year intervals in the time period preceding the LGM. The average percentage of pollen of *Alnus* at the site in each time interval (1,000 and 5,000 year) was calculated by dividing the *Alnus* pollen count by the total pollen sum in each sample after excluding aquatic species, cryptogam spores and indeterminable pollen. We excluded pollen of subgenus *Alnobetula* from the total *Alnus* pollen count. Due to their specific pollen morphology, pollen grains of species of subgenus *Alnobetula* are identified and counted separately in palaeoecological studies [46], [47]. We chose 5,000-year intervals before 26 kyr BP because the pollen records were fragmentary and the rare radiocarbon dates do not sufficiently cover the pollen profiles. The total pollen sum was calculated in most literature sources in the same way, allowing us to determine past *Alnus* pollen value for each time period by simple visual inspection of pollen diagrams.

Because alders are high pollen producers, they are generally overrepresented in pollen diagrams [1]. Moreover, *Alnus glutinosa* and *A. incana* often dominate in swamps, at lake and stream shores and at the margins of peat bogs in close vicinity to sample sites [48]. To record the regional presence of *Alnus* from pollen diagrams, several thresholds ranging from 0.5 to 8% have been used in the literature [49], with greater agreement for 2–3% [1], [50], [51], [52]. We used the 2.5% threshold suggested for *Alnus* in a recent study comparing modern pollen data with European tree species distribution [49]. This 2.5% threshold corresponds to the presence of *Alnus* within approximately 50 km of a pollen site [49]. We also incorporated the threshold of 0.5% as an indicator of possible scarce regional occurrence despite the risk of contamination by long-distance pollen transport. Lisitsyna et al. [49] still found strong agreement between pollen presence defined by the 0.5% threshold and the regional occurrence of *Alnus*. Pollen values greater than the 10% threshold are assumed to correspond to the occurrence of an *Alnus*-dominated forest at the site [1], [37], [51]. In summary, four percentage categories were used to describe the patterns of past *Alnus* occurrence, where less than 0.5% indicates the regional absence of a species, 0.5–2.5% may be the result of long-distance pollen transport but could also capture the presence of relatively small populations in the region, 2.5–10% indicates a species’ presence within the region, and values greater than 10% indicate the local presence of a species at the site. The description of *Alnus* distribution in the results and discussion section is based on ≥2.5% pollen records to eliminate possible misinterpretation based on the 0.5% threshold.

### Macrofossil Data

To obtain macrofossil evidence (e.g., cones, fruits, male catkins, twigs, wood pieces), we used free data available from the Eurasian Macrofossil Database (NEMD, http://oxlel.zoo.ox.ac.uk/reference-collection, [13]) and additional published records. In total, we used macrofossil data from 14 sites in the database and 54 sites in the literature (Tables S2 and S3). Macrofossils of *Alnus glutinosa* and *A. incana* were determined at 38 and 15 sites, respectively. Macrofossil records were assigned according to 1,000- or 5,000-year pollen intervals based on constructed age-depth models (see Pollen data chapter). We interpreted only the *Alnus* presence, as it is problematic to evaluate data regarding the absence or abundance of macrofossils [9].

### Pollen and Macrofossil Maps

The pollen and macrofossil maps indicate *Alnus* occurrence at particular time periods during the last 50,000 years. We merged 5,000- and 1,000-yr intervals with a limited number of records to logical periods of the last glacial period; 50–26 cal. kyr BP includes the period preceding the LGM, 26–20 cal. kyr BP the period of the LGM and 20–15 cal. kyr BP the period after the LGM, also known as the Oldest Dryas. The macrofossil remains and maximum pollen thresholds recorded during the merged periods were plotted in maps. We also marked changes in the pollen percentages between the time periods, indicating the expansion, stability or decrease of *Alnus*. The term “*Alnus*” indicates macrofossils that were not assigned to individual species in original studies whereas the names “*Alnus glutinosa*” and “*A. incana”* refer to those that were.

### Regional Differences in Late Glacial and Holocene History

To determine the specific postglacial history of *Alnus* in individual European regions, we delimited 10 regions based on different environmental conditions in the last glacial period and the Holocene (Figure 1). The Iberian, Italian and Balkan regions include areas considered southern LGM refugia of trees (Figure 1, regions a–c). The Baltic and northeastern European plains, Scandinavia and the British Isles are regions that were largely covered by the Scandinavian ice sheet during the LGM (Figure 1, regions f, g, j). The Carpathians and Alps covered areas of potential LGM refugia for some temperate and many boreal trees (Figure 1, regions d, e). The Hercynian Mountains, the Massif Central and highlands located to the north of the Alps were mostly ice-free regions (Figure 1, region h). Ice-free lowland areas of the Western European plain were influenced by the oceanic climate (Figure 1, region i). We determined the proportion of pollen sites in each region and time period that reached the 0.5%, 2.5% and 10% thresholds. Only time intervals with more than 10 sites available in particular regions were considered in the analysis. The region of the Great Hungarian plains was excluded from all analyses because fewer than 10 pollen sites had been found there.

## Results

### Pre-LGM *Alnus* Distribution (50–26 cal. kyr BP)

**Figure 2:**
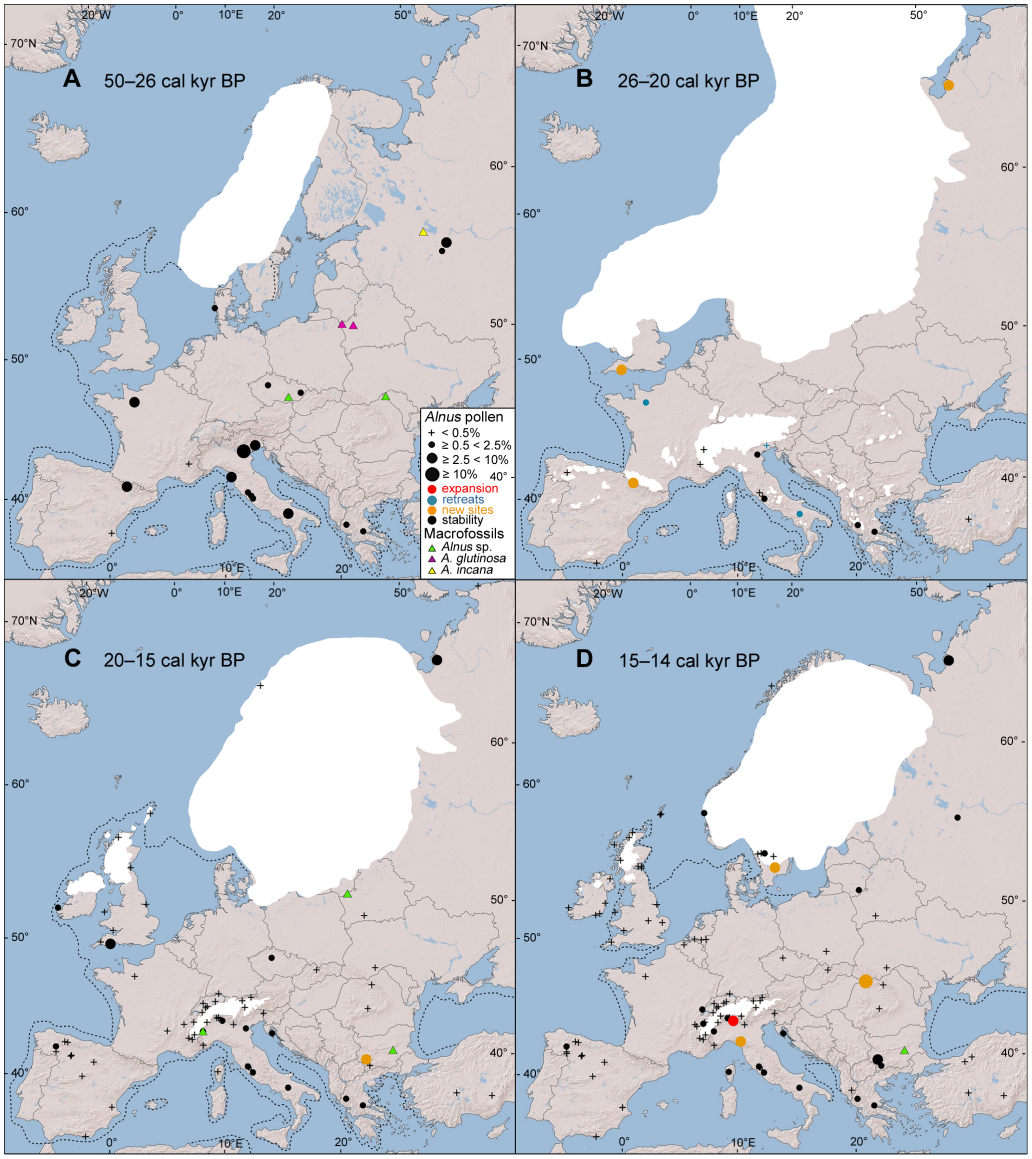
Last glacial period distribution (50–14 cal. kyr BP) of *Alnus* pollen sites. According to four classes of the percentage of *Alnus* pollen and macrofossil remains. The dot colour indicates changes compared with the previous period: red, expansion, *Alnus* pollen <2.5% in preceding period; blue, retreat, *Alnus* pollen ≥2.5% in preceding period; orange, new pollen sites of *Alnus* pollen ≥2.5%; black, stability; the course of deglaciation (white) and changes in coastline (dotted lines).

In southern Europe, *Alnus* exceeds the 2.5% pollen threshold in the Pyrenees Mountains [53] and at several Italian sites [54], [55], [56], [57] (Figure 2A). Other pollen records exceeding 2.5% have been obtained from northwestern France [58] and the western Russian plains [59]. In western Russia and Belarus, the occurrence of *A. glutinosa* and *A. incana* is supported by macrofossil remains [60], [61], [62]. *Alnus* macrofossil records are present along the northern border of the Pannonian lowlands in the Czech Republic and the northeastern foothills of the Carpathians in Romania [10] (Figure 2A).

### LGM *Alnus* Distribution (26–20 cal. kyr BP; Figure 2B)

At the LGM, the *Alnus* pollen values decrease in Italy and France (Figure 2B). The only 2.5%-threshold pollen evidence for *Alnus* occurrence in southern-European peninsulas was detected in the Pyrenees Mountains [53]. Further north in Europe, *Alnus* pollen values exceed the 2.5% threshold at two sites in the Bodmin moor in Cornwall [63] and in the Timan Ridge in Arctic Russia [64] (Figure 2B).

### Late Glacial *Alnus* Distribution (20–12 cal. kyr BP; Figure 2C, 2D, 3A and 3B)

Between 20 and 15 cal. kyr BP, the 2.5%-threshold pollen evidence of *Alnus* continues in southern England and Arctic Russia (Figure 2C). In southern Europe, only one new 2.5%-threshold pollen record has emerged in the Rila Mountains in Bulgaria [65]. Macrofossil remains of *Alnus* occur in the southwestern foothills of the Alps [66], the Thracian plain in Bulgaria [67] and southern Lithuania [61] (Figure 2C).

**Figure 3:**
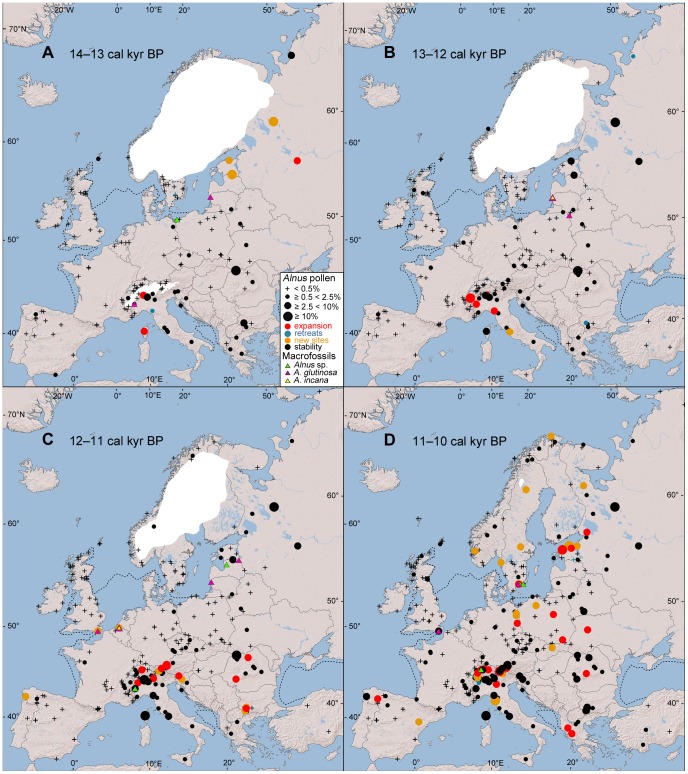
Late Glacial and early Holocene distribution (14–10 cal. kyr BP) of *Alnus* pollen sites. According to four classes of the percentage of *Alnus* pollen and macrofossil remains; for details, see Figure 2.

Between 15 and 12 cal. kyr BP, several pollen sites exceed the 2.5% *Alnus* threshold in the southwestern and western parts of the Alps (Figs 2D, 3A and 3B). Moreover, macrofossil remains of *A. glutinosa* occur there [66] (Figure 3B). South of the Alps, *Alnus* pollen increases and reaches more than 2.5% in Corsica [68] (Figure 3A), the northern Apennines [69] (Figure 3B) and central Italy [70] (Figure 3B). In the Carpathians, *Alnus* pollen records exceeding 2.5% are present in the Gutaiului Mountains in northwestern Romania [71] (Figure 2D). Sites with evidence of more than 2.5% of *Alnus* pollen emerge in southern Scandinavia [72] (Figure 2D), Estonia [73], [74] (Figure 3A) and northwestern and western Russia [75], [76] (Figure 3A). Macrofossil remains occur in Poland [77] (Figure 3A), Lithuania [61], [78] (Figure 3A and 3B) and Belarus [62] (Figure 3B).

### Holocene *Alnus* Distribution (12–0 cal. kyr BP; Figure 3C, 3D and 4, Figure S1 and S2)

At the beginning of the Holocene (i.e., 12–11 cal. kyr BP, Figure 3C), a continual increase in the number of sites with at least 2.5% *Alnus* pollen is apparent across the Alps, with the exception of the western areas. In western Europe, macrofossils of *A. glutinosa* and *A. incana* are present at the Kreekrak site in southwestern Netherlands [79] and *A. glutinosa* in Pannel Bridge, East Sussex [80]. Several pollen sites exceed the 2.5% pollen threshold in the Romanian Carpathians [81] and the Rila and Pirin Mountains in Bulgaria [82], [83], [84]. The first piece of evidence since the LGM of more than 2.5% of *Alnus* pollen has been recorded in the Iberian peninsula [85] (Figure 3C).

Between 11 and 10 cal. kyr BP (Figure 3D), many sites reach at least 2.5% of *Alnus* pollen in a large area of the Polish lowland, the northern Carpathians and Scandinavia, including its northern part [86], [87]. An increase of sites exceeding the 2.5% pollen threshold is also evident in the Iberian and the Balkan peninsula (Figure 3D).

**Figure 4:**
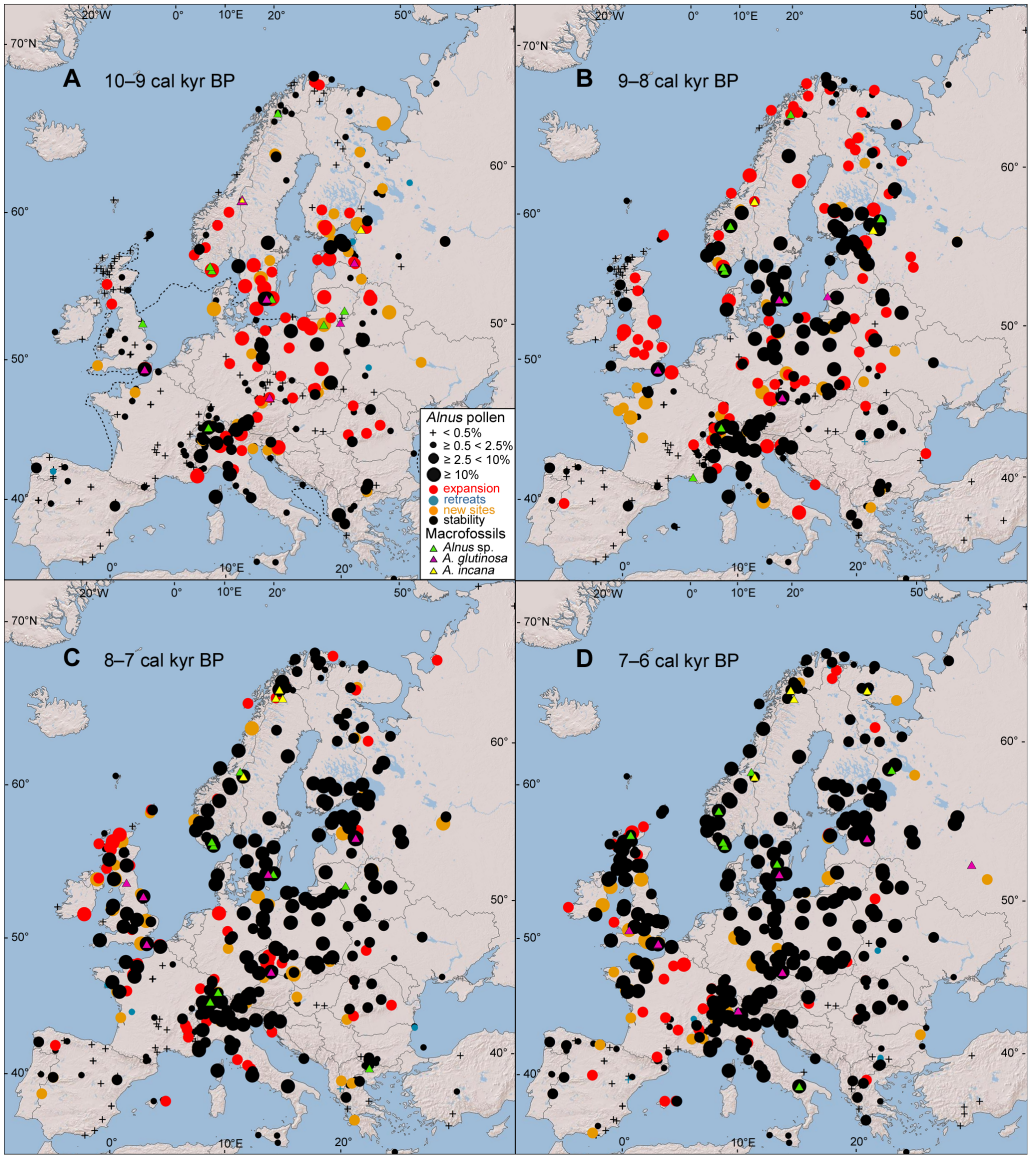
Holocene distribution (10–6 cal. kyr BP) of *Alnus* pollen sites. According to four classes of the percentage of *Alnus* pollen and macrofossil remains; for details, see Figure 2.

Between 10 and 9 cal. kyr BP, the majority of localities in the Carpathians and the Baltic region, including southern Scandinavia, exceed the 2.5% *Alnus* pollen threshold (Figure 4A). Macrofossil remains of *A. glutinosa* occur in the northern border of its recent distribution in central Sweden [14]. By contrast, few sites with more than 2.5% *Alnus* pollen are present in a large zone running from the Bohemian Massif and the northern foothills of the Alps through the Massif Central and the French Alps to western Europe and the British Isles (Figure 4A).

Between 9 and 8 cal. kyr BP, the increase of sites with more than 2.5% of *Alnus* pollen is apparent over the British Isles, the northern foothills of the Alps, the Bohemian Massif, northern Scandinavia and likely in the western European plain (Figure 4B). During the next two millennia (i.e., 8–6 cal. kyr BP), many sites with 2.5% *Alnus* evidence emerge in the French Alps, northern Scotland, Ireland and all southern peninsulas (Figure 4C and 4D). Finally, the number of sites exceeding 2.5% of *Alnus* pollen increases in the Massif Central and the remaining unoccupied areas of France between 7 and 6 cal. kyr BP (Figure 4D).

**Figure 5:**
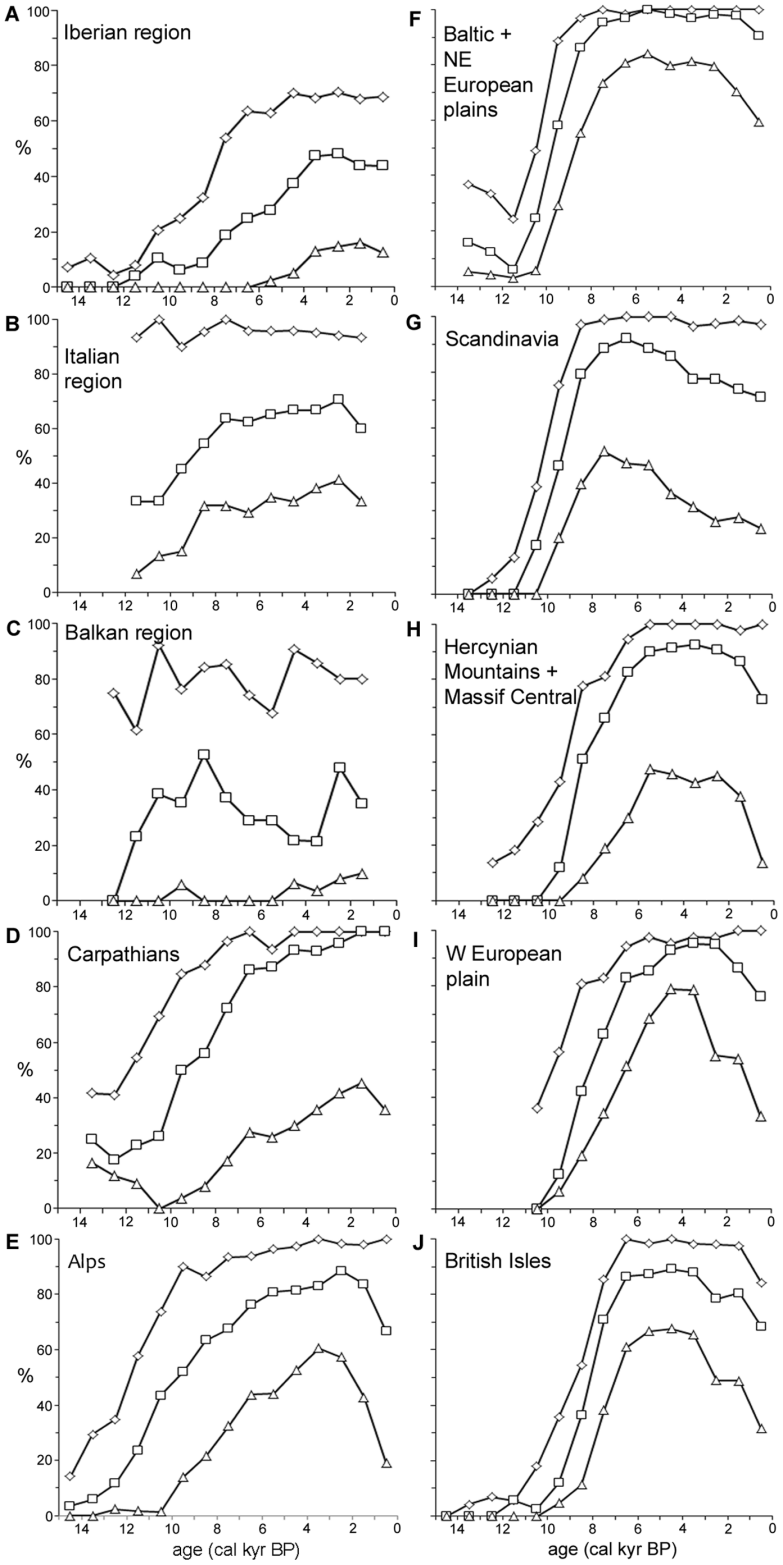
Regional proportions of *Alnus* pollen sites during the Late Glacial and Holocene periods. Pollen thresholds: 0.5% (diamonds), 2.5% (squares) and 10% (triangles). Only time intervals with more than 10 sites available in particular regions were considered.

During the period between 6 and 0 cal. kyr BP, a decrease in the number of sites with more than 2.5% *Alnus* pollen is present in large areas of Europe, likely except in the southern peninsulas and the Carpathians (Figure 5; Figure S1 and S2). After 6 cal. kyr BP, *Alnus* enters a period of retreat in northern Scandinavia and continues southward up to the present (Figure 5G; Figures S1 and S2). In other regions, a decrease is apparent during approximately the last three millennia. A relatively strong decrease appears in the Alps (Figure 5E), Hercynian Mountains (Figure 5H), the western European plain (Figure 5I) and the British Isles (Figure 5J) whereas a weak decrease is apparent in the Baltic region (Figure 5F).

### Regional Differences at the Beginning of the *Alnus* Expansion

In the southern peninsulas, the Alps and the Carpathians, there is an increase in the number of sites exceeding the 2.5% pollen threshold beginning in the Late Glacial period and increasing gradually during most of the Holocene (Figure 5A–5E). In more northerly regions, the number of sites with more than 2.5% pollen evidence rises abruptly after the beginning of the Holocene. Specifically, an increase in the number of sites in the Baltic region (Figure 5F) and Scandinavia (Figure 5G) starts between 11 and 10 cal. kyr BP and over three thousand years reaches more than 80% of occupied sites. In Hercynian Mountains (Figure 5H), the western European plain (Figure 5I) and the British Isles (Figure 5J), the expansion starts between 10 and 9 cal. kyr BP, and 80% of sites are occupied after four thousand years.

## Discussion

### Northern LGM Refugia

**Figure 6:**
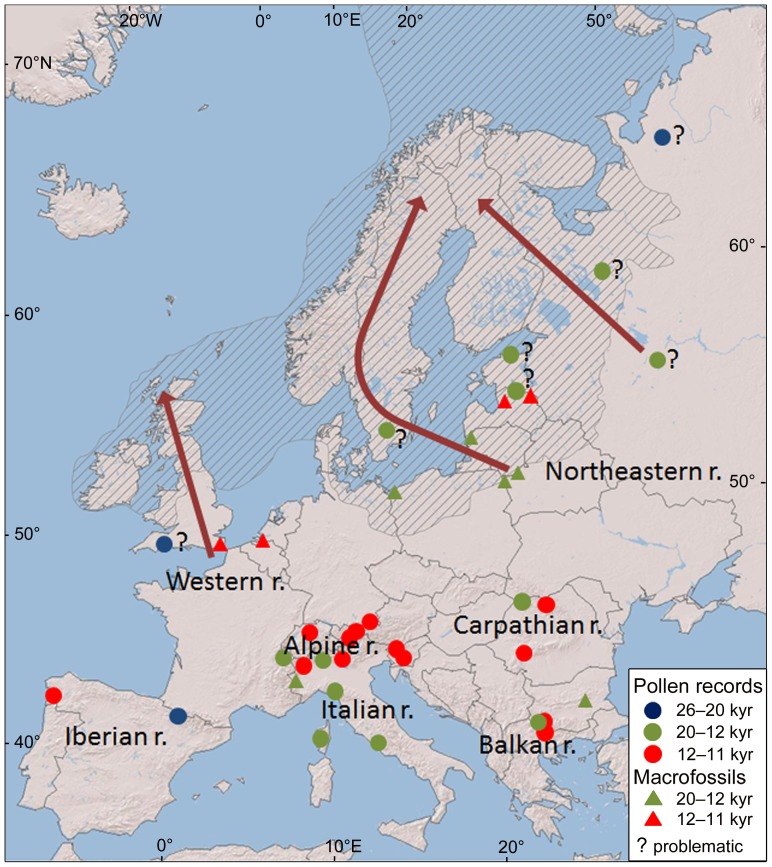
Putative Last Glacial Maximum refugia and directions of postglacial *Alnus* migration. The triangles and dots indicate macrofossil and pollen (≥2.5%) records from the LGM (blue), Late Glacial (green) and early Holocene (red). Arrows indicate directions of *Alnus* migration after northern deglaciation; question marks show problematic pollen records – possible reworking or long-distance pollen dispersal; hatching indicates the maximal extent of the ice sheet during the LGM.

For the Last Glacial Maximum, there are two records with more than 2.5% *Alnus* pollen (Figure 6) from the periglacial landscape of the Scandinavian ice sheet in southern England [63] and Arctic Russia [64], but they are likely influenced by wind pollen transport from more distant sites. This is indicated by a low concentration of *Alnus* pollen and the presence of steppe taxa in pollen profiles [64], [88].

Because of the absence of reliable records from the LGM, we used pollen sites and macrofossils from the Late Glacial and early Holocene periods as indicators of possible *Alnus* LGM refugial areas (Figure 6). These sources indicate the presence of *Alnus* during the LGM in western Europe, the northern foothills of the Alps, the Romanian Carpathians and a large area of northeastern Europe (Figure 6). Evidence of more than 2.5% pollen from sites located in northeastern Europe from the Late Glacial period are generally interpreted as a reworking of earlier climatically favourable periods or long-distance dispersal [72], [73], [74], [75], [76] but macrofossil remains found in Poland, Belarus, Lithuania and Latvia support the occurrence of *Alnus* in this area (Figure 6).

The ability of alder trees to tolerate the climatic conditions of the LGM in northern areas has been supported in several ways. Kullman [39] showed a high tolerance of *Alnus incana* to cold climates by assessing its regeneration patterns in a subalpine forest of central Sweden. He suggested that *A. incana* could have survived the last glacial period in northern areas because it has high vegetative survivability far above its generative limit. Palaeodistributional modelling based on the climatic tolerance of trees has suggested the possible existence of *A. incana* in the proximity of the ice sheet, including southern England, northern France, Central Europe, the northern Carpathians and Belarus, but this modelling has also suggested that *A. incana* was absent from the northwestern Russian plains [7]. The northern occurrence of *A. glutinosa* reached France and the northern foothills of the Alps, but the species was absent from the northern Carpathians, Belarus and the northwestern Russian plains [7]. The survival of *Alnus* species in the North throughout the LGM might be supported by their occurrence in floodplains, which were moister and more sheltered sites than the typical dry habitats of the surrounding uplands with the occurrence of permafrost [13].

### Southern LGM Refugia

Surprisingly, the 2.5% threshold does not support the Mediterranean peninsulas as LGM refugial areas for *Alnus* with the sole exception of the Pyrenees [53]. This finding contradicts the conclusions of a phylogeographic study on *A. glutinosa* that detected specific cpDNA haplotypes for particular southern peninsulas [34]. A recent population-genetic study of Lepais et al. [89], supported also by pollen data [90], [91] highlights the behaviour of rear-edge stable populations of *A. glutinosa* in North Africa. They found that tetraploid *A. glutinosa* populations in Morocco have diverged for a long-time without contribution of gene flow of Algerian or Tunisian diploid populations. This supports to the idea that *Alnus* survived in the Mediterranean area at mesoclimatically favourable sites (e.g., in foothill valleys) in sparse and isolated populations, which are generally hard to detect by pollen analyses [4], which possibly explains the low percentage of *Alnus* pollen.

### Holocene *Alnus* Expansion in Northern Regions

The expansion of *Alnus* began in the Baltic region and Scandinavia between 11 and 10 cal. kyr BP (Figure 3D). The absence of *Alnus* evidence in most of central and northwestern Europe indicates that populations in northeastern Europe were predominant sources for the colonisation of Scandinavia. The delayed expansion of *Alnus* in the British Isles between 10 and 8 cal. kyr (Figure 4A and 4B) appears to have originated in a western European refugium [92], [93], [94], [95] rather than in eastern Europe, as suggested by Huntley and Birks [1]. However, eastern populations, which colonised the Baltic states and Scandinavia, could have spread southwest and mixed with western populations [93]. Synchronously with the rise of *Alnus* in the British Isles, alders expanded in Hercynian Mountains, but it is impossible to tell whether Baltic, Carpathian, Alpine or local alder populations contributed to this expansion (Figure 4A and 4B). Source populations are also unknown for the *Alnus* expansion in the Massif Central and the remaining unoccupied area of France between 7 and 6 cal. kyr BP (Figure 4D).

#### Scale-dependent pattern of alnus expansion

In northern areas, the *Alnus* expansion shows a scale-dependent pattern characterised by a synchronous increase of *Alnus* within individual regions, but with regional differences in the times of the expansion. At the scale of hundreds to a thousand kilometres within individual regions, we recorded little or no directional pattern in the *Alnus* expansion, i.e., sites with *Alnus* evidence initially occurred across the whole region, and then the number of sites increased equally. We recorded this pattern in all northern regions, including the deglaciated area of Scandinavia, corroborating the descriptions of Bennett and Birks [95] for the British Isles and Giesecke et al. [18] for the Baltic area. Such a general absence of spatial coherence of the *Alnus* expansion within large areas seems to be very specific in comparison with the generally observed “stepping stone” character of expansions commonly recorded for other European trees [95]. This pattern suggests that the delayed *Alnus* expansion apparent in some regions was likely a result of environmental limitations rather than the effect of slow colonization.

Giesecke et al. [18] suggested that the climate is an important factor affecting regional differences in the expansion of *Alnus.* Global warming is generally assumed to be a trigger of the rapid *Alnus* expansion that began at the turn of the Late Glacial and Holocene periods [1]. However, an arid climate in some regions could have limited the onset of the *Alnus* expansion. The ecological requirements of *Alnus* and their recent distribution indicate that *Alnus* occurrence significantly declines in areas with an arid climate [37]. *Alnus glutinosa* is currently absent from large, arid areas of the Hungarian, Romanian and Ukrainian lowlands and the Iberian peninsula (http://euforgen.org). Increased oceanicity and rising sea levels after the separation of the British Isles from the continent possibly drove the *Alnus* expansion at approximately 9 cal. kyr BP in the British Isles, as suggested by Godwin [96] and Chambers and Elliott [94]. Similarly, the early *Alnus* expansion in the Baltic area could be accelerated by the large area of the Ancylus Lake (i.e., the Baltic sea).

### *Alnus* Expansion in Southern Peninsulas, the Alps and the Carpathians

In southern regions, *Alnus* began its expansion in the Late Glacial and early Holocene periods. It seems that *Alnus* expanded at individual sites rather than synchronously in whole regions. We assume that the arid climate of the Mediterranean, which was temporarily and spatially variable during the Holocene [97], possibly limited the establishment of new populations and locally caused population decreases. Similarly, a harsh, unstable mountain climate [98], [99] possibly drove a relatively slow expansion in the Alps and Carpathians.

During the second part of the Holocene, between 6 and 0 cal. kyr BP, *Alnus* retreats took place in most regions of Europe (Figure 5). In Scandinavia, the northward-southward direction of its population decrease is positively correlated with climate cooling and ombrogenous peat formation, which are likely the main factors initiating this process [37], [39]. Human activity in floodplains resulting in deforestation could be an additional factor contributing to thinning [100].

### Species-specific History of *Alnus glutinosa* and *A. incana* Based on Macrofossils

Differences in the LGM refugia of *Alnus glutinosa* and *A. incana* could have significantly affected the time of the *Alnus* expansion in particular regions. For example, the earlier *Alnus* expansion in the Baltic area could be an expansion of the more cold-tolerant *A. incana*. Available macrofossils, however, do not support such differences between *A. glutinosa* and *A. incana*, although results may be influenced by their relative scarcity. It seems that both *Alnus* species colonised Scandinavia from the area of the northeastern refugium. Macrofossil evidence of *A. incana* from Netherlands also supports its Late Glacial occurrence in western Europe, i.e., outside its recent range [79] (Figure 3C).

### Drawbacks of the Approach

Different factors may influence the proportion of *Alnus* pollen at individual sites and potentially underestimate or overestimate *Alnus* occurrence in the past. The recorded pollen proportion of species depends on pollen production and dispersal of other species in the vegetation [49]. It has been shown that the occurrence of trees (e.g. *Betula*) in areas with low pollen production, such as borders of tundra and taiga, may be overestimated in comparison to forest zones [49]. Recent studies have shown that the size of sedimentary basins, including bogs and lakes, importantly influences the source area of pollen coming from surrounding vegetation [101], [102]. Small sedimentary basins reflect the composition of surrounding vegetation at the expense of regional vegetation patterns and, thus, may underestimate regional species occurrence [101], [102].

One important factor influencing the representation of *Alnus* pollen is its dispersal ability. *Alnus* has small and light pollen grains (fall speed 0.021 ms^–1^, according to Eisenhut [103]) effectively dispersed by wind over large distances. Studies have shown that *Alnus* pollen may occur in quite high relative quantities (4%) in remote areas thousands of kilometres from its closest occurrence in the vegetation [104]. An unstable climate and strong winds in the last glacial period likely facilitated long-distance dispersal of *Alnus* pollen, biasing pollen records in generally treeless landscapes with low pollen production.

The level of taxonomic resolution of the pollen spectra may bias interpretations when considering occurrences of *Alnus glutinosa* and *A. incana*. May and Lacourse [105] pointed out problems with the identification of three species, *A. rubra* (analogous to *A. glutinosa*), *A. incana* and *A. alnobetula*, in pollen spectra based on a dataset from North America. They concluded that if all three species were present in the vegetation, it would be statistically impossible to determine their pollen at the species level. This makes it difficult to distinguish *A. alnobetula* pollen from the other two and complicates the interpretation of pollen records. In southern Italy and Corsica, *Alnus* pollen records may also include pollen grains of *A. cordata*, which grows there sympatrically with *A. glutinosa* in alluvial habitats. Similarly, we cannot fully exclude the presence of pollen transported over long distances belonging to other species of subgenus *Alnus* such as *A. djavanshirii* Zare, *A. dolichocarpa* Zare, *A. orientalis* Decne. and *A. subcordata* C. A. Mey, all recently growing in the Eastern Mediterranean area and Iran [106], [107]. Despite the above-mentioned facts, the accordance of macrofossils with the pollen records confirms the robustness of the relative pollen data used in this study.

### Comparison with Huntley and Birks, and King and Ferris

Using a much larger pollen dataset, we broadly confirmed LGM refugial areas and the general pattern of the postglacial expansion of *Alnus* as presented in the Huntley and Birks [1] “Pollen Maps”, thus supporting the robustness and actuality of their work. The main differences between our study and the conclusions of Huntley and Birks [1] lie in the interpretation of the importance of northern LGM refugial areas for the *Alnus* expansion. Based on our dataset, the refugium in northeastern Europe appears to be more important for the *Alnus* expansion than was proposed by Huntley and Birks [1]. Huntley and Birks [1] mentioned this area only as a possible LGM refugium of *A. incana* subsp. *kolaensis*. We also support that the western refugium rather than eastern European one was the source for the expansion in the British Isles.

King and Ferris [34] have suggested the Carpathians as possible source areas for the expansion of *Alnus glutinosa* in the northern part of Europe. Our study also supports northeastern and western Europe. However, some conclusions of King and Ferris [34] seems to be based on the work of Huntley and Birks [1] rather than on molecular data. Only two largely distributed haplotypes, the first occurring across all northern parts of Europe and the second in the Alps, the Carpathians, western Europe and Scandinavia, were recorded by King and Ferris [34]. The presence of two weakly spatially structured haplotypes in the northern part of Europe may reflect the postglacial expansion of genotypes from the Carpathians [34] but may also correspond to the fragmentation of the continual *A. glutinosa* range during cold phases of the last glacial period. Similarly, some tree species most likely surviving the last glacial period in the northern part of Europe, such as *Betula pendula*, *B. pubescens*, *Populus tremula* and *Salix caprea*, exhibit a low level of phylogeographic structure [108], [109], [110]. To shed light on the last glacial period and Holocene history of *A. glutinosa* in northern Europe, future molecular studies should combine several approaches. For example, more variable chloroplast DNA markers [111] and microsatellites capable of determining the demographic history of *A. glutinosa* [112] in a particular region using approximate Bayesian computation [113] could be employed. A similar study is needed for *A. incana*, for which molecular studies are still missing.

Huntley and Birks [1] postulated two questions concerning the expansion pattern of *Alnus* in Europe. First, they asked why *Alnus* delayed its expansion north of the Alps. They hypothesised that this delay could have been caused by the occurrence of only cold-demanding *A*. *incana* and *A*. *alnobetula* in the Alpine LGM refugium. These species were unable to colonise the upland and lowland areas north of the Alps. This answer remains plausible, but the macrofossil finding of *A. glutinosa* in the southern foothills of the Alps in the Late Glacial period makes their interpretations less probable. Second, they posed a question about the importance of a western refugium for the *Alnus* expansion, which appears to be the source for the *Alnus* expansion in the British Isles in our study. However, only future phylogeographic studies can bring progress towards answering the following additional questions: (i) Are there any distinctions among northern LGM refugial areas of *A. glutinosa* and *A. incana* that could influence regional differences at the beginning of the *Alnus* expansion? (ii) Was Scandinavia colonised only from the northeastern refugium, or were there other sources of colonisation located, for example, in western Europe? (iii) What is the origin of *A. incana* subsp. *kolaensis*, whose range has recently been limited to the north of Scandinavia? Within this context, the large area of northwestern Russia and the Baltic states appears to be crucial for future molecular sampling.

## Supporting Information

Figure S1 Holocene distribution (6–2 cal. kyr BP) of *Alnus* pollen sites. According to four classes of percentage of *Alnus* pollen and macrofossil remains. The colour of dots indicates changes compared to the previous period; red, expansion, *Alnus* pollen <2.5% in preceding period; blue, retreat, *Alnus* pollen ≥2.5% in preceding period; orange, new pollen sites of *Alnus* pollen ≥2.5%, respectively; black, stability; the course of deglaciation (white) and changes in coastline (dot lines).
(DOCX)

Figure S2 Holocene distribution (2–0 cal. kyr BP) of *Alnus* pollen sites. According to four classes of percentage of *Alnus* pollen and macrofossil remains; for details see Figure S1.
(DOCX)

Table S1 Location of the pollen sites from EPD, PALYCZ and the literature (Lit.).
(DOCX)

Table S2 Location of the macrofossil sites from NEMD and the literature (Lit.).
(DOCX)

Table S3 References of the pollen and macrofossil sites from EPD, PALYCZ, NEMD and the literature (Lit.).
(DOCX)

Checklist S1 PRISMA checklist.
(DOCX)
