# Supplementary material for: Migration Patterns of Subgenus Alnus in Europe since the Last Glacial Maximum: A Systematic Review
Source: PLoS One. 2014 Feb 21;9(2):e88709. doi: 10.1371/journal.pone.0088709 (PMC3931649; doi:10.1371/journal.pone.0088709)
Supplement: Figure S1 — Holocene distribution (6–2 cal. kyr BP) of Alnus pollen sites. According to four classes of percentage of Alnus pollen and macrofossil remains. The colour of dots indicates changes compared to the previous period; red, expansion, Alnus pollen <2.5% in preceding period; blue, retreat, Alnus pollen ≥2.5% in preceding period; orange, new pollen sites of Alnus pollen ≥2.5%, respectively; black, stability; the course of deglaciation (white) and changes in coastline (dot lines). (DOCX) [file pone.0088709.s001.docx]

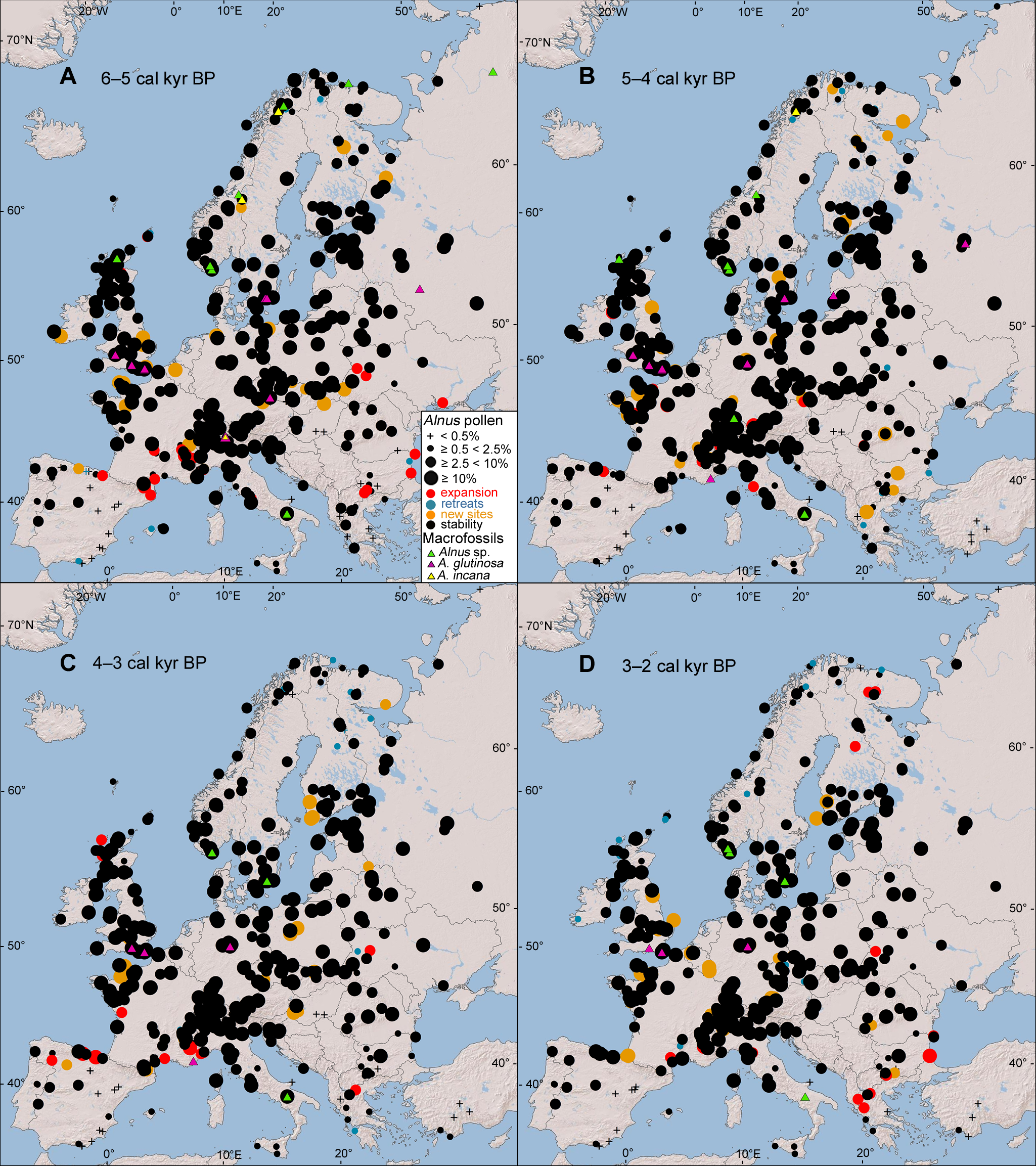


**Figure S1. Holocene distribution (6–2 cal. kyr BP) of *Alnus* pollen sites.** According to four classes of percentage of *Alnus* pollen and macrofossil remains. The colour of dots indicates changes compared to the previous period; red, expansion, *Alnus* pollen < 2.5% in preceding period; blue, retreat, *Alnus* pollen ≥ 2.5% in preceding period; orange, new pollen sites of *Alnus* pollen ≥ 2.5%, respectively; black, stability; the course of deglaciation (white) and changes in coastline (dot lines).
