# Supplementary material for: Migration Patterns of Subgenus Alnus in Europe since the Last Glacial Maximum: A Systematic Review
Source: PLoS One. 2014 Feb 21;9(2):e88709. doi: 10.1371/journal.pone.0088709 (PMC3931649; doi:10.1371/journal.pone.0088709)
Supplement: Figure S2 — Holocene distribution (2–0 cal. kyr BP) of Alnus pollen sites. According to four classes of percentage of Alnus pollen and macrofossil remains; for details see Figure S1. (DOCX) [file pone.0088709.s002.docx]

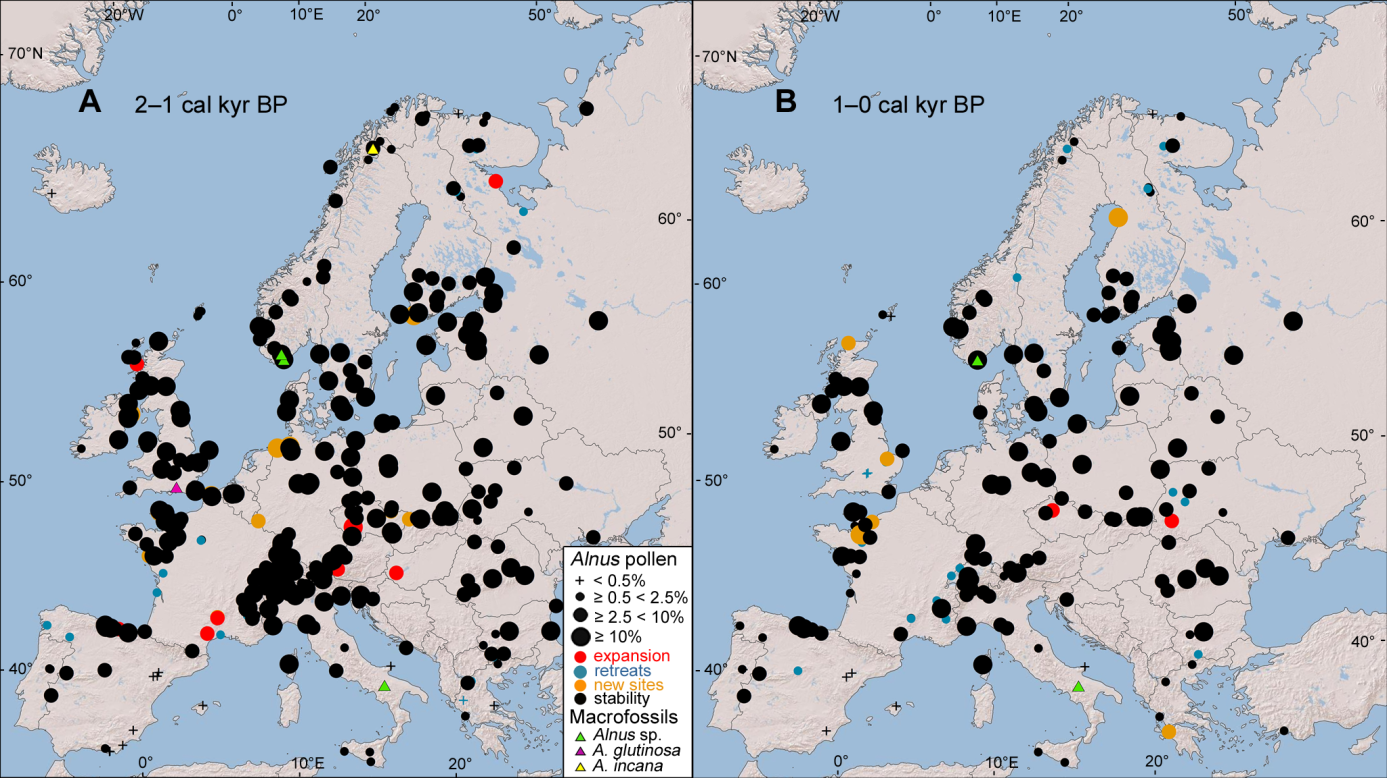


**Figure S2. Holocene distribution (2–0 cal. kyr BP) of *Alnus* pollen sites.** According to four classes of percentage of *Alnus* pollen and macrofossil remains; for details see Appendix S3 Figure S1.
