# Supplementary material for: Migration Patterns of Subgenus Alnus in Europe since the Last Glacial Maximum: A Systematic Review
Source: PLoS One. 2014 Feb 21;9(2):e88709. doi: 10.1371/journal.pone.0088709 (PMC3931649; doi:10.1371/journal.pone.0088709)
Supplement: Table S1 — Location of the pollen sites from EPD, PALYCZ and the literature (Lit.). (DOCX) [file pone.0088709.s003.docx]

**Table S1. Location of the pollen sites from EPD, PALYCZ and the literature (Lit.).**

|  | ***Country*** | ***Longitude*** | ***Latitude*** | ***Source*** | ***Reference*** |
| --- | --- | --- | --- | --- | --- |
| ***Site*** |  |  |  |  |  |
| Lake Maliq | ALB | 40.35 | 20.42 | Lit. | [1] |
| Atemlöchermoos | AUT | 46.94 | 11.2 | EPD | [2] |
| Buntes Moor | AUT | 47.06 | 11.30 | EPD | [3] |
| Dortmunder Hütte | AUT | 47.10 | 11.00 | EPD | [4], [5] |
| Egelsee | AUT | 47.61 | 12.17 | EPD | [6] |
| Franz Senn-Hütte | AUT | 47.35 | 11.88 | EPD | [3] |
| Fuschlsee | AUT | 47.78 | 13.27 | EPD | [7] |
| Gerlos | AUT | 47.24 | 12.14 | EPD | [2] |
| Giering | AUT | 47.47 | 12.36 | EPD | [8] |
| Gradenmoos | AUT | 47.97 | 12.81 | EPD | [9] |
| Hasenmoos | AUT | 47.47 | 12.38 | EPD | [8] |
| Katzenloch | AUT | 47.34 | 11.13 | EPD | [6] |
| Kirchbichl | AUT | 47.51 | 12.9 | EPD | [6] |
| Lanser Moor | AUT | 47.24 | 11.42 | EPD | [2] |
| Lindenmoos | AUT | 47.51 | 12.4 | EPD | [2] |
| Lutzenberg | AUT | 47.46 | 12.36 | EPD | [8] |
| Mieminger See | AUT | 47.29 | 10.98 | EPD | [6] |
| Miesberg | AUT | 47.65 | 12.27 | EPD | [8] |
| Moor Alpenrose | AUT | 47.09 | 11.78 | EPD | [3] |
| Moor am Rofenberg | AUT | 46.83 | 10.83 | EPD | [10] |
| Pillermoos Untergurgl | AUT | 46.90 | 11.4 | EPD | [2] |
| Rotmoos Obergurgl | AUT | 46.84 | 11.3 | EPD | [2] |
| Schönwies | AUT | 46.85 | 11.3 | EPD | [2] |
| Schwarzsee, Reschenscheideck | AUT | 46.87 | 10.48 | EPD | [11], [12] |
| Schwemm | AUT | 47.65 | 12.30 | EPD | [13] |
| Tauernmoos | AUT | 47.17 | 12.64 | EPD | [14] |
| Trumer Moos | AUT | 47.93 | 13.7 | EPD | [15] |
| Wasenmoos beim Zellhof | AUT | 47.98 | 13.10 | EPD | [15] |
| Waxeckalm | AUT | 47.02 | 11.50 | EPD | [4], [5] |
| Wildmoos | AUT | 46.95 | 11.2 | EPD | [2] |
| Zirbenwaldmoor | AUT | 46.86 | 11.3 | EPD | [16] |
| Bois des Amerois | BLR | 49.74 | 5.13 | EPD | [17] |
| Ivanisovka peat bog | BLR | 52.27 | 26.5 | Lit. | [18] |
| Lake Bobrovichskoe | BLR | 52.62 | 25.78 | Lit. | [18] |
| Lake Lozoviki | BLR | 55.27 | 28.12 | Lit. | [18] |
| Lake Mezhuzhol | BLR | 55.00 | 28.7 | Lit. | [18] |
| Lake Neropla | BLR | 53.73 | 29.88 | Lit. | [18] |
| Moerzeke | BEL | 51.05 | 4.18 | EPD | [19], [20] |
| Snellegem | BEL | 51.19 | 3.28 | EPD | [20], [21], [22] |
| Vinderhoute | BEL | 51.08 | 3.62 | EPD | [20], [21] |
| Uitbergen | BEL | 51.02 | 3.94 | EPD | [19] |
| Arkutino Lake | BGR | 42.37 | 27.73 | EPD | [23] |
| Beliya Kanton | BGR | 41.73 | 24.13 | EPD | [24] |
| Besbog | BGR | 41.75 | 23.67 | EPD,Lit | [25], [26] |
| Black Sea (South) | BGR | 42.07 | 28.49 | EPD | [27] |
| Dry Lake II | BGR | 42.05 | 23.53 | EPD | [28], [29] |
| Ezero wetland, Thracia plain | BGR | 48.47 | 26.2 | Lit. | [30] |
| Kupena | BGR | 41.98 | 24.33 | EPD | [31] |
| Lake Dalgoto | BGR | 41.67 | 23.37 | Lit. | [32] |
| Lake Duranunlak | BGR | 43.67 | 28.55 | EPD | [33], [34] |
| Lake Ribno Banderishko | BGR | 41.84 | 23.30 | Lit. | [35] |
| Lake Sedmo Rilsko | BGR | 42.19 | 23.58 | EPD | [36] |
| Lake Shabla-Ezeretz | BGR | 43.58 | 28.55 | EPD | [34], [37] |
| Lake Srebarna | BGR | 44.08 | 27.12 | EPD | [38] |
| Lake Varna (Arsenala) | BGR | 43.20 | 27.83 | EPD | Bozilova E (unpublished) |
| Lake Varna (Beloslav-Poveljanovo) | BGR | 43.20 | 27.83 | EPD | Bozilova E (unpublished) |
| Maleshevska Mountains Peat Bog | BGR | 41.70 | 23.3 | EPD | [39], [40] |
| Mire Garvan | BGR | 44.12 | 26.95 | EPD | [38] |
| Mozgovista | BGR | 41.71 | 23.47 | Lit. | [41] |
| Mutorog Peat Bog | BGR | 43.52 | 23.62 | EPD | [42], [43] |
| Pirin, Lake Kremensko-5 | BGR | 41.72 | 23.53 | Lit. | [26], [44] |
| Popovo Ezero | BGR | 41.72 | 23.67 | EPD | [25] |
| Sredna Gora Mountains Peat Bog | BGR | 42.83 | 24.83 | EPD | [45], [46], [47] |
| Trilistnika, Seven Rila Lakes | BGR | 42.20 | 23.32 | Lit. | [48] |
| Tschokljovo Marsh | BGR | 42.37 | 22.83 | EPD | [39], [49], [50] |
| Vitosha Mountains Peat Bog | BGR | 42.83 | 23.83 | EPD | [51], [52] |
| Dolgoe | BLR | 55.23 | 28.18 | EPD | [53] |
| Chernikhovo | BLR | 53.42 | 26.43 | EPD | [54], [55] |
| Novolsky | BLR | 56.77 | 26.18 | EPD | [55], [56] |
| Oltush Lake | BLR | 51.70 | 23.96 | EPD | Yelovicheva YaK (unpublished) |
| Osvea | BLR | 56.05 | 28.8 | EPD | [53] |
| Sudoble Lake | BLR | 54.03 | 28.10 | EPD | [57], [58] |
| Anenské údolí | CZE | 50.59 | 16.12 | PALYCZ | [59] |
| Blato | CZE | 49.04 | 15.19 | EPD | [60], [61], [62] |
| Borkovicka blata | CZE | 49.22 | 14.90 | EPD | [63] |
| Dvůr Anšov | CZE | 48.78 | 16.42 | PALYCZ | [64] |
| Flaje Kiefern | CZE | 50.70 | 13.53 | EPD | [65], [66], [67], [68], [69], [70], [71], [72], [73], [74], [75] |
| Hala Izerska | CZE | 50.85 | 15.36 | PALYCZ | Baranowska-Kącka A (unpublished) |
| Horní Lomná | CZE | 49.52 | 18.63 | PALYCZ | [76] |
| Hrabanovská černava | CZE | 50.22 | 14.83 | PALYCZ | [77] |
| Hůrecká slať | CZE | 49.15 | 13.33 | PALYCZ | [78] |
| Chrást | CZE | 50.26 | 14.54 | PALYCZ | [79] |
| Jablůnka | CZE | 49.38 | 17.95 | PALYCZ | [80] |
| Jelení louže | CZE | 50.89 | 14.28 | PALYCZ | [59] |
| Kamenicky | CZE | 49.73 | 15.97 | EPD | [81], [82] |
| Knížecí pláně | CZE | 48.96 | 13.64 | PALYCZ | [83] |
| Kožlí | CZE | 49.36 | 14.2 | PALYCZ | [84] |
| Krkonoše | CZE | 50.76 | 15.55 | Lit. | [85] |
| Krušné hory | CZE | 54.69 | 13.62 | Lit. | [75] |
| Labský důl | CZE | 50.77 | 15.55 | Lit. | [86] |
| Loucky | CZE | 49.33 | 15.50 | EPD | [62] |
| Malá Niva | CZE | 48.87 | 13.88 | Lit. | [78] |
| Mokre louky (South) | CZE | 48.83 | 14.83 | EPD | [87] |
| Nad Dolským mlýnem | CZE | 50.85 | 14.34 | PALYCZ | [88] |
| Olbramovice | CZE | 48.99 | 16.40 | EPD | [89] |
| Palasiny | CZE | 49.69 | 15.48 | EPD | [90] |
| Plešné jezero | CZE | 48.78 | 13.87 | PALYCZ | [91] |
| Podbaba | CZE | 50.11 | 14.39 | Lit. | [80] |
| Rasna | CZE | 49.23 | 15.37 | EPD | [62] |
| Rejvíz | CZE | 50.23 | 17.31 | Lit. | [92] |
| Rezabinec | CZE | 49.25 | 14.12 | EPD | [93] |
| Rybárenská Slať | CZE | 48.96 | 13.64 | Lit. | [78] |
| Rynholec | CZE | 50.13 | 13.97 | Lit. | [94] |
| Stráženská slať | CZE | 48.96 | 13.64 | Lit. | [83] |
| Svatoborice-Mistrin | CZE | 48.83 | 17.17 | EPD | [95] |
| Svatobořice | CZE | 48.95 | 17.8 | PALYCZ | [95] |
| Švarcenberk | CZE | 49.15 | 14.70 | Lit. | [96] |
| Teplické údolí | CZE | 50.58 | 16.13 | PALYCZ | [97] |
| Velky Ded | CZE | 50.08 | 17.22 | EPD | Rybníčková E (unpublished) |
| Velky Maj | CZE | 50.05 | 17.22 | EPD | Rybníčková E (unpublished) |
| Vernerovice | CZE | 50.10 | 16.25 | EPD | [98] |
| Vlčí rokle | CZE | 50.60 | 16.13 | PALYCZ | [97] |
| Vracov | CZE | 48.98 | 17.20 | EPD | [89], [99], [100], Sladkova-Hynkeva H (unpublished) |
| Weiherlohe | CZE | 49.73 | 12.39 | PALYCZ | [101] |
| Weissenstadter Forst | CZE | 50.14 | 11.88 | PALYCZ | [102] |
| Windbruch | CZE | 49.61 | 12.54 | PALYCZ | [101] |
| Wolfslohe | CZE | 49.91 | 12.4 | PALYCZ | [102] |
| Zbudovska blata | CZE | 49.83 | 14.33 | EPD | [103], [104] |
| Esbjerg area | DEN | 55.52 | 8.38 | Lit. | [105] |
| Præstesti site | DEN | 55.52 | 8.38 | Lit. | [105] |
| Vrogum | DEN | 55.68 | 8.23 | Lit. | [106] |
| Ahlenmoor | DEU | 53.70 | 8.73 | EPD | [107], [108] |
| Ahlequellmoor | DEU | 51.73 | 9.51 | EPD | [109] |
| Bibersee | DEU | 47.21 | 8.47 | Lit. | [110] |
| Breitnau-Neuhof | DEU | 47.93 | 8.7 | EPD | [111] |
| Bruchberg | DEU | 51.76 | 10.46 | EPD | [112], [113] |
| Dunum (Hilliges Moor) | DEU | 53.58 | 7.63 | EPD | [108], [114] |
| Durchenbergried | DEU | 47.78 | 8.98 | EPD | [115], [116] |
| Felchosee | DEU | 53.05 | 14.13 | EPD | [117] |
| Feuenried | DEU | 47.75 | 8.92 | EPD | [116] |
| Flögeln | DEU | 53.67 | 8.76 | EPD | [107], [108] |
| Georgenfelder Hochmoor | DEU | 50.75 | 13.75 | EPD | [73] |
| Großer Krebssee | DEU | 52.85 | 14.10 | EPD | [117] |
| Glaswaldsee | DEU | 48.43 | 8.25 | EPD | [118], [119] |
| Herthamoor | DEU | 54.57 | 13.65 | Lit. | [120] |
| Huzenbacher See | DEU | 48.58 | 8.34 | EPD | [121], [122] |
| Jues Lake | DEU | 51.66 | 10.35 | Lit. | [123] |
| Kleinen Mochowsee | DEU | 52.00 | 14.20 | EPD | [124] |
| Krumpa | DEU | 51.30 | 11.85 | EPD | [125], [126] |
| Langes Fenn Kemnitzerheide | DEU | 52.31 | 12.91 | EPD | [127], [128] |
| Löddigsee | DEU | 53.43 | 11.85 | EPD | [129] |
| Lüderholz | DEU | 51.68 | 10.31 | EPD | [130] |
| Lüttersee | DEU | 51.58 | 10.16 | EPD | [130] |
| Mecklenburg | DEU | 53.43 | 11.85 | Lit. | [129] |
| Merzdorf | DEU | 51.40 | 13.53 | EPD | [131] |
| Moor im Weissenstadter Forst | DEU | 50.14 | 11.88 | EPD | [102] |
| Neuenhagener Oderinsel | DEU | 53.05 | 14.13 | Lit. | [117] |
| Oberderdingen-Großvillars | DEU | 49.04 | 8.76 | EPD | [132] |
| Ober-Hörgern 2 | DEU | 50.46 | 8.75 | Lit. | [133] |
| Plinz | DEU | 50.83 | 11.50 | Lit. | [134] |
| Salzwise | DEU | 52.15 | 9.91 | Lit. | [120] |
| Sauborst | DEU | 50.27 | 12.3 | Lit. | [102] |
| Säulingsee | DEU | 50.93 | 9.98 | Lit. | [120] |
| Seelohe | DEU | 50.26 | 12.5 | Lit. | [102] |
| Silberhohl | DEU | 51.91 | 10.18 | EPD | [130] |
| Sonnenberger Moor | DEU | 51.77 | 10.52 | EPD | [112] |
| Steerenmoos | DEU | 47.80 | 8.20 | EPD | [135] |
| Steisslingen lake | DEU | 47.80 | 8.90 | Lit. | [136] |
| Unter-Ückersee | DEU | 53.25 | 13.85 | EPD | [137] |
| Wachel 3 | DEU | 53.44 | 8.87 | EPD | [138] |
| Waschhorn | DEU | 53.62 | 8.74 | EPD | [139] |
| Wilder See beim Ruhestein | DEU | 48.57 | 8.24 | EPD | [118], [119] |
| Wildseemoor bei Kaltenbronn | DEU | 48.72 | 8.46 | EPD | [118] |
| Wissenstadter forst | DEU | 50.13 | 11.88 | Lit. | [102] |
| Wolfslohe | DEU | 49.80 | 11.55 | Lit. | [102] |
| Lake Solso | DNK | 56.13 | 8.63 | EPD | [140] |
| Albufera Alcudia | ESP | 39.79 | 3.12 | EPD | [141] |
| Algendar | ESP | 39.94 | 3.96 | EPD | [142], [143] |
| Alsa | ESP | 43.12 | -4.02 | EPD | [144] |
| Antas | ESP | 37.21 | -1.82 | EPD | [142], [145], [146] |
| Atxuri | ESP | 43.25 | -1.55 | EPD | [147], [148] |
| Banyoles | ESP | 42.13 | 2.75 | EPD | [149] |
| Cala Galdana | ESP | 39.94 | 3.97 | EPD | [142] |
| Cala'n Porter | ESP | 39.87 | 4.13 | EPD | [142] |
| Canada de la Cruz | ESP | 38.07 | -1.30 | Lit. | [150] |
| Canal de Navarrés | ESP | 39.08 | -0.69 | Lit. | [151] |
| Carihuela Cave | ESP | 37.45 | -2.57 | Lit. | [152] |
| Cova Beneito | ESP | 38.80 | -0.47 | Lit. | [153] |
| Cueto de la Avellanosa | ESP | 43.12 | -4.36 | EPD | [154] |
| El Jardin | ESP | 38.00 | -1.00 | Lit. | [155] |
| Gallocanta Lake | ESP | 40.98 | -0.50 | Lit. | [156] |
| Hoya del Castillo | ESP | 41.25 | -0.50 | EPD | [157] |
| Hoyos de Iregua | ESP | 40.90 | -3.88 | Lit. | [158] |
| Lago de Ajo | ESP | 43.05 | -6.15 | EPD | [159], [160] |
| Lagoa de Lucenza | ESP | 42.58 | -6.88 | Lit. | [161] |
| Laguna de la Roya | ESP | 42.22 | -6.77 | EPD | [160] |
| Laguna de las Sanguijuelas | ESP | 42.13 | -5.30 | Lit. | [162] |
| Laguna Guallar | ESP | 41.40 | -0.22 | EPD | [157] |
| Laguna Salada Chiprana | ESP | 41.23 | -0.17 | EPD | Davis BAS (unpublished) |
| Las Devotas | ESP | 42.58 | -5.80 | Lit. | [163] |
| Las Pardillas Lake | ESP | 42.05 | -3.05 | EPD | [164] |
| Montes do Buio Cuadramón | ESP | 43.47 | -7.53 | EPD | [165] |
| Navarrés | ESP | 39.10 | -0.68 | EPD | [151], [166] |
| Ojos del Tremedal, Teruel | ESP | 40.54 | -1.95 | Lit. | [167] |
| Pico del Sertal | ESP | 43.22 | -4.44 | EPD | [168] |
| Pozo do Carballal | ESP | 42.71 | -6.89 | Lit. | [169] |
| PRD-4 | ESP | 42.53 | -8.52 | EPD | [170] |
| Puerto de Belate | ESP | 43.03 | -2.05 | EPD | [147], [148] |
| Puerto de las Estaces de Trueba | ESP | 43.12 | -3.70 | EPD | [171] |
| Puerto de Los Tornos | ESP | 43.15 | -3.43 | EPD | [147] |
| Quintanar de la Sierra | ESP | 42.03 | -3.02 | EPD | [147], [148] |
| Rascafria | ESP | 40.90 | -3.88 | Lit. | [172] |
| Roquetas de Mar | ESP | 36.79 | -2.59 | EPD | [142], [145], [146], [173] |
| Salada Pequeña | ESP | 41.03 | -0.22 | EPD | Davis BAS (unpublished) |
| Saldropo | ESP | 43.05 | -2.72 | EPD | [147], [148] |
| San Rafael | ESP | 36.77 | -2.60 | EPD | [142], [145], [174] |
| Sanabria Marsh | ESP | 42.10 | -6.73 | EPD | [160], [175], [176] |
| Sierra de Gádor | ESP | 36.90 | -2.92 | Lit. | [177] |
| Tramacastilla | ESP | 42.73 | -0.40 | Lit. | [163] |
| Urdaibai | ESP | 43.32 | -1.30 | Lit. | [178] |
| Åntu Sinijarv | EST | 59.13 | 26.33 | EPD | [179] |
| Haljala | EST | 59.42 | 26.30 | Lit. | [180] |
| Imatu Mire | EST | 59.13 | 27.43 | EPD | Saarse L (unpublished) |
| Kalsa Mire | EST | 58.17 | 27.45 | EPD | [181] |
| Kirikumae | EST | 57.67 | 27.25 | EPD | [182] |
| Lake Ermistu | EST | 58.37 | 23.97 | EPD | [183] |
| Lake Karujarv | EST | 58.38 | 22.20 | EPD | [184], [185] |
| Lake Maardu | EST | 59.43 | 25.00 | EPD | [186] |
| Lake Ruila | EST | 59.17 | 24.43 | Lit. | [187] |
| Lake Viitna Linajärv | EST | 59.45 | 26.00 | Lit. | [188] |
| Lasva | EST | 57.86 | 27.18 | Lit. | [189] |
| Liivjarve Bog | EST | 59.22 | 27.58 | EPD | [190] |
| Mire Johvika | EST | 58.50 | 22.33 | EPD | [191] |
| Mire Pelisoo | EST | 58.47 | 22.38 | EPD | [184] |
| Mire Saviku | EST | 58.40 | 27.23 | EPD | [192] |
| Påidre | EST | 58.27 | 25.63 | EPD | [193] |
| Plaani Külajärv Lake | EST | 57.68 | 27.8 | Lit. | [189], [194] |
| Punso | EST | 57.68 | 27.25 | EPD | Saarse L (unpublished) |
| Raigastvere Lake | EST | 58.60 | 26.67 | EPD | [195] |
| Tondi | EST | 59.47 | 24.92 | EPD | [179], [196] |
| Verijärv | EST | 57.81 | 27.6 | Lit. | [189] |
| Vôhma Mire | EST | 59.05 | 27.33 | EPD | Saarse L (unpublished) |
| Aholami | FIN | 61.88 | 25.22 | EPD | Koivula L (unpublished) |
| Akuvaara | FIN | 69.13 | 27.68 | EPD | [197] |
| Hirvilampi | FIN | 60.63 | 24.25 | EPD | [198] |
| Iso Lehmälampi lake | FIN | 60.35 | 24.60 | Lit. | [199] |
| Isokärret | FIN | 60.22 | 22.13 | EPD | [200] |
| Kaarkotinlampi | FIN | 61.42 | 25.87 | EPD | [201] |
| Kaartlamminsuo | FIN | 60.73 | 24.22 | EPD | [198] |
| Kangerjoki | FIN | 66.12 | 29.00 | EPD | [202] |
| Kirkkosaari | FIN | 60.87 | 24.50 | EPD | [198] |
| Kittilä | FIN | 65.03 | 24.68 | EPD | [203], [204], [205] |
| Konilampi mire | FIN | 61.80 | 24.28 | Lit. | [206] |
| Kuivajarvi | FIN | 60.78 | 23.83 | EPD | Vuerela I (unpublished) |
| Lake Kolmilaträsk | FIN | 60.28 | 20.15 | EPD | [208] |
| Lake Njargajavri | FIN | 69.87 | 27.17 | Lit. | [209] |
| Lake Skaidejavri | FIN | 70.00 | 27.87 | EPD | [210] |
| Lake Somaslampi | FIN | 69.26 | 21.51 | Lit. | [211] |
| Lake Tsuolbmajavri | FIN | 68.69 | 22.8 | EPD | [212], [213] |
| Lalaxkärret | FIN | 60.15 | 21.87 | EPD | [214] |
| Maanselänsuo | FIN | 65.62 | 29.60 | EPD | [215] |
| Masehjavri | FIN | 69.05 | 20.98 | EPD | [216] |
| Mossen | FIN | 60.12 | 21.60 | EPD | [214] |
| Mukkavaara | FIN | 68.92 | 21.00 | EPD | [216], [217] |
| Rukatunturi | FIN | 66.17 | 29.15 | EPD | [218], [219] |
| Ryönänsuo | FIN | 60.43 | 24.17 | EPD | [198] |
| Särkikangas | FIN | 65.92 | 29.20 | EPD | [219], [220], [221] |
| Siikasuo | FIN | 61.30 | 22.7 | EPD | [222] |
| Sipola | FIN | 65.05 | 24.79 | EPD | Hicks S (unpublished) |
| Suovalampi | FIN | 69.58 | 28.83 | EPD | [197] |
| Syrjälänsuo | FIN | 61.22 | 28.12 | EPD | Vuorela I (unpublished) |
| Tullerinsuo | FIN | 61.33 | 21.95 | EPD | [222] |
| Vasikkasuo | FIN | 64.67 | 27.87 | EPD | [214], [222] |
| Viheriäisenneva mire | FIN | 61.85 | 24.23 | Lit. | [206] |
| Ylimysneva | FIN | 62.13 | 22.87 | EPD | [223] |
| Altenweiher | FRA | 48.01 | 6.99 | EPD | [224] |
| Ampoix | FRA | 45.63 | 2.93 | EPD | [225], [226] |
| Anneray | FRA | 49.16 | -0.06 | EPD | Clet-Pellerin M (unpublished) |
| Anse de Gattemare | FRA | 49.69 | -1.30 | EPD | [227], [228] |
| Anse de la Mare | FRA | 49.63 | -1.23 | EPD | [227], [228] |
| Anse Saint-Martin | FRA | 49.70 | -1.88 | EPD | [229] |
| Aronde | FRA | 49.46 | 2.69 | EPD | Gauthier E (unpublished) |
| Auneau | FRA | 48.46 | 1.79 | EPD | Richard H (unpublished) |
| Badcére | FRA | 42.59 | 2.6 | Lit. | [230] |
| Baie de Seine estuary | FRA | 49.42 | -0.03 | EPD | [231], [232], [233] |
| Basse-Ville | FRA | 47.19 | -1.86 | EPD | [234] |
| Bellefontaine | FRA | 46.58 | 6.9 | EPD | [235] |
| Bellengreville | FRA | 49.12 | -0.22 | EPD | Clet-Pellerin M (unpublished) |
| Biot | FRA | 43.80 | 7.10 | EPD | [236] |
| Cairon | FRA | 49.24 | -0.45 | EPD | [237], [238], [239] |
| Clapeyret | FRA | 44.15 | 7.24 | EPD | [240] |
| Col des Lauzes | FRA | 46.04 | 6.54 | EPD | [240] |
| Col du Pré | FRA | 45.71 | 6.61 | Lit. | [241] |
| Col Luitel | FRA | 45.09 | 5.85 | EPD | [242] |
| Correo | FRA | 44.51 | 5.98 | EPD | [243] |
| Coulvain | FRA | 49.07 | -0.72 | EPD | Clet-Pellerin M (unpublished) |
| Cristol Lake | FRA | 45.00 | 6.63 | EPD | [243] |
| Embouchac | FRA | 43.57 | 3.92 | EPD | [244], [245] |
| Ennerie | FRA | 47.24 | -2.00 | EPD | [246] |
| Etang de Cheylade | FRA | 45.09 | 2.90 | EPD | [247] |
| Etang d'Ouveillan | FRA | 43.27 | 3.00 | EPD | [235] |
| Etang du Lautrey | FRA | 46.59 | 5.86 | EPD | [229] |
| Etang paysan | FRA | 49.70 | -1.87 | EPD | [248] |
| Fangeas | FRA | 44.72 | 6.45 | EPD | [237] |
| Fontaine Henry | FRA | 49.28 | -0.45 | EPD | [237], [238], [239] |
| Fougères | FRA | 48.52 | -0.83 | EPD | [249] |
| Fournas | FRA | 42.70 | 2.6 | Lit. | [230] |
| Gourg Négre | FRA | 42.63 | 2.22 | Lit. | [230] |
| Grande Brière | FRA | 47.37 | -2.25 | EPD | [250] |
| Hières sur Amby | FRA | 45.79 | 5.28 | EPD | [251] |
| Change-Glatinier | FRA | 48.12 | -0.79 | EPD | [252] |
| Chantemerle | FRA | 45.92 | 0.02 | EPD | [253] |
| Chef-du-Pont | FRA | 49.38 | -1.36 | EPD | [254] |
| La Beuffarde | FRA | 46.82 | 6.42 | EPD | [255] |
| La Borde | FRA | 42.53 | 2.8 | Lit. | [230] |
| La Caudelais | FRA | 47.26 | -1.78 | EPD | [234] |
| La Moulinasse 4 | FRA | 42.69 | 2.24 | Lit. | [230] |
| La Moulinasse 6 | FRA | 42.69 | 2.24 | Lit. | [230] |
| La Taphanel | FRA | 45.27 | 2.68 | EPD | [256] |
| La Vie | FRA | 48.55 | -0.26 | EPD | [252] |
| Lac de Creno | FRA | 42.20 | 8.95 | Lit. | [257], [258] |
| Lac de Praver | FRA | 45.07 | 5.86 | EPD | [243] |
| Lac des Boites | FRA | 45.06 | 5.89 | EPD | [243] |
| Lac du Bouchet | FRA | 44.92 | 3.78 | EPD | [259], [260] |
| Lac du Lauzon | FRA | 44.68 | 5.79 | EPD | [261] |
| Lac Long Inférieur | FRA | 44.06 | 7.45 | EPD | [240] |
| Lac Miroir | FRA | 44.64 | 6.79 | EPD | [243] |
| Lac Mouton | FRA | 44.06 | 7.44 | EPD | [240] |
| Lac Saint Léger | FRA | 44.42 | 6.34 | EPD | [240] |
| Lake of Annecy | FRA | 45.85 | 6.17 | EPD | [262] |
| Laurenti | FRA | 42.68 | 2.3 | Lit. | [230] |
| Lavau | FRA | 47.31 | -1.97 | EPD | [234] |
| Le Fourneau | FRA | 48.44 | -0.19 | EPD | [252] |
| Le Grand Etang de Suze-La-Rousse | FRA | 44.35 | 5.23 | EPD | [263] |
| Le Grand Lemps | FRA | 45.47 | 5.42 | EPD | [251] |
| Le Marais St Boetien | FRA | 49.62 | 3.82 | EPD | [264] |
| Le Suc | FRA | 44.38 | 2.90 | EPD | [265] |
| Le Vivier | FRA | 45.67 | 6.41 | Lit. | [241] |
| Les Etelles | FRA | 45.57 | 6.27 | Lit. | [241] |
| Les Sagnes de Balcere | FRA | 42.59 | 2.6 | Lit. | [230] |
| Lingreville | FRA | 48.93 | -1.54 | EPD | [266] |
| Locmariaquer | FRA | 47.55 | -2.93 | EPD | Visset L (unpublished) |
| Logne | FRA | 47.33 | -1.50 | EPD | Barbier D (unpublished) |
| Loira | FRA | 47.35 | -0.79 | Lit. | [267] |
| Loras | FRA | 45.66 | 5.24 | EPD | [251] |
| Lutinière | FRA | 46.44 | -0.86 | EPD | Visset L (unpublished) |
| Marais de la Dives | FRA | 49.25 | -0.15 | EPD | [268] |
| Marais de Marchesieux | FRA | 49.17 | -1.30 | EPD | [269], [270] |
| Menez-Cam | FRA | 48.25 | -3.50 | EPD | [271], [272] |
| Moselotte | FRA | 48.03 | 7.00 | EPD | [224] |
| Pas du Gu | FRA | 47.24 | -2.15 | EPD | [245] |
| Pelléautier | FRA | 44.52 | 6.18 | EPD | [240] |
| Petiville | FRA | 49.23 | -0.17 | EPD | [268] |
| Peuil Peat Bog | FRA | 45.13 | 5.64 | EPD | [243] |
| Peyrelevade | FRA | 45.71 | 2.38 | EPD | [240] |
| Pierre Folle | FRA | 47.02 | -1.89 | EPD | [245] |
| Pinet | FRA | 42.87 | 1.97 | Lit. | [230] |
| Place du Commerce | FRA | 47.21 | -1.56 | EPD | Visset L (unpublished) |
| Plaine Alpe | FRA | 44.96 | 6.59 | EPD | [273] |
| Plan du Laus | FRA | 44.24 | 6.70 | EPD | [240] |
| Pont-l'Eveque Le Lac | FRA | 49.28 | -0.20 | EPD | San Juan G, Ozouf JC, Clet-Pellerin M (unpublished) |
| Pré Rond | FRA | 44.92 | 6.59 | EPD | [273] |
| Racou mire | FRA | 42.57 | 2.1 | Lit. | [274] |
| Reviers | FRA | 49.30 | -0.47 | EPD | [237], [238] |
| Saint Julien de Ratz | FRA | 45.35 | 5.62 | EPD | [251] |
| Saint Michel de Braspart | FRA | 48.42 | -3.67 | EPD | [271], [272] |
| Saint Sauveur | FRA | 43.57 | 3.92 | EPD | [275] |
| Saint Sixte | FRA | 45.43 | 5.63 | EPD | [251] |
| Saint Viaud Contin | FRA | 47.27 | -2.02 | EPD | [245] |
| Saint-Thomas | FRA | 47.27 | -1.75 | EPD | [234] |
| Saint-Ursin | FRA | 48.52 | -0.25 | EPD | [252] |
| Serrent | FRA | 47.81 | -2.47 | EPD | Visset L (unpublished) |
| St-Momelin | FRA | 50.79 | 2.25 | Lit. | [276] |
| Tocqueboeuf | FRA | 49.69 | -1.42 | EPD | [227], [228] |
| Tourbière de Gatimort | FRA | 43.58 | 2.79 | EPD | [277] |
| Tourbière de la Lande | FRA | 43.57 | 2.97 | EPD | [277] |
| Tourbière de la Peyroutarié | FRA | 44.47 | 3.60 | EPD | [277] |
| Tourbière de Mont Sec | FRA | 45.07 | 5.81 | EPD | [243] |
| Tourbière de Mur de Sologne | FRA | 47.41 | -1.61 | EPD | [278], [279] |
| Tourbière des Narses Mortes | FRA | 44.43 | 3.60 | EPD | [277], [280] |
| Tourbière du Peschio | FRA | 44.45 | 3.60 | EPD | [277] |
| Troarn Saint-Samson | FRA | 49.18 | -0.17 | EPD | [268] |
| Urville-Nacqueville | FRA | 49.68 | -1.76 | EPD | Clet-Pellerin M (unpublished) |
| Vallée de la Voise | FRA | 48.42 | 1.75 | EPD | [264] |
| Vallon de Provence | FRA | 44.39 | 6.40 | EPD | [240] |
| Vauville | FRA | 49.64 | -1.85 | EPD | [281], [282], [283] |
| Watten | FRA | 50.83 | 2.21 | Lit. | [276] |
| Saksunarvatn | FRO | 62.25 | -7.18 | EPD | [284] |
| Abernethy Forest | GBR | 56.23 | -3.72 | EPD | [285] |
| Allt na Feithe Sheilich | GBR | 57.32 | -3.90 | EPD | [286] |
| Arfon Platform | GBR | 53.20 | -3.90 | Lit. | [287] |
| Black Loch, Fife | GBR | 56.21 | -3.15 | Lit. | [288] |
| Bodmin Moor | GBR | 50.53 | -3.47 | Lit. | [289] |
| Brede Bridge | GBR | 50.93 | -0.60 | EPD | [290] |
| Bryn y Castell, Snowdonia | GBR | 52.97 | -3.90 | Lit. | [291] |
| Butter Mountain | GBR | 54.17 | -6.03 | EPD | [292] |
| Cam Loch | GBR | 58.08 | -5.00 | EPD | [293] |
| Caburn | GBR | 50.86 | 0.05 | EPD [294] | |
| Carrivmoragh | GBR | 54.32 | -5.98 | EPD | [292] |
| Clatteringshaws Loch | GBR | 55.07 | -4.28 | EPD | [286] |
| Clettnadal | GBR | 60.05 | -1.36 | Lit. | [295] |
| Coire Bog | GBR | 57.85 | -4.42 | EPD | [286] |
| Cooran Lane | GBR | 55.12 | -4.40 | EPD | [286] |
| Dallican Water | GBR | 60.39 | -1.10 | EPD | [296] |
| Flasks, Vale of Mowbray | GBR | 54.22 | -1.56 | Lit. | [297] |
| Foula | GBR | 60.15 | -2.10 | EPD | [298], [299] |
| Gallanech Beg | GBR | 56.38 | -5.50 | EPD | Davies F (unpublished) |
| Glims Moss | GBR | 59.09 | -3.20 | Lit. | [300] |
| Gors Fawr Bog | GBR | 51.93 | -4.72 | EPD | [301] |
| Hadrians Wall | GBR | 55.09 | -1.49 | Lit. | [302] |
| Hawks Tor | GBR | 50.53 | -4.60 | EPD | [303], [304] |
| Hipper Sick | GBR | 53.22 | -1.58 | EPD | [305], [306], [307] |
| Hobbs Lot March | GBR | 52.58 | 0.08 | EPD | [308] |
| Hockham Mere | GBR | 52.50 | 0.83 | EPD | [309], [310] |
| Horsemarsh Sewer | GBR | 51.05 | -0.80 | Lit. | [311] |
| King's Pool | GBR | 52.81 | -2.11 | EPD | [312] |
| Kinloch, Isle of Rhum | GBR | 58.27 | -4.82 | Lit. | [313] |
| Lackan Bog | GBR | 54.27 | -6.08 | EPD | [292] |
| Lade Bank | GBR | 53.08 | 0.05 | EPD | [308] |
| Lang Lochs Mire | GBR | 60.13 | -1.21 | Lit. | [314] |
| Little Cheyne Court Walland marsh | GBR | 50.96 | 0.83 | EPD | [311] |
| Little Loch Roag | GBR | 58.13 | -6.88 | EPD | [315], [316] |
| Llanilid | GBR | 51.52 | -3.45 | EPD | [317], [318] |
| Llyn Gwernan | GBR | 52.68 | -4.87 | EPD | [319], [320], [321] |
| Loch a´Phuinnd | GBR | 57.37 | -6.73 | Lit. | [322] |
| Loch aBhogaidh | GBR | 55.73 | -5.60 | Lit. | [323] |
| Loch a'Chroisg | GBR | 57.57 | -5.33 | EPD | [324] |
| Loch Ashik | GBR | 57.25 | -5.83 | EPD | [325], [326] |
| Loch Buaillaval | GBR | 58.27 | -5.23 | Lit. | [322] |
| Loch Clair | GBR | 57.56 | -5.34 | EPD | [327] |
| Loch Cleat | GBR | 57.07 | -6.33 | EPD | [325], [326] |
| Loch Dungeon | GBR | 55.12 | -4.32 | EPD | [286] |
| Loch Einich | GBR | 57.08 | -3.80 | EPD | [286] |
| Loch Fada | GBR | 57.45 | -6.20 | EPD | [315], [326] |
| Loch Laxford | GBR | 58.37 | -5.00 | EPD | [298], [328] |
| Loch Lomond | GBR | 56.10 | -4.61 | Lit. | [329] |
| Loch Lomond Ross Dubh | GBR | 56.09 | -4.58 | EPD | [330] |
| Loch Maree | GBR | 57.08 | -5.48 | EPD | [331] |
| Loch Meodal | GBR | 57.13 | -5.87 | EPD | [325], [326] |
| Loch of Clickimin | GBR | 59.15 | 1.17 | Lit. | [332] |
| Loch Sionascaig | GBR | 58.06 | -5.18 | EPD | [293], [324] |
| Lochan an Druim | GBR | 58.47 | -4.70 | EPD | [333] |
| Lochan coir a' Ghobhainn | GBR | 57.18 | -6.30 | EPD | [315], [326] |
| Lomond Hills | GBR | 56.25 | -2.72 | Lit. | [334] |
| Long Lough | GBR | 54.42 | -5.87 | EPD | [335], [336] |
| Moel y Gerddi | GBR | 52.87 | -4.04 | Lit. | [337] |
| Morrone Birkwoods | GBR | 57.00 | -3.43 | EPD | [338], [339] |
| Norfolk | GBR | 52.50 | -0.83 | Lit. | [310] |
| North Gill, North Yorkshire | GBR | 54.38 | -0.90 | Lit. | [340] |
| Oxford region | GBR | 51.75 | -1.25 | Lit. | [341] |
| Sidlings Copse | GBR | 51.80 | -1.15 | Lit. | [342] |
| Pannel Bridge | GBR | 50.87 | 0.66 | EPD,Lit. | [343], [344] |
| Pickletillem | GBR | 56.40 | -2.90 | EPD | [345] |
| Quoyloo Meadow | GBR | 59.02 | -3.33 | Lit. | [346] |
| Redmere | GBR | 52.43 | 0.43 | EPD | [308] |
| Rough Tor South | GBR | 50.60 | -4.65 | Lit. | [347] |
| Round Loch of Glenhead | GBR | 55.08 | -4.42 | EPD | Stevenson AC (unpublished) |
| Scarborough, Star carr | GBR | 54.22 | -0.47 | Lit. | [348] |
| Slieve Croob | GBR | 54.33 | -5.98 | EPD | [292] |
| Slieve Naslat | GBR | 54.35 | -5.98 | EPD | [292] |
| Stanshiel Rig | GBR | 55.29 | -2.43 | Lit. | [349] |
| Teanga | GBR | 57.32 | -7.28 | EPD | Stevenson AC (unpublished) |
| The Bog [Roos] | GBR | 53.73 | -0.07 | EPD | [350], [351] |
| Thorpe Bulmer | GBR | 54.72 | -1.30 | EPD | [312], [352] |
| Welney Washes | GBR | 52.52 | 0.25 | EPD | [308] |
| Wester Cartmore Farm | GBR | 56.13 | -2.67 | Lit. | [353] |
| Wiggenhall St. Germans | GBR | 52.68 | 0.33 | EPD | [308] |
| Wilden Marsh | GBR | 51.87 | -2.25 | Lit. | [354] |
| Willingham Mere | GBR | 52.33 | -0.05 | EPD | [308] |
| Aghia Galini | GRC | 35.10 | 24.68 | EPD | [355] |
| Delphinos | GRC | 35.33 | 24.28 | Lit. | [356] |
| Edessa | GRC | 40.82 | 21.95 | EPD | [357] |
| Giannitsa B | GRC | 40.67 | 22.32 | EPD | [357] |
| Halos I | GRC | 39.17 | 22.83 | EPD | Bottema S (unpublished) |
| Ioannina I | GRC | 39.76 | 20.73 | EPD | [357] |
| Ioannina lake | GRC | 39.75 | 20.85 | Lit. | [358] |
| Kastoria | GRC | 40.55 | 21.32 | EPD | [357] |
| Khimaditis Ib | GRC | 40.62 | 21.58 | EPD | [357] |
| Khimaditis III | GRC | 40.61 | 21.59 | EPD | [357] |
| Khimaditis III | GRC | 40.61 | 21.59 | EPD | [359] |
| Kopais | GRC | 38.48 | 23.7 | EPD | [360] |
| Kotihi lagoon | GRC | 38.02 | 21.30 | Lit. | [361] |
| Lailias | GRC | 41.27 | 23.60 | EPD | [362] |
| Lake Orestiás | GRC | 40.51 | 21.26 | EPD | [363] |
| Lake Voulkaria | GRC | 38.87 | 20.83 | EPD | [364] |
| Lake Xinias | GRC | 39.05 | 22.27 | EPD | [365] |
| Nisi Fen | GRC | 40.82 | 21.92 | Lit. | [366] |
| Tenaghi Philippon | GRC | 40.98 | 24.78 | EPD | [360] |
| Bokanjacko | HRV | 44.18 | 15.23 | EPD | [367] |
| Lake Vrana, Island of Cres | HRV | 44.85 | 14.39 | Lit. | [368] |
| Mljet island | HRV | 42.78 | 17.35 | Lit. | [369] |
| Alsópáhok | HUN | 46.77 | 17.17 | EPD | [370], [371] |
| Balaton lake, Keszthely-Úsztatómajor | HUN | 46.77 | 17.25 | Lit. | [372] |
| Csögle | HUN | 47.22 | 17.26 | EPD | [370] |
| Nagy-Mohos | HUN | 48.33 | 20.44 | EPD | [370] |
| Pölöske | HUN | 46.76 | 16.92 | EPD | [370], [371], [373] |
| Szigliget | HUN | 46.80 | 17.43 | EPD | [370] |
| Voros-mocsar | HUN | 46.48 | 19.19 | EPD | Nagy-Bodor E, Cserny T, Nagy E (unpublished) |
| Zsombo Swamp | HUN | 46.36 | 19.99 | EPD | [374] |
| Aegelsee | CHE | 46.65 | 7.54 | EPD | [375], [376], [377] |
| Amsoldingersee | CHE | 46.73 | 7.58 | EPD | [378] |
| Bachalpsee | CHE | 46.67 | 8.2 | Lit. | [379] |
| Bibersee | CHE | 47.13 | 8.28 | EPD | [380] |
| Boehnigsee Goldmoos | CHE | 46.26 | 7.84 | EPD | [381] |
| Egelsee | CHE | 47.18 | 8.58 | Lit. | [382] |
| Eggen ob Blatten | CHE | 46.22 | 7.59 | EPD | [11], [12] |
| Etang de la Gruère | CHE | 47.24 | 7.5 | EPD | [383], [384] |
| Etang d'y Cor, Montana | CHE | 46.31 | 7.48 | EPD | [11], [12] |
| Gamperfin | CHE | 47.10 | 9.23 | EPD | [385], [386], [387] |
| Gänsemoos, Schwarzenburg | CHE | 46.50 | 7.21 | EPD | [11], [12], [385], [388] |
| Hopschensee | CHE | 46.15 | 8.1 | EPD | [11], [12] |
| Chutti, Boltigen | CHE | 46.38 | 7.23 | EPD | [11], [389] |
| Lac Superieur de Fully | CHE | 46.18 | 7.9 | EPD | [390] |
| Lac de Fully | CHE | 46.17 | 7.10 | Lit. | [390] |
| Lej da Champfer | CHE | 46.47 | 9.81 | Lit. | [391] |
| Lej da san Murezzan | CHE | 46.49 | 9.85 | Lit. | [391] |
| Linden | CHE | 46.51 | 7.41 | EPD | [12], [385] |
| Lobsigensee | CHE | 47.03 | 7.30 | EPD | [392] |
| Mittlere Hellelen | CHE | 46.17 | 7.50 | EPD | [11], [12] |
| Neugrundmoor | CHE | 47.17 | 8.60 | Lit. | [393] |
| Piano | CHE | 46.32 | 8.62 | Lit. | [394] |
| Praz Rodet | CHE | 46.57 | 6.17 | EPD | [299], [395], [396], [397] |
| Rotsee | CHE | 47.08 | 8.33 | EPD | [377], [398], [399], [400], [401] |
| Soppensee | CHE | 47.09 | 8.8 | Lit. | [402] |
| Wachseldorn Untermoos | CHE | 46.82 | 7.73 | EPD | [12], [385], [400], [403] |
| Wallbach, Lenk | CHE | 46.43 | 7.40 | EPD | [11], [12] |
| Arts Lough | IRL | 52.95 | -6.43 | EPD | [404] |
| Ballinloghig Lake | IRL | 52.20 | -10.31 | EPD | [405] |
| Ballybetagh | IRL | 53.17 | -6.25 | EPD | [406], [407], [408] |
| Belle Lake | IRL | 52.18 | -7.03 | EPD | [409], [410] |
| Coolteen | IRL | 52.35 | -6.60 | EPD | [409], [410] |
| Cregganmore | IRL | 54.25 | -9.60 | EPD | [411] |
| Derrycunihy | IRL | 52.02 | -9.42 | EPD | [412] |
| Newferry | IRL | 54.82 | -6.46 | Lit. | [413] |
| Sluggan Moss | IRL | 54.93 | -6.30 | EPD | [414] |
| Mosfell | ISL | 64.13 | -20.61 | EPD | Hallsdóttir M (unpublished) |
| Azzano Decimo | ITA | 45.89 | 12.72 | Lit. | [415] |
| Balladrum | ITA | 46.02 | 8.75 | Lit. | [416] |
| Biviere di Gela | ITA | 37.02 | 14.33 | Lit. | [417] |
| Ca´Formera | ITA | 45.57 | 12.67 | Lit. | [418] |
| Colfiorito | ITA | 43.03 | 12.93 | EPD | [419] |
| Concordia Sagittaria-Paludetto | ITA | 45.75 | 12.83 | Lit. | [418] |
| Dura-Moor | ITA | 46.64 | 11.46 | EPD | [420] |
| Gorgo Basso | ITA | 37.62 | 12.65 | Lit. | [421] |
| Grünsee, Reschenscheideck | ITA | 46.52 | 10.29 | EPD | [11] |
| Lac de Lod | ITA | 45.80 | 7.84 | EPD | [422] |
| Lac de Villa | ITA | 45.68 | 7.76 | EPD | [422] |
| Lagaccione, Lago di Bolsena | ITA | 42.57 | 11.85 | Lit. | [423] |
| Lago Battaglia, Cargano, coast | ITA | 41.91 | 16.13 | Lit. | [424] |
| Lago del Greppo | ITA | 44.12 | 10.69 | Lit. | [425] |
| Lago del Segrino | ITA | 45.82 | 9.26 | Lit. | [426] |
| Lago dell Costa | ITA | 45.27 | 11.74 | Lit. | [427] |
| Lago dell'Accesa | ITA | 42.99 | 10.88 | EPD,Lit. | [428], [429] |
| Lago di Annone | ITA | 45.80 | 9.35 | Lit. | [430] |
| Lago di Fimon | ITA | 45.47 | 11.53 | Lit. | [431], [432] |
| Lago di Martignano | ITA | 42.12 | 12.33 | EPD | [433] |
| Lago di Muzzano | ITA | 46.00 | 8.93 | Lit. | [426] |
| Lago di Origlio | ITA | 46.06 | 8.94 | Lit. | [434] |
| Lago di Pergusa | ITA | 37.52 | 14.30 | Lit. | [435] |
| Lago di Vico | ITA | 42.32 | 12.17 | Lit. | [436] |
| Lago Grande di Monticchio | ITA | 40.94 | 15.60 | EPD,Lit. | [437], [438] |
| Lago Lucone | ITA | 45.55 | 10.48 | Lit. | [439] |
| Lago Padule | ITA | 44.30 | 10.21 | EPD | [440] |
| Lago Piccolo di Avigliana | ITA | 45.05 | 7.38 | Lit. | [434], [441] |
| Lago Pratignano | ITA | 44.18 | 10.82 | EPD | [440] |
| Malschötscher Hotter | ITA | 46.67 | 11.46 | EPD | [420] |
| Mar Ligure | ITA | 43.80 | 10.32 | Lit. | [442] |
| Ospitale | ITA | 44.16 | 10.78 | EPD | [440] |
| Palughetto mire | ITA | 45.82 | 9.34 | Lit. | [443] |
| Pian di Gembro | ITA | 46.17 | 10.16 | Lit. | [444] |
| Prato Spilla, Appennino Parmense | ITA | 44.36 | 10.11 | Lit. | [445] |
| Ragogna lake | ITA | 46.30 | 13.61 | Lit. | [446] |
| Rifugio Mondovi | ITA | 44.20 | 7.73 | Lit. | [447] |
| Rinderplatz | ITA | 46.64 | 11.49 | EPD | [420] |
| Rome, Tiber delta | ITA | 41.83 | 12.28 | Lit. | [448] |
| Selle di Carnino | ITA | 44.15 | 7.69 | EPD | [240] |
| Schwarzsee | ITA | 46.67 | 11.43 | EPD | [420] |
| Sommersüss | ITA | 46.76 | 11.68 | EPD | [420] |
| Stracciacappa | ITA | 42.13 | 12.32 | Lit. | [449] |
| Tirrenia | ITA | 43.67 | 10.27 | Lit. | [450] |
| Tobiera del Biecai | ITA | 44.19 | 7.72 | Lit. | [447] |
| Tourbière de Champlong | ITA | 45.82 | 7.81 | EPD | [422] |
| Tourbière de Pilaz | ITA | 45.82 | 7.83 | EPD | [422] |
| Tourbière de Santa Anna | ITA | 45.86 | 7.65 | EPD | [422] |
| Bebrukas Lake | LTU | 54.09 | 24.12 | EPD | [451], [452], [453] |
| Biržulis Lake | LTU | 55.78 | 22.43 | Lit. | [454], |
| Pamerkiai outcrop | LTU | 54.31 | 24.73 | Lit. | [455] |
| Svencele Bog | LTU | 55.50 | 21.29 | EPD | Stančikaite M (unpublished) |
| Beaufort Birkenbach | LUX | 49.85 | 6.13 | EPD | [456] |
| Breidfeld | LUX | 50.12 | 6.6 | EPD | [457], [456] |
| Rodenbourg Bretzboesh | LUX | 49.69 | 6.27 | EPD | [456], [457] |
| Lake Kurjanovas | LVA | 56.52 | 27.98 | Lit. | [458] |
| Rudushskoe Lake | LVA | 56.50 | 27.55 | EPD | [459], [460], [461], [462], [463], [464], [465] |
| Lake Ohrid | MKD | 40.90 | 20.63 | Lit. | [466] |
| Bosscherheide | NLD | 51.58 | 6.9 | EPD | [467], [468] |
| Kreekrak | NLD | 51.44 | 4.24 | Lit. | [469] |
| Mariahout | NLD | 51.52 | 5.55 | EPD | [467] |
| Mekelermeer | NLD | 52.77 | 6.62 | EPD | [470], [471] |
| Notsel | NLD | 51.55 | 4.77 | EPD | [472] |
| Zutphen | NLD | 52.15 | 6.15 | Lit. | [469] |
| Andoya-Nedre Erasvatn | NOR | 69.26 | 16.5 | Lit. | [473] |
| Andoya-Ramsa | NOR | 69.18 | 16.9 | Lit. | [473] |
| Blavasstjonn | NOR | 64.92 | 11.67 | EPD | [474], [475] |
| Blomoy | NOR | 60.53 | 4.88 | EPD | [476] |
| Brurskardtjorni | NOR | 61.42 | 8.67 | EPD,Lit. | [477], [478] |
| Bruvatnet | NOR | 70.18 | 28.42 | EPD | [197] |
| Dalane | NOR | 58.25 | 8.00 | Lit. | [479] |
| Dalmutladdo | NOR | 69.17 | 20.72 | EPD | [480] |
| Domsvatnet | NOR | 70.32 | 31.3 | EPD | [481] |
| Donvold | NOR | 68.13 | 13.58 | EPD | Nilssen EJ (unpublished) |
| Fitjar | NOR | 59.93 | 5.33 | Lit. | [482] |
| Flaatevatn | NOR | 59.70 | 6.17 | EPD | [483] |
| Fläfattjonna | NOR | 62.33 | 10.33 | Lit. | [484] |
| Frengstadsetra | NOR | 62.57 | 10.13 | EPD | [485] |
| Gauptjern | NOR | 68.86 | 19.62 | Lit. | [486] |
| Gorrmyra | NOR | 68.86 | 19.58 | Lit. | [486] |
| Grasvatn | NOR | 63.70 | 8.70 | EPD | [487] |
| Grostjørna | NOR | 58.53 | 7.73 | Lit. | [479] |
| Holebudalen | NOR | 59.83 | 7.00 | Lit. | [479] |
| Jervtjern | NOR | 68.86 | 19.59 | Lit. | [486] |
| Lake Fläfattjonna | NOR | 62.33 | 10.40 | Lit. | [488] |
| Lake Hopseidet | NOR | 70.83 | 27.72 | EPD | [210] |
| Lake Ifjord | NOR | 70.43 | 27.63 | EPD | [210] |
| Leirdalen | NOR | 61.56 | 8.37 | Lit. | [489] |
| Lille Kjelavatn | NOR | 58.83 | 7.00 | Lit. | [479] |
| Melkøya, Kilden | NOR | 70.70 | 23.60 | Lit. | [490] |
| Melkøya, Sunden | NOR | 70.70 | 23.60 | Lit. | [490] |
| Rattuvarri | NOR | 69.35 | 20.32 | EPD | [217] |
| Sandvikvatn | NOR | 59.28 | 5.50 | EPD | Paus AA (unpublished) |
| Semmeldalen | NOR | 76.67 | 15.33 | EPD | [491] |
| Skrubbtjern | NOR | 68.89 | 19.61 | Lit. | [486] |
| Sørøya | NOR | 70.53 | 22.90 | Lit. | [492] |
| Svanavatnet | NOR | 66.42 | 14.5 | Lit. | [493] |
| Trettetjorn | NOR | 60.72 | 7.00 | EPD,Lit. | [477], [478], [494] |
| Trollvatnet | NOR | 69.88 | 23.47 | EPD | [495] |
| Vestre Oykjamyrtorn | NOR | 59.82 | 6.00 | EPD | [477], [478], [494] |
| Bledowo Lake | POL | 52.55 | 20.67 | EPD | [496], [497] |
| Cergowa Gora | POL | 49.53 | 21.70 | EPD | [498], [499], [500] |
| Czajkow | POL | 50.78 | 21.28 | EPD | [501] |
| Darzlubie Forest | POL | 54.70 | 18.17 | EPD | [502],[503], [504] |
| Giecz | POL | 52.32 | 17.36 | EPD | [505] |
| Great Mazurian Lake | POL | 53.85 | 21.83 | Lit. | [506] |
| Jasiel | POL | 49.37 | 21.89 | EPD | [507], [508] |
| Jaslo | POL | 49.78 | 21.47 | EPD | [509] |
| Jezioro Druzno | POL | 54.12 | 19.47 | EPD | [510], [511] |
| Kluki | POL | 54.71 | 17.28 | EPD | [512] |
| Lake Hancza | POL | 54.27 | 22.82 | EPD | [513] |
| Lake Lednica | POL | 52.56 | 17.39 | EPD | [514] |
| Lake Mikolajki | POL | 53.77 | 21.42 | EPD | [515], [516] |
| Lake Racze | POL | 53.92 | 14.67 | EPD | [517], [518], [519], [520], [521], [522] |
| Lake Skrzetuszewskie | POL | 52.55 | 17.36 | EPD | [523], [524] |
| Maly Suszek | POL | 53.73 | 17.77 | EPD | [525] |
| Niechorze | POL | 54.00 | 15.5 | EPD | [526] |
| Puscizna Rekowianska | POL | 49.48 | 19.82 | EPD | [527], [528], [529], [530] |
| Roztoki | POL | 49.72 | 21.58 | EPD | [531] |
| Rudnickie Male | POL | 53.43 | 18.75 | EPD | [532] |
| Slawsko | POL | 52.67 | 18.25 | EPD | Milecka M (unpublished) |
| Slopiec | POL | 50.78 | 20.78 | EPD | [533], [534], [535], [536] |
| Szymbark | POL | 49.63 | 21.10 | EPD | [508], [537], [538] |
| Tarnawa Wyzna | POL | 49.10 | 22.83 | EPD | Ralska-Jasiewiczowa M (unpublished) |
| Tarnowiec | POL | 49.70 | 21.62 | EPD | [531] |
| Volin island | POL | 54.00 | 14.63 | Lit. | [539] |
| Wolbrom | POL | 50.38 | 19.77 | EPD | [540], [541], [542] |
| Wolin II | POL | 53.83 | 14.67 | EPD | [504], [519], [520], [521], [522] |
| Woryty | POL | 53.75 | 20.20 | EPD | [543], [544], [545] |
| Zarnowiec Peat Bog | POL | 54.72 | 18.12 | EPD | [502], [503], [504] |
| Covao do Boieiro | PRT | 40.33 | -6.38 | Lit. | [546] |
| Charco da Candieira | PRT | 40.34 | -6.42 | Lit. | [546], [547] |
| Charco da Candieira | PRT | 40.34 | -7.58 | EPD | [546], [547], [548] |
| Charco dos Coes | PRT | 40.34 | -6.40 | Lit. | [546] |
| Lagoa Clareza | PRT | 40.34 | -6.40 | Lit. | [546] |
| Lagoa Comprida | PRT | 40.36 | -6.36 | Lit. | [546] |
| Lagoa Comprida 2 | PRT | 40.36 | -7.64 | EPD | [549], [550], [551] |
| Lagoa das Salgadeiras | PRT | 40.34 | -6.39 | Lit. | [546] |
| Lower Tagus Basin | PRT | 39.03 | -7.09 | Lit. | [552] |
| Apuseni-IC Ponor | ROU | 45.86 | 22.52 | Lit. | [553] |
| Avrig | ROU | 45.72 | 24.38 | Lit.,EPD | [554], [555], [556] |
| Bergerie | ROU | 45.86 | 22.52 | Lit. | [553] |
| Bisoca peat bogs | ROU | 45.53 | 26.82 | Lit. | [557] |
| Cimetiere | ROU | 45.86 | 22.52 | Lit. | [553] |
| Iezerul Caliman | ROU | 47.33 | 25.27 | Lit. | [558] |
| Luci | ROU | 46.27 | 25.75 | Lit. | [556] |
| Mohos | ROU | 46.08 | 25.92 | EPD | [554], [559] |
| Preluca Tiganului | ROU | 47.82 | 23.54 | Lit.,EPD | [560], [561], [562], [563], [564] |
| Semenic | ROU | 45.18 | 22.6 | Lit.,EPD | [135], [565] |
| Steregoiu | ROU | 47.81 | 23.54 | Lit.,EPD | [560], [564], [566], [567] |
| Taul Zanoguti | ROU | 45.33 | 22.80 | Lit. | [558] |
| Turbuta | ROU | 47.37 | 23.50 | Lit. | [568] |
| Babozero | RUS | 66.38 | 37.52 | EPD | [569] |
| Bezdonnoe | RUS | 62.03 | 32.77 | EPD | [570], [571] |
| Cape Shpindler | RUS | 69.72 | 62.80 | Lit. | [572] |
| Chernaya Gorka | RUS | 67.08 | 65.35 | Lit. | [573] |
| Chistic | RUS | 57.34 | 33.00 | EPD | [574] |
| Chuna lake | RUS | 67.95 | 32.48 | Lit. | [575] |
| Dlinnoe | RUS | 62.32 | 33.85 | EPD | [576] |
| Glubokoe | RUS | 61.07 | 36.05 | EPD | [570] |
| Gotnavolok | RUS | 62.20 | 33.80 | EPD | [570], [571], [577], [578] |
| Ivanovskoye Peat Bog | RUS | 56.82 | 38.77 | EPD | Spiridonova EA (unpublished) |
| Kepskoe | RUS | 65.08 | 32.17 | EPD | [570] |
| Krugloye | RUS | 66.37 | 37.58 | EPD | [569] |
| Kunyok | RUS | 67.83 | 33.67 | EPD | [579] |
| Lake KP3 | RUS | 69.07 | 36.01 | Lit. | [580] |
| Lake Lemolovskoye | RUS | 60.36 | 30.32 | Lit. | [581] |
| Lake Nero | RUS | 57.18 | 39.45 | EPD | [582], [583] |
| Lake Pieni-Kuuppalanlampi | RUS | 61.28 | 29.92 | Lit. | [584] |
| Lake Tambichozero | RUS | 61.93 | 37.90 | Lit. | [585] |
| Landshaftnoe | RUS | 64.57 | 30.53 | EPD | [570] |
| Lutnermayok peat bog | RUS | 67.68 | 33.28 | Lit. | [586] |
| Mezhgornoe | RUS | 66.37 | 30.70 | EPD | [570] |
| Mire Petrolivo | RUS | 56.00 | 31.98 | EPD | [587] |
| Mire Sosvyatskoe | RUS | 56.20 | 32.00 | EPD | [587] |
| Mshinskoye | RUS | 59.87 | 29.92 | Lit. | [581] |
| Murman cost | RUS | 69.07 | 36.01 | Lit. | [588] |
| Mustusuo | RUS | 61.81 | 33.50 | EPD | [570] |
| Nemino | RUS | 62.75 | 34.58 | EPD | [570] |
| Nenazvannoe | RUS | 61.81 | 33.48 | EPD | [570] |
| Neor Lake | RUS | 57.17 | 39.43 | Lit. | [589] |
| Nosuo | RUS | 64.57 | 30.83 | EPD | [570] |
| Poeryanny Zub Lake | RUS | 68.83 | 35.33 | Lit. | [590] |
| Priventninskoye | RUS | 60.18 | 29.44 | Lit. | [591] |
| Ptichje | RUS | 66.35 | 30.57 | EPD | [570] |
| Rugozero | RUS | 64.08 | 32.63 | EPD | [570] |
| Rybachiy | RUS | 69.63 | 32.37 | EPD | [579] |
| Shombashuo | RUS | 65.12 | 32.63 | EPD | [570] |
| Solnechnoe | RUS | 65.83 | 34.33 | EPD | [570] |
| Stupino | RUS | 52.25 | 39.83 | EPD | [592], [593] |
| Tchernaya Rechka | RUS | 60.19 | 29.54 | Lit. | [591] |
| Timan Ridge | RUS | 67.27 | 48.72 | Lit. | [594] |
| Valdai Hills-Staroselsky Moch | RUS | 56.58 | 32.92 | Lit. | [595] |
| Vishnevskoe Lake | RUS | 60.50 | 29.52 | EPD | [464], [465], [596], [597], [598] |
| Vysokinskoye | RUS | 60.31 | 28.87 | Lit. | [591] |
| Zapovednoe | RUS | 65.12 | 32.63 | EPD | [570] |
| Zaruckoe | RUS | 63.90 | 36.25 | EPD | [570] |
| Gorenje jezero | SLO | 45.73 | 14.31 | Lit. | [599] |
| Griblje marsh | SLO | 45.57 | 15.28 | Lit. | [600] |
| Lake Bled | SLO | 46.37 | 14.10 | Lit. | [601] |
| Ljubljansko barje | SLO | 45.98 | 14.54 | Lit. | [602] |
| Ljubljansko barje/Na mahu | SLO | 45.98 | 14.54 | Lit. | [602] |
| Mlaka | SLO | 45.50 | 15.21 | Lit. | [600] |
| Prapoče | SLO | 45.43 | 14.18 | Lit. | [599] |
| Bobrov | SVK | 49.45 | 19.57 | EPD | [603] |
| Hozelec | SVK | 49.05 | 18.30 | EPD | [604] |
| Liptovsky Jan | SVK | 49.04 | 19.68 | EPD | Rybníček K (unpublished) |
| Popradské pleso | SVK | 39.08 | 20.7 | Lit. | [605] |
| Regetovka | SVK | 49.43 | 21.28 | EPD | [606] |
| Šafárka | SVK | 48.88 | 20.58 | Lit. | [80] |
| Zlatnicka Dolina | SVK | 49.52 | 19.28 | EPD | [603] |
| Ageröds Mosse | SWE | 55.83 | 13.42 | EPD | [607] |
| Åkerhultagöl [Tomtabaken] | SWE | 57.48 | 14.47 | EPD | [608], [609] |
| Åsbotorpsjön | SWE | 58.42 | 13.83 | EPD | Digerfeld G (unpublished) |
| Bergakyllen | SWE | 57.17 | 16.15 | EPD | [610] |
| Bjorksjodamm | SWE | 57.71 | 12.39 | EPD | [609], [611] |
| Blekinge, Kalvöviken | SWE | 56.17 | 15.12 | Lit. | [612] |
| Blekinge, Sörevik | SWE | 56.12 | 15.77 | Lit. | [612] |
| Domsjön | SWE | 58.30 | 12.45 | EPD | [609], [613], [614], [615] |
| Dumpokjaruatl | SWE | 66.07 | 18.37 | Lit. | [616] |
| Färskesjön | SWE | 56.17 | 15.87 | Lit. | [617] |
| Fjällnas | SWE | 62.55 | 12.17 | EPD | [618], [619] |
| Fjallsjön 1 | SWE | 57.75 | 12.86 | EPD | [609], [611] |
| Galtsjön | SWE | 56.22 | 15.22 | EPD | [609], [620] |
| Hälsegyl | SWE | 56.55 | 14.61 | EPD | [621] |
| Högstorpsmossen | SWE | 57.95 | 15.68 | Lit. | [622], [623] |
| Hunnemara | SWE | 56.17 | 14.88 | Lit. | [624] |
| Ipmatisjauratj | SWE | 66.17 | 18.17 | Lit. | [616] |
| Kalven | SWE | 56.53 | 14.56 | EPD | [621] |
| Kansjon | SWE | 57.63 | 14.53 | EPD | Jacobson GL (unpublished) |
| Krageholmssjön | SWE | 55.50 | 13.75 | Lit. | [617] |
| Lake Badsjön | SWE | 68.33 | 18.75 | Lit. | [625] |
| Lake Flarken | SWE | 58.58 | 13.67 | EPD | [626] |
| Lake Lattok | SWE | 65.96 | 18.35 | Lit. | [627] |
| Lake Ryssjön | SWE | 56.17 | 15.8 | Lit. | [628] |
| Lake Raigejegge | SWE | 66.15 | 18.21 | Lit. | [627] |
| Lake Sambösjön | SWE | 57.13 | 12.42 | EPD | [629] |
| Lake Vibysjön | SWE | 59.03 | 14.88 | Lit. | [630] |
| Lillsjön | SWE | 57.08 | 12.53 | EPD | Björck S (unpublished) |
| Ljungsjön | SWE | 57.73 | 13.33 | EPD | [609], [611] |
| Logylet | SWE | 56.30 | 14.98 | EPD | [609], [620] |
| Mabo Moss | SWE | 58.02 | 16.7 | EPD | [631] |
| Mullsjön | SWE | 58.28 | 14.23 | EPD | [632] |
| Närke-Ladfallet | SWE | 58.83 | 15.92 | Lit. | [633] |
| Närke-Majorsmossen | SWE | 58.83 | 15.92 | Lit. | [633] |
| Närke-Östra Torrtallemossen | SWE | 58.83 | 15.92 | Lit. | [633] |
| Närke-Skelnabäckakärret | SWE | 58.83 | 15.92 | Lit. | [633] |
| Nedre Madbergagölen | SWE | 58.60 | 12.17 | EPD | [634] |
| Skvarran | SWE | 57.20 | 16.15 | EPD | [610] |
| Smygen | SWE | 56.15 | 15.12 | Lit. | [624] |
| Spaime Lake | SWE | 63.12 | 12.32 | Lit. | [635] |
| Spjällsjön | SWE | 56.69 | 14.59 | EPD | [621] |
| Stavsåkra | SWE | 56.92 | 14.90 | Lit. | [682] |
| Stora Gilltjärnen | SWE | 60.08 | 15.83 | Lit. | [636] |
| Svartkälstjärn | SWE | 64.27 | 19.55 | Lit. | [637] |
| Trehörningen | SWE | 58.55 | 11.60 | Lit. | [638] |
| Vingölen | SWE | 57.13 | 15.95 | EPD | [610] |
| Vuolep Njakajaure | SWE | 68.33 | 18.78 | Lit. | [639] |
| Akgöl Adabag | TUR | 37.50 | 33.73 | EPD | [640] |
| Beysehir Gölü I | TUR | 37.54 | 31.50 | EPD | Bottema S (unpublished) |
| Kararmik Batakligi | TUR | 38.43 | 30.80 | EPD | [641] |
| Köycegiz Gölü | TUR | 36.88 | 28.64 | EPD | Bottema S (unpublished) |
| Ova Gölü | TUR | 36.27 | 29.30 | EPD | Bottema S (unpublished) |
| Sögüt Gölü | TUR | 37.00 | 29.90 | EPD | [641] |
| Dovjok Swamp | UKR | 48.75 | 28.25 | EPD | [642] |
| Gel'myazevskoye | UKR | 49.67 | 31.83 | EPD | [643], [644], [645] |
| Iosipovo | UKR | 51.20 | 28.00 | EPD | [646], [647], [648], [649] |
| Kardashinski Swamp | UKR | 46.52 | 32.62 | EPD | [642] |
| Khomin Mokh | UKR | 51.20 | 28.00 | EPD | [646], [647], [648], [649] |
| Miroshy | UKR | 51.20 | 28.00 | EPD | [646], [647], [648], [649] |
| Starniki | UKR | 50.27 | 26.2 | EPD | [645], [650], [651], [652], [653], [654], [655] |
| Stoyanov 2 | UKR | 50.38 | 24.63 | EPD | [643], [644], [645], [652], [654], [656], [657] |
| Turova Dacha | UKR | 48.90 | 24.10 | EPD | [646], [647], [648], [649] |
| Vološča | UKR | 49.55 | 23.83 | Lit. | [658] |
| Zalozhtsy | UKR | 49.75 | 25.45 | EPD | [643], [644], [645], [650], [652] |
