# Supplementary material for: Migration Patterns of Subgenus Alnus in Europe since the Last Glacial Maximum: A Systematic Review
Source: PLoS One. 2014 Feb 21;9(2):e88709. doi: 10.1371/journal.pone.0088709 (PMC3931649; doi:10.1371/journal.pone.0088709)
Supplement: Table S2 — Location of the macrofossil sites from NEMD and the literature (Lit.). (DOCX) [file pone.0088709.s004.docx]

**Table S2. Location of the macrofossil sites from NEMD and the literature (Lit.).**

| ***Site*** | ***Country*** | ***Longitude*** | ***Latitude*** | ***Source*** | ***Reference*** |
| --- | --- | --- | --- | --- | --- |
| Ezero wetland, Thracia plain | BLG | 42.47 | 26.02 | Lit. | [30] |
| Pirin | BLG | 41.45 | 23.43 | Lit. | [660] |
| Gozha | BLR | 53.8 | 23.85 | NEMD | [661] |
| Plaskovtsy | BLR | 53.56 | 25.16 | NEMD | [662] |
| Bohunice | CZE | 49.17 | 16.58 | Lit. | [663] |
| Švarcenberk | CZE | 49.15 | 14.7 | Lit. | [96] |
| Jues Lake, Harz | DEU | 51.66 | 10.35 | Lit. | [123] |
| Steisslingen lake | DEU | 47.8 | 8.9 | Lit. | [136] |
| Solova Bog | EST | 57.7 | 27.42 | Lit. | [664] |
| Vyallamyagi | EST | 57.75 | 27.16 | NEMD | [665] |
| Navia | FRA | 43.48 | 5.43 | Lit. | [666] |
| Villaviciosa | FRA | 43.52 | 6.72 | Lit. | [666] |
| Brede Bridge, East Sussex | GBR | 50.93 | 0.71 | Lit. | [308] |
| Brookland | GBR | 51.05 | -0.8 | Lit. | [311] |
| Hope Farm | GBR | 51.05 | -0.8 | Lit. | [311] |
| Horsemarsh Sewer | GBR | 51.05 | -0.8 | Lit. | [311] |
| Isle Harris | GBR | 58 | -6.79 | Lit. | [322] |
| Kinloch, Isle of Rhum | GBR | 58.27 | -4.82 | Lit. | [313] |
| Pannel Bridge | GBR | 50.87 | 0.66 | Lit. | [344] |
| Scarborough, Star carr | GBR | 54.22 | -0.47 | Lit. | [348] |
| The Dowels | GBR | 51.05 | -0.8 | Lit. | [311] |
| Wales, Barlands Farm | GBR | 51.57 | -2.83 | Lit. | [667] |
| Wales, Vurlong Reen I | GBR | 51.58 | -2.8 | Lit. | [667] |
| Walton Moss, Cumbria | GBR | 55 | -2.79 | Lit. | [668] |
| Willow Garth | GBR | 54.09 | -0.28 | Lit. | [669] |
| Lej da san Murezzan | CHE | 46.49 | 9.85 | Lit. | [670] |
| Soppensee | CHE | 47.09 | 8.08 | Lit. | [402] |
| Lago Grande di Monticchio | ITA | 40.93 | 15.6 | Lit. | [438] |
| Lago Piccolo di Avigliana | ITA | 45.05 | 7.38 | Lit. | [434] |
| Osechenskoe | LAT | 57.53 | 24.86 | NEMD | [671] |
| Bebrukas | LTU | 54.58 | 24.61 | NEMD | [672] |
| Lake Kašučiai | LTU | 55.99 | 21.31 | Lit. | [455] |
| Rudnya | LTU | 54.06 | 24.66 | NEMD | [662], [673] |
| Kreekrak | NL | 51.44 | 4.24 | Lit. | [469] |
| Brurskardtjorni | NOR | 61.42 | 8.67 | Lit. | [478] |
| Dalane | NOR | 58.25 | 8 | Lit. | [479] |
| Dalmutladdo | NOR | 69.17 | 20.72 | Lit. | [480] |
| Gauptjern | NOR | 68.86 | 19.62 | Lit. | [486] |
| Gorrmyra | NOR | 68.86 | 19.58 | Lit. | [486] |
| Grostjørna | NOR | 58.53 | 7.73 | Lit. | [479] |
| Jervtjern | NOR | 68.86 | 19.59 | Lit. | [486] |
| Mt. Storsnasen | NOR | 63.47 | 11.84 | Lit. | [674] |
| Trettejorn | NOR | 60.72 | 7 | Lit. | [478] |
| Great Mazurian Lake | POL | 53.85 | 21.83 | Lit. | [506] |
| Volin island | POL | 54 | 14.63 | Lit. | [539] |
| Mitoc Malu Galben | ROM | 48.12 | 27.03 | Lit. | [663] |
| Don | RUS | 53.65 | 38.79 | NEMD | Novenko EY (unpublished) |
| Chashnitsy | RUS | 56.94 | 39.38 | NEMD | [589] |
| Kovash | RUS | 59.9 | 29.11 | NEMD | [675], [676] |
| Lake Chashnitsy | RUS | 56.94 | 39.38 | Lit. | [589] |
| Lutnermayok peat bog | RUS | 67.68 | 33.28 | Lit. | [586] |
| Megrega | RUS | 61.93 | 33.1 | NEMD | [677] |
| Shenskoe | RUS | 58.51 | 37.11 | NEMD | [678] |
| Rybachiy peat bog | RUS | 69.63 | 32.37 | Lit. | [579] |
| Ugra | RUS | 54.67 | 34.1 | NEMD | Novenko EY (unpublished) |
| Usinsk mire | RUS | 65.75 | 57.5 | NEMD | [679] |
| Viun | RUS | 60.55 | 30.58 | NEMD | [677] |
| Volha river, Pobochnoye swamp | RUS | 53.03 | 51.84 | Lit. | [680] |
| Blekinge, Kalvöviken | SWE | 56.17 | 15.12 | Lit. | [612] |
| Blekinge, Sörevik | SWE | 56.12 | 15.77 | Lit. | [612] |
| Hunnemara | SWE | 56.17 | 14.88 | Lit. | [624] |
| Lake Badsjön | SWE | 68.33 | 18.75 | Lit. | [625] |
| Lake Latteluokta | SWE | 68.25 | 19.98 | Lit. | [625] |
| Lake Ryssjön | SWE | 56.17 | 15.08 | Lit. | [628] |
| Mt. Getryggen | SWE | 63.17 | 12.37 | Lit. | [681] |
| Smygen | SWE | 56.15 | 15.12 | Lit. | [624] |
| Spaime Lake | SWE | 63.12 | 12.32 | Lit. | [635] |
| Stavsåkra | SWE | 56.92 | 14.9 | Lit. | [682] |
