# Supplementary material for: Migration Patterns of Subgenus Alnus in Europe since the Last Glacial Maximum: A Systematic Review
Source: PLoS One. 2014 Feb 21;9(2):e88709. doi: 10.1371/journal.pone.0088709 (PMC3931649; doi:10.1371/journal.pone.0088709)
Supplement: Table S3 — References of the pollen and macrofossil sites from EPD, PALYCZ, NEMD and the literature (Lit.). (DOCX) [file pone.0088709.s005.docx]

**Table S3. References of the pollen and macrofossil sites from EPD, PALYCZ, NEMD and the literature (Lit.).**

| 1. Denèfle M, Lézine A-M, Fouache E, Dufaure J-J (2000) A 12,000-Year Pollen Record from Lake Maliq, Albania. Quaternary Res 54: 423–432. |
| --- |
| 1. Bortenschlager S (1984) Beiträge zur Vegetationsgeschichte Tirols I. Inneres Ötztal und unteres Inntal. Berichte des naturwissenschaftlich-medizinischen Vereins in Innsbruck 71: 19–56. |
| 1. Weirich J, Bortenschlager S (1980) Beiträge zur Vegetationsgeschichte Tirols III: Stubaier Alpen - Zillertaler Alpen. Berichte des naturwissenschaftlich-medizinischen Vereins in Innsbruck 67: 7–30. |
| 1. Heuberger H (1977) Gletscher- und klimageschichtliche Untersuchungen im Zemmgrund. Alpenvereinsjahrbuch 102: 39–50. |
| 1. Hüttemann H, Bortenschlager S (1987) Beiträge zur Vegetationsgeschichte Tirols VI: Riesengebirge, Hohe Tatra-Zillertal. Kühtai. Berichte des naturwissenschaftlichen-medizinischen Verein Innsbruck 74: 81–112. |
| 1. Wahlmüller N (1985) Beiträge zur Vegetationsgeschichte Tirols V: Nordtiroler Kalkalpen. Berichte des naturwissenschaftlich-medizinischen Vereins in Innsbruck 72: 101–144. |
| 1. Voigt R (1996) Paläolomnolische und vegetationsgeschichtliche Untersuchungen an Sedimenten aus Fuschlsee und Chiemsee (Salzburg und Bayern). Diss Bot 270: 1–303. |
| 1. Bortenschlager I (1976) Beiträge zur Vegetationsgeschichte Tirols II: Kufstein - Kitzbühel - Pass Thurn. Berichte des naturwissenschaftlich-medizinischen Vereins in Innsbruck 63: 105–137. |
| 1. Krisai R, Mayer W, Schröck C, Türk R (2006) Das Gradenmoos in der Schobergruppe (NP Hohe Tauern, Kärten): Vegetation und Entstehung. Carinthia II 196/116: 359–386. |
| 1. Bortenschlager S (1993) Das höchst gelegene Moor der Ostalpen Moor am Rofenberg 2760 m. Festschrift Zoller. Diss Bot 196: 329–334. |
| 1. Welten M (1982a) Pollenanalytische Untersuchungen zur Vegetationsgeschichte des Schweizerischen Nationalparks. Ergebnisse der wissenschaftlichen Untersuchungen im Schweizerischen Nationalpark XVI/80: 1–43. |
| 1. van der Knaap WO, Ammann B (1997) Depth-age relationships of 25 well-dated Swiss Holocene pollen sequences archived in the Alpine Palynological Data-Base. Revue de Paléobiologie 16: 433–480. |
| 1. Oeggl K (1988) Beiträge zur Vegetationgeschichte Tirols VII: Das Hochmoor Schwemm bei Wachsee. Berichte des naturwissenschaftlich-medizinischen Vereins in Innsbruck 75: 37–60. |
| 1. Krisai R (2006) Mooruntersuchungen im Stubachtal (Hohe Tauern, Salzburg). Beiträge zur Naturkunde Oberösterreichs 16: 105–147. |
| 1. Krisai R (1975) Die Ufervegetation der Trumer Seen (Salzburg). Diss Bot 29: 1–202. |
| 1. Rybníček K, Rybníčková E (1977) Mooruntersuchung im ob oberen. Folia Geobot 12: 245–291. |
| 1. Gilot E, Munaut A-V, Couteaux M, Heim J, Capron P, et al. (1969) Datations 14C et palynologie en Belgique et dans les régions voisines. Bulletin de la Société Belge de Géologie, Paleontologie, Hydrologie 78: 21–29. |
| 1. Zernitskaya V, Mikhailov N (2009) Evidence of early farming in the Holocene pollen spectra of Belarus. Quatern Int 203: 91–104. |
| 1. Verbruggen C (1979a) Paleoecologische en palynlogische benadering van enkele bekende historisch-geografisch problemen in Vlaaderen. Bronnen voor de historisch geografie van Belgie. Handelingen van het Colloquium te Brussel 25–27–IV: 487–497. |
| 1. Verbruggen C, Denys L, Kiden P (1996) Palaeoecological events in Belgium during the last 13,000 years with special reference to sandy Flanders. In: Berglund BE, Birks HJB, Ralska–Jasiewiczowa M, Wright HE, editors. Palaeoecological Events During the Last 15000 Years. Chichester, England: Wiley. pp. 553–574. |
| 1. Verbruggen C (1979b) Vegetational and palaeoecological history of the Lateglacial period in Sandy Flanders (Belgium). Acta Universita Oulu, Oulu, Finland 3: 133–142. |
| 1. Denys L, Verbruggen C, Kiden P (1990) Palaeolimnological aspects of a Late-Glacial shallow lake in Sandy Flanders, Belgium. Hydrobiologia 214: 273–278. |
| 1. Bozilova E, Beug HJ (1992) On the Holocene history of vegetation in SE Bulgaria (Lake Arkutino, Ropotamo region). Veg Hist Archaeobot 1: 19–32. |
| 1. Lazarova MA, Tonkov S, Marinova E, Ivanov D, Bozilova EDB (2011) Contributions to the European Pollen Database. 12. Western Rhodopes Mountains (Bulgaria): peat bog Beliya Kanton. Grana 50: 162–164. |
| 1. Stefanova I, Bozilova E (1995) Studies on the Holocene history of vegetation in the Northern Pirin Mountains, southwestern Bulgaria. In: Bozilova E, Tonkov S, editors. Advances in Holocene Palaeoecology in Bulgaria. Sofia–Moscow: Pensoft Publication. pp. 9–31. |
| 1. Stefanova I, Atanassova J, Delcheva M, Wright H (2006) Chronological framework for the Lateglacial pollen and macrofossil sequence in the Pirin Mountains, Bulgaria: Lake Besbog and Lake Kremensko-5. Holocene 16: 877–892. |
| 1. Shopov VS, Bozilova E, Atanassova J (1992) Biostratigraphy and radiocarbon data of upper quaternary sediments from the western part of the Black Sea. Geologica Balcanica 22: 59–70. |
| 1. Bozilova E, Smith AG (1979) Palynology of lake Sucho Ezero from South Rila Mountain (Bulgaria). Phytologia 11: 54–67. |
| 1. Bozilova E, Tonkov S, Pavlova D (1986) Pollen and plant macrofossil analyses of the Lake Sucho Ezero in the south Rila mountains. Annual of Sofia University, Faculty of Biology 80: 48–57. |
| 1. Magyari E, Chapman J, Gaydarska B, Marinova E, Deli T, et al. (2008) The ‘oriental’component of the Balkan flora: evidence of presence on the Thracian Plain during the Weichselian late-glacial. J Biogeogr 35: 865–883. |
| 1. Huttunen A, Huttunen R, Vasari Y, Panovska H, Bozilova E (1992) Late Glacial and Holocene history of flora and vegetation in the Western Rhodopes Mountains Bulgaria. Acta Botanica Fennica 144: 63–80. |
| 1. Stefanova I, Ammann B (2003) Lateglacial and Holocene vegetation belts in the Pirin Mountains (southwestern Bulgaria). Holocene 13: 97–107. |
| 1. Bozilova E, Tonkov S (1985a) Palaeoecological studies in lake Durankulak. Annual of Sofia University, Faculty of Biology 76: 25–29. |
| 1. Bozilova E, Filipova M (1986) Paleoecological environment in northeastern Black Sea area during Neolithic, Eneolithic and Bronze periods. Studia Praehistoria 8: 160–165. |
| 1. Tonkov S, Panovska H, Possnert G, Bozilova E (2002) The Holocene vegetation history of Northern Pirin Mountain, southwestern Bulgaria: pollen analysis and radiocarbon dating of a core from Lake Ribno Ban derishko. Holocene 12: 201–210. |
| 1. Bozilova E, Tonkov S (2000) Pollen from Lake Sedmo Rilsko reveals southeast European postglacial vegetation in the highest mountain area of the Balkans. New Phytol 148: 315–325. |
| 1. Filipova M (1985) Plaeoecological investigations of lake Shabla-Ezeretz in NE Bulgaria. Ecol Mediterr 11: 148–158. |
| 1. Lazarova MA (1995) Human impact on the natural vegetation in the region of Lake Srebarna and mire Garvan (northeastern Bulgaria). Palynological and Palaeoethnobotanical evidence. In: Bozilova E, Tonkov S, editors. Advances in Holocene Palaeoecology in Bulgaria. Sofia–Moscow: Pensoft Publication. pp. 47–67. |
| 1. Bozilova E, Tonkov S (1985b) Vegetational development in the mountainous areas of SW Bulgaria. I. Palynological investigation and reconstruction of past vegetation. Ecol Mediterr 9: 33–37. |
| 1. Tonkov S, Bozilova E (1992a) Paleoecological investigation of Tchokljovo Marsh (Konjavska Mountains). Annual of Sofia University, Faculty of Biology 83: 5–16. |
| 1. Tonkov S (2003) Holocene palaeovegetation of the Northwestern Pirin Mountains (Bulgaria) as reconstructed from pollen analysis. Rev Palaeobot Palynol 124: 51–61. |
| 1. Panovska H, Bozilova E, Tonkov S (1990) Late Holocene vegetational history in the western part of Belasitza Mt. In: Baltackov G, editor. Geographica Rhodopica 2. Thessaloniki: Aristote Univ. Press. pp. 1–7. |
| 1. Panovska H, Bozilova E, Tonkov S (1995) A palaeoecological investigation on the vegetation history in the Southern Pirin Mountains (southwestern Bulgaria). In: Bozilova E, Tonkov S, editors. Advances in Holocene Palaeoecology in Bulgaria. Sofia–Moscow: Pensoft Publication. pp. 32–46. |
| 1. Atanassova J, Stefanova I (2003) Late-glacial vegetation history of Lake Kremensko-5 in the northern Pirin Mountains, southwestern Bulgaria. Veg Hist Archaeobot 12: 1–6. |
| 1. Filipovitch L (1977) Palynological data for the postglacial distribution of Juglans in the composition of Bulgarian flora. Phytology 6: 32–37. |
| 1. Petrov SI, Filipovitch L (1987) Postglacial changes of the vegetation on the slopes of Sredna Gora mountain. In: Kuzmanov B, editor. Proceedings of Fourth National Botanical Conference. Sofia: Bulgarian Academy of Sciences. pp. 339–406. |
| 1. Filipovitch L (1992) Anthropogenic activity as a factor for the formation of contemporary plant communities in the central Sredna Gora mountain. Fitologija 43: 30–35. |
| 1. Tonkov S, Possnert G, Bozilova E (2006) The lateglacial vegetation and radiocarbon dating of Lake Trilistnika, Rila Mountains (Bulgaria). Veg Hist Archaeobot 16: 15–22. |
| 1. Tonkov S, Bozilova E (1992b) Pollen analysis of peat bog in Maleshevska mountain (SW Bulgaria). Annual of Sofia University, Faculty of Biology 81: 11–21. |
| 1. Chakalova E, Stojanova D, Tonkov S (1990) Plant macrofossil remains from the Chokljovo Marsh (Konjavska Mountains). Annual of Sofia University, Faculty of Biology 80: 41–47. |
| 1. Filipovitch L (1985) Palynological studies of peat bogs on the southern slopes of Vitosha mountain. Forest Sci 2: 3–16. |
| 1. Filipovitch L (1988) Vegetation history of the high parts of Vitosha mountain during the Late Postglacial times. Fitologija 34: 3–27. |
| 1. Zernitskaya VP (1991) Palaeogeography of the Byelorussian Polessie in the Late Glacial and Holocene. Thesis (Ph. D.) – Minsk: Minsk University. 291 p. |
| 1. Zernitskaya VP (1985) Development of vegetation in the western part of the Kopyl upland during the Late Glacial time and Holocene. In: Geographical aspects of rational nature use. Minsk: Nauka i Taknika. pp. 110–116. |
| 1. Matveev AV, Krutos EA, Zernitskaya VP (1993) Geochronology of the Holocene of the Byelorussian Polessie. Radiocarbon 35: 435–439. |
| 1. Zernitskaya VP, Krutos EA, Klimanov VA (1988) Studies of mire for the purpose of reconstruction of the climatic peculiarities of the Byelorussian Polesie. Minsk: Nauka i Tekhnika. pp. 68–73. |
| 1. Bogdel II (1984) Razvitie prirody Belorrussii v golotsene. Thesis (Ph. D.) – Minsk: Belarusskii Universety. |
| 1. Elovicheva YaK, Bogdel II (1985) Novye razrezy golosena Belarusi. In: Kuznetsov VA, Ropot VF, Elovicheva YaK, editors. Geologicheskoe stroenie osadochnoi tolshchi Belorussii. Minsk: Nauka i Tekhnika. pp. 141–169. |
| 1. Pokorný P, Kuneš P (2005) Holocene acidification process recorded in three pollen profiles from Czech sandstone and river terrace environments. Ferrantia 44: 101–107. |
| 1. Rybníček K, Rybníčková E (1968) The history of flora and vegetation on the Blato mire in southeast Bohemia (palaeoecological study). Folia Geobot 3: 117–142. |
| 1. Rybníčková E (1970) Die entwicklungsgeschichte der Walder auf der Bohmisch-Mahrischen Hohe im Spat- und postglazial. mitt. ostalp. Dinar. Sekt. intern. verein. vegetatioskde, Wien 10/2: 64–68. |
| 1. Rybníček K (1974) Die vegetationsverhaltnisse der moore im sudlichen Teil der bohmisch-Mahrischen Hohe. Vegetace ČSSR A6. Praha: Academia. 236 p. |
| 1. Jankovská V (1980) Paleogeobotanische Rekonstruction der Vegetationsentwicklung im Becker Třeboňská pánev wehrend des Spetglazials und Holozens. Vegetace CSSR, A11 Praha: Academia. |
| 1. Svobodová H (1997) Die Entwicklung der Vegetation in Südmähren (Tschechien) während des Spätglazials und Holozäns - eine palynologische Studie. Verh Zool-Bot Ges Österreich 134: 317–356. |
| 1. Rudolph K, Firbas F (1924) Die Hochmoore des Herzgebirges. Beihefte zum Botanischen Centralblatt 41/2: 1–162. |
| 1. Rudolph K (1926) Pollenanalytische Untersuchungen im thermophilen Florengebiet Bohmens Der Kommerner See. Ber Dtsch Bot Ges 44: 239–248. |
| 1. Losert H (1940) Beitrage zur spät- und nacheiszeitlichen Vegetationsgeschichte Innerbohmens. I. Der "Kommerner See." II. Das Spatglazial von Wschetat. III. Das Spatglazial bei Lissa-Hrabanov. Beihefte zum Botanischen Centralblatt. Original Arbeiten Abt. B, 60: 346–393 . |
| 1. Schmedl H (1940) Beitrag zur Frage des Grenzhorizontes im Sebastiansberger Hochmoor. Beihefte zum Botanischen Centralblatt. Original Arbeiten 60: 493–534. |
| 1. Jankovská V (1983) Palynologische Forschung am ehemaligen Komorany See (Spatglazial bis Subatlantikum). Vestnik UUG 58/2: 99–107. |
| 1. Jankovská V (1988b) Palynologische Erforschung archaologischer Proben aus dem Komoranske jezero See bei Most (NW Bohmen). Folia Geobot 23: 45–78. |
| 1. Břízová E (1995) Reconstruction of the vegetational evolution of Bozi Dar peat bog during Late Glacial Holocene. Praha: Geolines. 10 p. |
| 1. Klapste J, Velimski T (1995) Palaobotanic der mittelalterlichen Stadt Most. Památky Archeologické 86: 81–161. |
| 1. Stebich M, Litt T (1997) Das Georgenfelder Hochmoor ein Archiv fur Vegetations. Siedlungs und Bergbaugeschichte. Leipziger Geowissenschaften 5: 209–216. |
| 1. Hejný S, Slavík B (1998) Květena České socialistické republiky I. Praha: Academia. 558 p. |
| 1. Jankovska V, Kunes P, van der Knaap W (2007) 1. Flaje-Kiefern (Krusne Hory Mountains): Late Glacial and Holocene vegetation development. Grana 46: 214–216. |
| 1. Rybníček K, Rybníčková E (2008) Upper Holocene dry land vegetation in the Moravian-Slovakian borderland (Czech and Slovak Republics). Veg Hist Archaeobot 17: 701–711. |
| 1. Petr L, Pokorný P (2008) Přirozená jezera na území České republiky. Jejich význam pro studium pravěkého osídlení a přírodního prostředí. In: Beneš J, Pokorný P, editors. Bioarcheologie v České Republice. Czech Republic: Jihočeská univerzita v Českých Budějovicích – Archeologický ústav AVČR. pp. 73–98. |
| 1. Svobodová H, Soukupová L, Reille M (2002) Diversified development of mountain mires, Bohemian Forest, Central Europe, in the last 13,000 years. Quatern Int 91: 123–135. |
| 1. Břízová E (1999) Late Glacial and Holocene development of the vegetation in the Labe (Elbe) River flood-plain (Central Bohemia, Czech Republic). Acta Palaeobotanica Suppl. 2: 549–554. |
| 1. Jankovská V, Pokorný P (2008) Forest vegetation of the last full-glacial period in the Western Carpathians (Slovakia and Czech Republic). Preslia 80: 307–324. |
| 1. Rybníčková E, Rybníček K (1979) Syngenesis of Polygalo Nardetum strictae preissing. In: Rychnovska M, editor. Progress report on MAB project no 91: Function of grassland in spring region. Brno. pp. 23–31. |
| 1. Rybníčková E, Rybníček K (1988) Holocene palaeovegetation and palaeoenvironment of the Kamenicska Kotlina basin (Czechoslovakia). Folia Geobot 23: 285–301. |
| 1. Svobodová H, Reille M, Goeury C (2001) Past vegetation dynamics of Vltavský luh, upper Vltava river valley in the Šumava mountains. Czech Republic. Veg Hist Archaeobot 10: 185–199. |
| 1. Pokorný P, Kuneš P (2009) 5. Kožlí (S. Bohemia, Czech Republic). Grana 48: 77–78. |
| 1. Engel Z, Nývlt D, Krízek M, Treml V, Jankovská V, et al. (2010) Sedimentary evidence of landscape and climate history since the end of MIS 3 in the Krkonose Mountains, Czech Republic. Quat Sci Rev 29: 913–927. |
| 1. Treml V, Jankovská V, Petr L (2008) Holocene dynamics of the alpine timberline in the High Sudetes. Biologia 63: 73–80. |
| 1. Jankovská V (1987) Entwicklung des Moores Mokré Louky bei Trebon im postglazial (palaookoligische Studie). Folia Geobot 22: 199–216. |
| 1. Abraham V (2006) Přirozená vegetace a její změny v důsledku kolonizace a lesnického hospodaření v Českém Švýcarsku. Thesis (Ph. D.) – Prague: Charles University of Prague. |
| 1. Svobodova H (1992) The development of the southern Moravian vegetation in the Late Glacial and Holocene. Thesis (Ph. D.) – Czech Republic: Institute of Botany Pruhonice. |
| 1. Jankovská V (1989) The evolution of Late-Glacial and Holocene vegetation in the vicinity of Svetla nad Sazavou (in the western Forland of the Bohemian-Moravian uplands). Folia Geobot 24: 337–448. |
| 1. Jankovská V (2006) Late Glacial and Holocene history of Plešné Lake and its surrounding landscape based on pollen and palaeoalgological analyses. Biologia 61: 371–385. |
| 1. Dudová L, Hájek M, Hájková P (2010) The origin and vegetation development of the Rejvíz pine bog and the history of the surrounding landscape during the Holocene. Preslia 82: 223–246. |
| 1. Rybníčková E, Rybníček K (1985) Palaeogeobotanical evaluation of the Holocene profile from the Rezabinec fishpond. Folia Geobot 20: 419–437. |
| 1. Pokorný P (2005) Role of man in the development of Holocene vegetation in Central Bohemia. Preslia 77: 113–128. |
| 1. Svobodova H (1989) A reconstruction of natural environment and settlement in the environs of Mistrin. A palynological study. Památky Archeologické 80: 188–206. |
| 1. Pokorný P, Jankovská V (2000) Long-term vegetation dynamics and the infilling process of a former lake (Švarcenberk, Czech Republic). Folia Geobot 35: 433–457. |
| 1. Kuneš P, Jankovská V (2000) Outline of Late Glacial and Holocene Vegetation in a Landscape with Strong Geomorphological Gradients. Geolines 11: 112–114. |
| 1. Peichlova M (1979) Historie vegetace Broumovska. Thesis (Ph. D.) – Czech Republic: Institute of Botany Pruhonice.122 p. |
| 1. Rybníčková E, Rybníček K (1972) Erste ergebnisse palae ogeobotanisher. Folia Geobot 7: 285–308. |
| 1. Rybníček K (1983) The environmental evolution and infiling process of a former lake near Vracov (Czeckoslovakia). Hydrobiologia 103: 247–250. |
| 1. Knipping M (1997) Pollenanalytische Untersuchungen zur Siedlungsgeschichte des Oberpfälzer Waldes. Telma 27: 61–74. |
| 1. Hahne J (1992) Untersuchungen zur spät-und postglazialen Vegetationsgeschichte im nordöstlichen Bayern (Bayerisches Vogtland, Fichtelgebirge, Steinwald). Flora 187: 169–200. |
| 1. Rybníčková E (1982) Absolute C14 dates of the profiles from the Zbudovska Blata marshes (southern Bohemia). Folia Geobot 17: 99–100. |
| 1. Rybníčková E, Rybníček K, Jankovská V (1975) Palaeoecological investigation of burried peat profiles from the Zbudovska Blata marshes, southern Bohemia. Folia Geobot 10: 157–178. |
| 1. Kolstrup E (2009) Vegetational and environmental history during the Holocene in the Esbjerg area, west Jutland, Denmark. Veg Hist Archaeobot 18: 351–371. |
| 1. Kolstrup E (1991) A late Pleistocene. Palaeogeogr Palaeoclimatol Palaeoecol 88: 53–69. |
| 1. Behre KE (1976b) Pollenanalytische Untersuchungen zur Vegetations- und Siedlungsgeschichte bei Flögeln und im Ahlenmoor (Elb-Weser-Winkel). Probleme der Küstenforschung 11: 101–118. |
| 1. Behre KE, Kucan D (1986) Die Reflektion archäologisch bekannter Siedlungen in Pollendiagrammen verschiedener Entfernung. – Beispiele aus der Siedlungskammer Flögeln, Nordwestdeutschland. In: Behre K–E, editor. Anthropogenic indicators in pollen diagrams. Bremerhaven: Pangaea. pp. 95–114. |
| 1. Jahns S (2005b) The later Holocene history of vegetation, land-use and settlements around the Ahlequellmoor in the Solling area, Germany. Veg Hist Archaeobot 15: 57–65. |
| 1. Tinner W, Lotter AF (2006) Holocene expansions of Fagus silvatica and *Abies alba* in Central Europe: where are we after eight decades of debate?. Quat Sci Rev 25: 526–549. |
| 1. Rösch M (1989) Pollenprofil Breitnau-Neuhof: Zum zeitlichen Verlauf der holozanen Vegetationsentwicklung im südlichen Schwarzwald. Carolinea 47: 15–24. |
| 1. Willutzki H (1962) Zur Waldgeschichte und Vermoorung sowie über Rekurrenzflächen im Oberharz. Nova Acta Leopold 25(160): 1–52. |
| 1. Beug HJ, Henrion I, Schmüser A (1999) Landschaftsgeschichte im Hochharz - die Entwicklung der Wälder und Moore seit dem Ende der letzten Eiszeit. Chlausthal-Zellerfeld: Papierflieger Verlag. 454 p. |
| 1. Behre KE (1976a) Beginn und Form der Plaggenwirtschaft in Nordwestdeutschland nach pollenanalytishen Untersuchungen in Ostfriesland. Neue Ausgrabungen und Forschungen in Niedersachsen 10:197–224. |
| 1. Rösch M (1986) Zwei Moore im westlichen Bodenseegebiet als Zeugen prahistorischer Landschaftsveranderung. Telma 16: 83–111. |
| 1. Rösch M (1997) Holocene sediment accumulation in the shallow water zone of Lower Lake Constance. Arch Hydrobiol 4: 541–562. |
| 1. Jahns S (2000) Late-glacial and Holocene woodland dynamics and land-use history of the Lower Oder valley, north-eastern Germany, based on two, AMS 14 C-dated, pollen profiles. Veg Hist Archaeobot 9: 111–123. |
| 1. Rösch M (2007) Botanical evidence for prehistoric and medieval land use in Black Forest. In: Klápšte J, Sommer P, editors. Medieval Rural Settlement in Marginal Landscapes, Ruralia 7. Cardiff: Turnhout Brepols Publishers. pp. 335–343. |
| 1. Rösch M (2009) Zur vorgeschichtlichen Besiedlung und Landnutzung im nördlichen Schwarzwald aufgrund vegetationsgeschichtlicher Untersuchungen in zwei Karseen. Mitteilungen des Vereins für Forstliche Standortskunde und Forstpflanzenzüchtung 46: 69–80. |
| 1. Kalis AJ, Merkt J, Wunderlich J (2003) Environmental changes during the Holocene climatic optimum in central Europe-human impact and natural causes. Quat Sci Rev 22: 33–79. |
| 1. Rösch M, Tserendorj G (2011a) Der Nordschwarzwald - früher besiedelt als gedacht? Pollenprofile belegen ausgedehnte vorgeschichtliche Besiedlung und Landnutzung. Denkmalpflege in Baden-Württemberg 40: 66–73. |
| 1. Rösch M, Tserendorj G (2011b) Florengeschichtliche Beobachtungen im Nordschwarzwald (Südwestdeutschland). Hercynia N.F. 44: 53–71. |
| 1. Voigt R, Grüger E, Baier J, Meischner D (2008) Seasonal variability of Holocene climate: a palaeolimnological study on varved sediments in Lake Jues (Harz Mountains, Germany). J Paleolimnol 40: 1021–1052. |
| 1. Jahns S (1999) Ein holozänes Pollendiagramm vom Kleinen Mochowsee, nördliche Niederlausitz. Gleditschia 27: 45–56. |
| 1. Litt T (1994) Paläoökologie, Paläobotanik und Stratigraphie des Jungquartärs im nordmitteleuropäischen Tiefland. Diss Bot 227: 1–185. |
| 1. Bottger T, Hiller A, Junge F, Litt T, Mania D, et al. (1998) Stable isotope, pollen and mollusc analyses of a Late Glacial sequence from Geiseltal, central Germany. Boreas 27: 88–102. |
| 1. Brande A, Böse M, Müller M, Facklam M, Wolters S (1999) The Bliesendorf soil and aeolian sand transport in the Potsdam area. GeoArchaeoRhein 3: 147–161. |
| 1. Wolters S (1999) Spät- und postglaziale Vegetationsentwicklung im Bereich der Fercher Berge Südlichwestlich von Potsdam. Gleditschia 27: 25–44. |
| 1. Jahns S (2007) Palynological investigations into the Late Pleistocene and Holocene history of vegetation and settlement at the Löddigsee, Mecklenburg, Germany. Veg Hist Archaeobot 16: 157–169. |
| 1. Chen SH (1988) Neue Untersuchengen über die spät- und postglaziale Vegetationsgeschichte im Gebiet zwischen Harz und Leine (BDR). Flora 181: 147–177. |
| 1. Jahns S (2004) Ein frühholozänes Pollendiagramm aus dem Tagebau Cottbus-Nord. Verhandlungen des Botanischen Vereins Berlin Brandenburg 137: 79–87. |
| 1. Rösch M (2005) Zur Vegetationsgeschichte des südlichen Kraichgaus - Botanische Untersuchungen bei Großvillars, Gemeinde Oberderdingen, Landkreis Karlsruhe. Fundberichte aus Baden-Wüttemberg 28: 839–870. |
| 1. Bos JAA (2001) Lateglacial and Early Holocene vegetation history of the northern Wetterau and the Amöneburger Basin (Hessen), central-west Germany. Rev Palaeobot Palynol 115: 177–212. |
| 1. Boettger T, Hiller A, Junge FW, Mania D, Kremenetski K (2009) Late Glacial/Early Holocene environmental changes in Thuringia, Germany: Stable isotope record and vegetation history. Quatern Int 203: 105–112. |
| 1. Rösch M (2000) Long-term human impact as registered in an upland pollen profile from the southern Black Forest, south-western Germany. Veg Hist Archaeobot 9: 205–218. |
| 1. Kerig T, Lechterbeck J (2004) Laminated sediments, human impact, and a multivariate approach: a case study in linking palynology and archaeology (Steisslingen, Southwest Germany). Quatern Int 113: 19–40. |
| 1. Jahns S (2001) On the Late Pleistocene and Holocene history of vegetation and human impact in the Ücker valley, north-eastern Germany. Veg Hist Archaeobot 10: 97–104. |
| 1. Dörfler W (1989) Pollenanalytische Untersuchungen zur Vegetations- und Siedlungsgeschichte im Süden des Landkreises Cuxhaven, Niedersachsen. Probleme der Küstenforschung im südlichen Nordseegebiet 17: 1–75. |
| 1. Kühl N (1998) Pollenanalytische Untersuchungen zur Vegetations- und Siedlungsgeschichte in einem Kesselmoor bei Drangstedt, Ldkr. Cuxhaven. Probleme der Küstenforschung im südlichen Nordseegebiet 25: 303–324. |
| 1. Odgaard BV (1988) Heathland history in western Jutland, Denmark. In: Birks HH, Birks HJB, Kaland PE, Moe D, editors. The Cultural Landscape – Past, Present and Future. Cambridge. pp. 311–319. |
| 1. Burjachs F, Pérez–Obiol R, Roure JM, Julia R (1994) Dinámica de la vegetatión durante el Holoceno en la isla de Mallorca. In: Andrés IM, Ollivier MD, Heras JG, Burgaz Moreno ME. Trabajos de palinología básica y aplicada. X simposio de palinología (A.P.L.E., Valencia, septiembre 1994). Valencia: Universitat de Valencia. 199–209. |
| 1. Yll E–I, Pérez–Obiol R, Pantaleon–Cano J, Roure JM (1995) Dinamica del paisaje vegetal en la vertiente Mediterra nea de la peninsula Iberica e islas Baleares desde el tardig glaciar hasta el presente. In: Aleixandre T, Perez A, editors. Reconstituction de la ambient es y cambios climaticos durante el Cuaternarion. Madrid: Centro de Ciencias Medioambientales, CSIC. pp. 319–328. |
| 1. Carrión JS, Fernández S, González-Sampériz P, Gil-Romera G, Badal E, et al. (2010) Expected trends and surprises in the Lateglacial and Holocene vegetation history of the Iberian Peninsula and Balearic Islands. Rev Palaeobot Palynol 3: 458–475. |
| 1. Mariscal B (1993) Variacion de la vegetacion Holocena (4300-280 BP) de Cantabria a traves del analisis polinico de la turbera del Alsa. Estudios Geologicos, Museo Nacional Ciencias Naturales, CSIC, Madrid. |
| 1. Pantaléon-Cano J (1997) Estudi palinologic de sediments litorals de la provincia d'Almeria. Transformacion del paisatge vegetal dins un territori semiarid. Thesis (Ph. D.) – Barcelona: Universitat Autònoma de Barcelona. |
| 1. Pantaléon-Cano J, Yll EI, Pérez-Obiol R, Roure JM (2003) Palynological evidence for vegetational history in semi-arid areas of the western Mediterranean (Almeria, Spain). The Holocene 13:109–119. |
| 1. Penalba MC (1989) Dynamique de vegetation Tardiglaciaire et Holocene du centre-nord de l'Espagne d'après l'analyse pollinique. Thesis (Ph. D.) – Marseille: Aix-Marseille Université. |
| 1. Penalba MC (1994) The history of the Holocene vegetation in northern Spain from pollen analysis. J Ecol 82: 815–832. |
| 1. Pérez-Obiol R, Julia R (1994) Climatic change on the Iberian Peninsula recorded in a 30,000-year pollen record from Lake Banyoles. Quaternary Res 41: 91–98. |
| 1. Carrión J, Munuera M, Dupré M, Andrade A (2001) Abrupt vegetation changes in the Segura mountains of southern Spain throughout the Holocene. J Ecol 89: 783–797. |
| 1. Carrión J, Dupré M (1996) Late Quaternary vegetational history at Navarrés, Eastern Spain. A two core approach. New Phytol 134: 177–191. |
| 1. Fernández S, Fuentes N, Carrión JS, González-Sampériz P, Montoya E, et al. (2007) The Holocene and Upper Pleistocene pollen sequence of Carihuela cave, southern Spain. Geobios 40: 75–90. |
| 1. Carrión J (1992) A palaeoecological study in the western Mediterranean area. The Upper Pleistocene pollen record from Cova Beneito (Alicante, Spain). Palaeogeogr Palaeoclimatol Palaeoecol 92: 1–5, 9–14. |
| 1. Mariscal B (1983) Estudio polinico de la turbera del Cueto de la Avellanosa polaciones (Cantabria). VI Reunion do grupo espanol de traballo de Quaternario. Cadernos do Laboratorio xeolóxico de Laxe 5: 205–226. |
| 1. Taylor D, Pedley H, Davies P, Wright M (1998) Pollen and mollusc records for environmental change in central Spain during the mid-and late Holocene. Holocene 8: 605–612 . |
| 1. Luzón A, Pérez A, Mayayo M, Soria A, Sánchez Goñi M, et al. (2007) Holocene environmental changes in the Gallocanta lacustrine basin, Iberian Range, NE Spain. Holocene 17: 649–663. |
| 1. Davis BAS, Stevenson AC (2007) The 8.2 ky event and early-mid Holocene forests, fires and flooding in the Central Ebro Desert, NE Spain. Quat Sci Rev 26: 1695–1712. |
| 1. García MJG, Valiño, Rodríguez AV, Zapata MBR (2002) Late-glacial and Holocene palaeoclimatic record from Sierra de Cebollera (northern Iberian Range, Spain). Quatern Int 93-94: 13–18. |
| 1. McKeever MH (1984) Comparative palynological studies of two lake sites in western Ireland and northwestern Spain. Thesis (Ph. D.) – Dublin: Trinity College Dublin. |
| 1. Allen JRM, Huntley B, Watts WA (1996) The vegetation and climate of northwest Iberia over the last 14,000 years. J Quaternary Sci 11: 125–147. |
| 1. Sobrino M, Muñoz Sobrino C, Ramil-Rego P, Rodríguez Guitián MA (2001) Vegetation in the mountains of northwest Iberia during the last glacial-interglacial transition. Veg Hist Archaeobot 10: 7–21. |
| 1. Muñoz Sobrino C, Ramil-Rego P, Gómez-Orellana L (2004) Vegetation of the Lago de Sanabria area (NW Iberia) since the end of the Pleistocene: a palaeoecological reconstruction on the basis of two new pollen sequences. Veg Hist Archaeobot 13: 1–22. |
| 1. González-Sampériz P, Valero-Garcés BL, Carrión JS, Peña-Monné JL, García-Ruiz JM, et al. (2005) Glacial and Lateglacial vegetation in northeastern Spain: New data and a review. Quatern Int 140: 4–20. |
| 1. Sánchez-Goñi MF, Hannon GE (1999) High-altitude vegetational pattern on the Iberian Mountain Chain (north- central Spain) during the Holocene. Holocene 9: 39–57. |
| 1. González AV, Saa MP (2000) Analyse pollinique d'une tourbière holocène dans les Montes do Buio: Cuadramón (Galice, nord-ouest de l'Espagne). Quaternaire 11: 257–268. |
| 1. Carrión JS, van Geel B (1999) Fine-resolution Upper Weichselian and Holocene palynological record from Navarrés (Valencia, Spain) and a discussion about factors of Mediterranean forest succession. Rev Palaeobot Palynol 106: 209–236. |
| 1. Stevenson A (2000) The Holocene forest history of the Montes Universales, Teruel, Spain. Holocene 10: 603– 610. |
| 1. Mariscal B (1986) Analisis polinico de la turbera del Pico del Sertal de la sierra de Pena Labra. Reconstrucion de la paleoflora y de la climatologia durante el Holoceno en la zona oriental de la cordillera cantabria. In: Lopez-Vera F, editor. Quaternary climate in Western Mediterranean. Madrid: Universidad Autonoma de Madrid: 205–220. |
| 1. Muñoz Sobrino C, Ramil-Rego P, Rodríguez Guitián M (1997) Upland vegetation in the north-west Iberian peninsula after the last glaciation: forest history and deforestation dynamics. Veg Hist Archaeobot 6: 215–233. |
| 1. López-Merino L, Silva Sánchez N, Kaal J, López-Sáez JA, Cortizas AM (2012) Post-disturbance vegetation dynamics during the Late Pleistocene and the Holocene: An example from NW Iberia. Glob Planet Change 92–93: 58–70. |
| 1. Mariscal B (1989) Comparacion palinologica entre una turbera de la cordillera central y unas turberas de la cordillera cantabrica. II. European Paleobot. Conference. Madrid: Univ. Complutense Madrid. |
| 1. Mugica FF, Anton MG, Ollero HS (1998) Vegetation dynamics and human impact in the Sierra de Guadarrama, Central System, Spain. Holocene 8: 69–82. |
| 1. Yll E–I, Roure J–M, Pantaleon–Cano J, Pérez–Obiol R (1994) Analisis polinico de una secuencia holocenica en Roquetas de Mar (Almeria). In: Mateu I, Dupre M, Guemes J, Burgaz ME, editors. Trabajos de palinologia basica y aplicada. Valencia: Universitat de Valencia. pp. 189–198. |
| 1. Pantaléon-Cano J, Yll EI, Pérez-Obiol R, Roure JM (2003) Palynological evidence for vegetational history in semi-arid areas of the western Mediterranean (Almeria, Spain). The Holocene 13:109–119. |
| 1. Hannon GE (1985) Late Quaternary vegetation of Sanabria Marsh. Thesis (Ph. D.) – Dublin: Trinity College Dublin. |
| 1. Turner C, Hannon GE (1988) Vegetational evidence for late Quaternary climate changes in southwest Europe in relation to the influence of the North Atlantic Ocean. Philos Trans R Soc Lond B Biol Sci 318: 451–485. |
| 1. Carrion JS, Sánchez-Gomez P, Mota JF, Yll R, Chaín C (2003) Holocene vegetation dynamics, fire and grazing in the Sierra de Gádor, southern Spain. Holocene 13: 839–851. |
| 1. Iriarte M (2009) Vegetation landscape and the anthropization of the environment in the central sector of the Northern Iberian Peninsula: Current status. Quatern Int 200: 66–76. |
| 1. Saarse L, Liiva A (1995) Geology of the Äntu group of lake. Proceedings of the Estonian Academy of Science, Geology 44: 119–132. |
| 1. Saarse L, Niinemets E, Amon L, Heinsalu A, Veski S, et al. (2009) Development of the late glacial Baltic basin and the succession of vegetation cover as revealed at Palaeolake Haljala, northern Estonia. Est J Earth Sci 58: 317–333. |
| 1. Kimmel K, Pirrus R, Raukas A (1999) Holocene deposits. In: Miidel A, Raukas A, editors. Lake Peipsi. Geology. Tallinn: Institute of Geology at Tallinn Technical University. Sulemees Publishers. pp. 42–52. |
| 1. Saarse L, Rajamäe R (1997) Holocene vegetation and climatic change on the Haanja Heights, SE Estonia, Proceedings of the Estonian Academy of Science. Geology 46: 75–94. |
| 1. Heinsalu A, Veski S (1991) History of Lake Ermistu. In: Inimene ja Geografiline Kesk. Tallinn: Vaba., EESTI TA. pp. 14–18 . |
| 1. Saarse L, Vishnevskaya E, Sarv A (1990b) Geological development of lakes, Island Saaremaa . Proceedings of the Estonian Academy of Science, Biology 39: 34–47. |
| 1. Saarse L, Königsson LK (1992) Holocene environmental changes on the island of Saaremaa, Estonia. Pact 37: 97–131. |
| 1. Saarse L, Veski S, Rajamäe R, Sarv A, Heinsalu A (1990a) Geology of Lake Maardu, Eesti Teaduste. Tallinn: Akademia Geoloogia Instituut. 32 p. |
| 1. Poska A, Saarse L (2002) Biostratigraphy and 14 C dating of a lake sediment sequence on the north-west Estonian carbonaceous plateau, interpreted in terms of human impact in the surroundings. Veg Hist Archaeobot 11: 191–200. |
| 1. Punning JM, Kangur M, Koff T, Possnert G (2003) Holocene lake-level changes and their reflection in the paleolimnological records of two lakes in northern Estonia. J Paleolimnol 29: 167–178. |
| 1. Niinemets E, Saarse L (2009) Holocene vegetation and land-use dynamics of south-eastern Estonia. Quatern Int 207: 104–116. |
| 1. Koff T (1990) Reconstruction of palaeogeographical conditions in NE Estonia on the basis of bog and lake deposits. In: Hult J, editor. Estonia-Finnish seminar on environmental questions, Joensuu, 21.–22. 8. 1989. pp. 99–115. |
| 1. Hansson A-M, Hiie S, Kihno K, Masauskaité R, Moe D, et al. (1996) A vegetation historical study of Johvikasoo, an ombrogenous mire at Tuiu, Saaremaa, Estonia. Pact 51: 39–57. |
| 1. Sarv A, Il'ves EO (1975) Über das Alter der holozänen Ablagerungen im Mündungsgebiet des Flusses Emajogi (Saviku). Eesti NSV Teaduste Akademia, Toimetised, Keemia Geol. 24: 64–71. |
| 1. Saarse L, Veski S, Heinsalu A, Rajamäe R, Martma T (1995) Litho- and biostratigraphy of Lake Päidre, South Estonia. Proceedings of the Estonian Academy of Sciences. Geology 44: 45–59. |
| 1. Niinemets E, Saarse L (2007) Mid-and late-Holocene land-use changes inferred from pollen records, in a south-eastern Estonian upland area. Rev Palaeobot Palynol 146: 51–73. |
| 1. Pirrus R, Rouk A–M, Liiva A (1987) Geology and stratigraphy of the reference site of Lake Raigastvere in Saadjärv drumlin field. In: Raukas A, Saarse L, editors. Palaeohydrology of the temperate zone III. Mires and lakes. Tallinn: Valgus. pp. 101–122. |
| 1. Kimmel K, Rajamäe R, Sakson M (1996) The Holocene development of Tondi mire, northern Estonia: pollen, diatom and chronological studies. PACT 51: 85–102. |
| 1. Hyvärinen H (1975) Absolute and relative pollen diagrams from northernmost Fennoscandia. Fennia 142: 1–23. |
| 1. Rankama T, Vuorela I (1988) Between inland and coast in Metal Age Finland - human impact on the premeval forests of Southern Hame during the Iron Age. Memoranda Societatis Pro Fauna et Flora Fennica 64: 25–34. |
| 1. Sarmaja-Korjonen K, Alhonen P (1999) Cladoceran and diatom evidence of lake-level fluctuations from a Finnish lake and the effect of acquatic-moss layers on microfossil assemblages. J Paleolimnol 22: 277–290. |
| 1. Asplund H, Vuorela I (1989) Settlement studies in Kemiö - archaeological problems and palynological evidence. Fennoscandia Archaeologica 6: 67–79. |
| 1. Vuorela I (1981) The vegetational and settlement history in Sysmä, central south Finland, interpreted on the basis of two pollen diagrams. Bulletin of the Geological Society of Finland 53: 47–63. |
| 1. Hicks S (1975b) Variations in pollen frequency in a bog at Kangerjoki, N.E. Finland during the Flandrian. Commentationes Biologicae 80: 4–28. |
| 1. Hicks S (1988a) Oulun yliopisto, historian laitos. Eripainossarja 202: 35–88. |
| 1. Hicks S (1988b) The representation of different farming practices in pollen diagrams from northern Finland. In: Birks HH, Birks HJB, Kaland PE, Moe D, editors. The Cultural Landscape – Past, Present and Future. Cambridge. pp. 189–207. |
| 1. Hicks S (1992) Modern pollen deposition and its use in interpreting the occupation history of the island Hailuto Finland. Veg Hist Archaeobot 1: 75–86. |
| 1. Jauhiainen S, Pitkainen A, Vasanderl H (2004) Chemostratigraphy and vegetation in two boreal mires during the Holocene. Holocene 14: 769–779. |
| 1. Vuorela I (2010) Pollen profile KUIVAJAR, Kuivajarvi, Finland. European Pollen Database (EPD) |
| 1. Sarmaja-Korjonen K, Vasari Y, Haeggström C-A (1991) Taxus baccata and influence of Iron Age man on the vegetation in Âland, SW Finland. Ann Bot Fenn 28: 143–159. |
| 1. Väliranta M, Kultti S, Nyman M, Sarmaja-Korjonen K (2005) Holocene development of aquatic vegetation in shallow Lake Njargajavri, Finnish Lapland, with evidence of water-level fluctuations and drying. J Paleolimnol 34: 203–217. |
| 1. Seppä H (1996) Post-glacial dynamics of vegetation and tree-lines in the far north of Fennoscandia. Fennia 174: 1–96. |
| 1. Szeroczyńska K, Tatur A, Weckström J, Gąsiorowski M, Noryśkiewicz AM, et al. (2007) Holocene environmental history in northwest Finnish Lapland reflected in the multi-proxy record of a small subarctic lake. J Paleolimnol 38: 25–47. |
| 1. Seppä H, Birks HJB (2001) July mean temperature and annual precipitation trends during the Holocene in the Fennoscandia tree-line area: pollen based climate reconstruction. Holocene 11: 527–539. |
| 1. Seppä H, Hicks S (2006) Integration of modern and past pollen accumulation rate (PAR) records across the arctic tree-line: a method for more precise vegetation reconstructions. Quat Sci Rev 25: 1501–1516. |
| 1. Vuorela I (1990) Pollenanalytiska studier. In: Zilliachus K, editor. Studier i åboländsk kulturhistoria utgivna av Konstsamfundet. Helsinki. pp. 115–134. |
| 1. Vasari Y (1965) Studies on the vegetational history of the Kuusamo district (north east Finland) during the late quaternary period. III. Maanselänsuo, a late-glacial site in Kuusamo. Ann Bot Fenn 2: 219–235. |
| 1. Hyvärinen H (1992) Holocene pine and birch limits near Kilpisjärvi, Western Finnish Lapland: pollen stratigraphical evidence. Paläoklimaforschung 9: 19–27. |
| 1. Eronen M, Hyvärinen H (1982) Subfossil pine dates and pollen diagrams from northern Fennoscandia. Geologiska Föreningens i Stockholm Förhandlinger 103: 437–455. |
| 1. Hicks S (1974) A method of using modern pollen rain values to provide a time-scale for pollen diagrams from peat deposits, Memoranda Soc. Fauna. Flora Fennica 49: 21–33. |
| 1. Hicks S (1985) Problems and possibilities in correlating historical/archaeological and pollen-analytical evidence in a northern boreal environment: An example from Kuusamo Finland. Fennoscandia Archaeologica 2: 51–84. |
| 1. Hicks S (1975a) New evidence for the presence of prehistoric man in the Kuusamo area. Nordia 1: 1–16. |
| 1. Hicks S (1976) Pollen analysis and archaeology in Kuusamo, N.E. Finland, an area of marginal human interference. Transactions of the Institute of British Geographers New Series 1: 361–386. |
| 1. Vuorela I (1991) Lounais-Suomen varhaismetallikautinen asutus ja viljely siitepölyanalyanalyysin valossa. Karhunhammas 13: 2–23. |
| 1. Huttunen A (1990) Vegetation and palaeoecology of a bog complex in southern Finland. Aquilo Ser Bot 28: 27–37. |
| 1. De Valk EJ (1981) Late Holocene and present vegetation of the Kastelberg (Vosges, France). Thesis (Ph. D.) – The Netherlands: University of Utrecht. |
| 1. de Beaulieu JL, Goeury C (1987) Zonation automatique appliquée à l'analyse pollinique: exemple de la narse d'Ampoix (Puy de Dome, France). Bulletin de l'Association Française pour l'Etude du Quaternaire 1: 49–61. |
| 1. Juvigné E, Gewelt M (1987) La narse d'Ampoix comme Téphrostratotype dans la chaine des Puys Méridionale (France). Bulletin de l'Association Française pour l'Etude du Quaternaire 1: 37–49. |
| 1. Coutard S (2003) Formations quaternaires en bordure d'une mer épicontinentale, climat et occupations humaines. Exemple du Val de Saire (Normandie, France). Thesis (Ph. D.) – Caen: Université de Caen. |
| 1. Coutard S, Clet–Pellerin M (2006) Evolution de la sédimentation et de la végétation pendant l'Holocene dans les marais arrière–littoraux du Val de Saire (Cotentin, Normandie). In: Allée P, Lespez L, editors. L’érosion entre Société, Climat et Paléoenvironnement. Actes de la Table Ronde en l’honneur de René Neboit Guilhot. Collection «Nature et Société, 3. PressesUniversitaires Blaise Pascal, pp. 273–280. |
| 1. Lespez L, Hardel B, Clet-Pellerin M, Davidson R, Marcigny C (2005a) Evolution des paysages du Néolithique à nos jours dans la péninsule de La Hague (Normandie, France), l'exemple de l'Anse St-Martin. Archéologie, histoire et anthropologie de la presqu'île de La Hague (Manche). Première année de recherche 2005 (2005) 31–43. |
| 1. Reille M, Lowe JJ (1993) A re-evaluation of the vegetation history of the eastern Pyrenees (France) from the end of the last glacial to the present. Quat Sci Rev 12: 47–77. |
| 1. Garnaud S, Lesueur P, Clet-Pellerin M, Lesourd S, Garlan T, et al. (2003) Holocene to modern fine-grained sedimentation on macrotidal shoreface-to -inner-shelf setting (eastern Bay of the Seine, France). Mar Geol 202: 33–54. |
| 1. Delsinne N (2005) Evolution pluri-millénaire à pluriannuelle du prisme sédimentaire d'embouchure de la Seine. Thesis (Ph. D.) – Caen: Université de Caen. |
| 1. Tessier B, Delsinne N, Sorrel P (2010) Holocene sedimentary infilling of a tide-dominated estuary mouth. The example of macrotidal Seine estuary (NW France). Bulletin de la Societe Geologique de France 181: 87–98. |
| 1. Voeltzel D (1987) Recherches pollenanalytiques sur la vegetation holocene de la plaine alluviale de l'estuaire de la Loire et des coteaux environnants. Thesis (Ph. D.) – Marseille: Aix-Marseille Université III.186 p. |
| 1. Bégeot C (2000) Histoire de la végétation et du climat au cours du Tardiglaciaire et du début de l'Holocene sur le Massif jurassien central à partir de l'analyse pollinique et l'étude des macrorestes végétaux. Thesis (Ph. D.) – France. |
| 1. Nicol-Pichard S, Dubar M (1998) Reconstruction of late-glacial and holocene environments in southeast France based on the study of a 66-m long core from Biot, Alpes Maritimes. Veg Hist Archaeobot 7: 11–15. |
| 1. Lespez L, Clet-Pellerin M, Limondin-Lozouet N, Pastre JF, Fontugne M (2005b) Discontinuités longitudinales des dynamiques sédimentaires holocènes dans les petites vallées de l'Ouest du Bassin Parisien, l'exemple de la Mue (Basse-Normandie). Quaternaire 16: 273–298. |
| 1. Lespez L, Cador JM, Carpentier V, Clet–Pellerin M, Germaine MA, et al. (2008a) Trajectoire des paysages des vallées normandes et gestion de l'eau du Néolithique aux enjeux de la gestion contemporaine. In: Galop D, editor. Paysage et environnement: de la reconstitution du passé aux modèles prospectifs. Besançon: Presses Universitaires de Franche-Comté, Annales littéraires, série Environnement, Société et Archéologie. pp. 61–75. |
| 1. Lespez L, Clet-Pellerin M, Limondin-Lozouet N, Pastre JF, Fontugne M, et al. (2008b) Fluvial system evolution and environmental changes during the Holocene in the Mue valley (Western France). Geomorphology 98: 55–70. |
| 1. de Beaulieu JL (1977) Contribution pollenanalytique à l'histoire tardiglaciaire et Holocene de la végétation des Alpes méridionales françaices. Thesis (Ph. D.) – Marseille: Aix-Marseille Université III. |
| 1. David F (1993) Altitudinal variation in the response of the vegetation to Late-glacial climatic events in the northern French Alps. New Phytol 125: 203–220. |
| 1. Wegmüller S (1977) Pollenanalytische Untersuchungen zur spät- und postglazialen Vegetationsgeschichte der französichen Alpen (Dauphiné). Bern: Verlag Paul Haupt. 185 p. |
| 1. Nakagawa T (1998) Etudes palynologiques dans les Alpes Françaises centrales et méridionales, histoire de la végétation Tardiglaciaire et Holocène. Thesis (Ph. D.) – Marseille: Aix-Marseille Université. |
| 1. Puertas O (1998) Perception palynologique de la gestion du paysage autour de la cité protohistorique de Lattara (Lattes France). Revue d'Archéométrie 2: 23–27. |
| 1. Bernard J (1996) Paléoenvironnement du Pays de Retz et du marais breton-vendéen. Thesis (Ph. D.) – Nantes: Université de Nantes. |
| 1. Reille M, de Beaulieu JL (1981) Analyse pollinique de l'étang de Cheylade (Massif Central, France): histoire tardiglaciaire et holocène de la végétation de la planèze de Saint-Flour. C R Seances Acad Sci III 292: 243–246. |
| 1. Sánchez–Goñi MF (1995) Analyse palynologique de l'Etang d'Ouveillan. In: Guilaine I, editor. Temps et espace dans le bassin de l'Aude du néolithique à l'âge du fer. Toulouse. pp. 265–275. |
| 1. de Beaulieu JL, Leveau P, Miramont C, Palet, JM, Walsh K, et al. (2003) Changements environnementaux postglaciaires et action de l'homme dans le bassin du Buëch et en Champsaur (Hautes-Alpes, France). Premier bilan d'une étude pluridisciplinaire. In: Muxart A, Vivien F-D, Villalba B, Burnouf J, editors. Des Milieux et des Hommes: Fragments d'Histoires Croisées. Paris: Elsevier. pp. 93–101. |
| 1. Clet M, Helluin M, Pellerin J, Pilet-Lemiere J, Fontugne M (1993) L'evolution des environnements végétaux pendant les deux derniers millénaires dans la region de Fougers (Ille-et-Vilaine, France). Palynosciences 2: 39–55. |
| 1. Visset L (1979) Recherches palynologiques sur la végétation Pleistocène et Holocène de quelques sites du district phytogéographique de Basse-Loire. Thesis (Ph. D.) – Nantes: Société des sciences naturelles de l'Ouest de la France. |
| 1. Clerc J (1988) Recherches pollenanalytiques sur la paléo-écologie Tardiglaciaire et Holocene du Bas-Dauphiné. Thesis (Ph. D.) – France: Université Paul Cézanne. |
| 1. Barbier D (1999) Histoire de la vegetation du nord-mayennais de la fin du Weichselien à l'aube du XXIème siècle, armoricain; interactions homme-milieu. Thesis (Ph. D.) – Nantes: Université de Nantes. |
| 1. Fauquette S, Guiot J, Menut M, de Beaulieu JL, Reille M, et al. (1999) Vegetation and climate since the last interglacial in the Vienne area (France). Glob Planet Change 20: 1–17. |
| 1. Clet–Pellerin M, Dugué O, Lautridou JP (2006) Seuil du Cotentin sédiments plio–pléistocenes comblement des marais |
| 1. Gauthier E (2001) Evolution de l'impact de l'homme sur la végétation du massif jurassien au cours des quatre derniers millénaires. Nouvelles données palynologiques. Thesis (Ph. D.) – France: Université de Franche-Comté. |
| 1. de Beaulieu, JL., Pons A, Reille M (1982) Recherches pollenanalytiques sur l'histoire de la végétation de la bordure nord du Massif du Cantal (Massif-Central, France). Pollen et Spores 24: 251–300. |
| 1. Reille M, Gamisans J, Beaulieu JL, Andrieu V (1997) The late-glacial at Lac de Creno (Corsica, France): a key site in the western Mediterranean basin. New Phytol 135: 547–559. |
| 1. Reille M, Gamisans J, Andrieu-Ponel V, de Beaulieu JL (1999) The Holocene at Lac de Creno, Corsica, France: a key site for the whole island. New Phytol 141: 291–309. |
| 1. Reille M, de Beaulieu JL (1988) History of the Würm and Holocene vegetation in western Velay (Massif Central, France): a comparison of pollen analysis from three corings at Lac du Bouchet. Rev Palaeobot Palynol 54: 233–248. |
| 1. Thouveny N, Creer KM, Blunk I (1990) Extention of the Lac du Bouchet palaeomagnetic record over the last 120,000 years. Earth Planet Sci Lett 97: 140–161. |
| 1. Argant J, Argant A (2000) Mise en évidence de l'occupation ancienne d'un site d'altitude: analyse pollinique du lac de Lauzon (Drôme). Géologie Alpine 31: 61–71. |
| 1. Noël H, Garbolino E, Brauer A, Lallier-Vergès E, de Beaulieu JL, et al. (2001) Human impact and soil erosion during the last 5000 yrs as recorded in lacustrine sedimentary organic matter at Lac d'Annecy, the French Alps. J Paleolimnol 25: 229–244. |
| 1. Argant J (1990) Climat et environnement au Quaternaire dans le bassin du Rhône d'apres les données palynologiques. Documents du Laboratoire de Géologie de Lyon 3: 1–199. |
| 1. van Zeist W, van der Spoel-Walvius MR (1980) A palynological study of the Late-Glacial and the Postglacial in the Paris basin. Palaeohistoria 22: 67–109. |
| 1. Reille M, de Beaulieu JL, Pons A (1985) Recherches pollenanalytiques sur l'histoire tardiglaciaire et Holocène de l a végétation du Cézalier, de la Planèze de St-Flour et de la Margeride (Massif-Central, France). Pollen et Spores 27: 209–270. |
| 1. Billard C, Clet-Pellerin M, Lautridou JP, Giffault M (1995) Un site protohistorique littoral dans le havre de la Vanlee à Lingreville et Bricqueville-sur-mer (Manche). Revue Archéologique de l'Ouest 12: 73–110. |
| 1. Cyprien AL, Visset L, Carcaud N (2004) Evolution of vegetation landscapes during the Holocene in the central and downstream Loire basin (Western France). Veg Hist Archaeobot 13: 181–196. |
| 1. Lespez L, Clet-Pellerin M, Davidson R, Hermier G, Carpentier V, et al. (2010) Middle to Laye Holocene landscape changes and geoarchaeological implications in the marshes of the Dives estuary (NW France). Quatern Int 216: 23–40. |
| 1. Bigot F, Fosse G, Lautridou JP, Truffreau A, Verron G (1976) Néolithique et Ages des Métaux Données nouvelles. In: Bigot F, editor. Préhistoire de la Normandie. Caen: Direction des antiquités préhistoriques de Basse-Normandie. pp. 93–102. |
| 1. Verron G (1977) Le marais de Marchésieux. Découvertes de l' âge du Bronze et étude palynologique des tourbes. Gallia Préhistoire 20–22: 370–374. |
| 1. van Zeist W (1963) Recherches palynologiques en Bretagne Occidentale, Congrès Préhistorique de France. Rennes 37: 5–19. |
| 1. van Zeist W (1964) A paleobotanical study of some bogs in western Brittany (Finistère), France. Palaeohistoria 10: 157–180. |
| 1. Muller SD, David F,Wicha S (2000) Impact de l'exposition des versants et de l'anthropisation sur la dynamique forestiére dans les alpes du sud (France). Géographie Physique et Quaternaire 54: 231–243. |
| 1. Guiter F, Andrieu-Ponel V, Digerfeldt G, Reille M, de Beaulieu JL, et al. (2005) Vegetation history and lake-level changes from the Younger Dryas to the present in Eastern Pyrenees (France): pollen, plant macrofossils and lithostratigraphy from Lake Racou (2000 m asl). Veg Hist Archaeobot 14: 99–118. |
| 1. Puertas O (1997) Evolution de la végétation depuis le Dryas récent dans la plaine littorale de Montpellier (Herault France) a partir de l'analyse pollinique. Thesis (Ph. D.) – Besançon: Université de Franche-Comté. |
| 1. Gandouin E, Ponel P, Andrieu-Ponel V, Guiter F, de Beaulieu JL, et al. (2009) 10,000 years of vegetation history of the Aa palaeoestuary, St-Omer Basin, northern France. Rev Palaeobot Palynol 156: 307–319. |
| 1. Pulido M (2006) Conséquenses de l'anthropisation sur la dynamique postglaciaire de la végétation dans le sud du Massif Central, France. Thesis (Ph. D.) – Marseille: University of Axis-Marseille III. |
| 1. Planchais N (1970) Tardiglaciaire et Postglaciaire à Mur-de -Sologne (Loir-et-Cher). Paris: CNRS. 48 p. |
| 1. de Beaulieu JL, Guiot J (1990) Mur de Sologne. In: Rapport CEA.BC.4548, 1 diagramme (inédit). pp. 11–18. |
| 1. Jouffroy-Bapicot I, Pulido M, Baron S, Galop D, Monna F, et al. (2007) Environmental impact of early palaeometallurgy: pollen and geochemical analysis. Veg Hist Archaeobot 16: 251–258. |
| 1. Lespez L, Clet–Pellerin M, Levalet F, Bellec F, Davidson R (2003) L'apport des archives naturelles dans la compréhension de l'évolution des paysages proto-historiques et historiques normands, l'exemple de la Péninsule de La Hague (manche). In: Annales de Normandie. Série des congrès des sociétés historiques et archéologiques de Normandie. pp. 77–93. |
| 1. Lespez L, Clet-Pellerin M, Davidson R, Marcigny C (2004) Evolution des paysages et anthropisation depuis le Néolithique dans la péninsule de la Hague (Normandie, France). Revue d'Archéométrie 28: 71–88. |
| 1. Lespez L, Clet-Pellerin M, Davidson R, Desmarest T, Menesson B, et al. (2006) L'environnement de la Mare de Vauville, de l'étude de la longue durée au développement durable: premiers résultats. In: Marcigny C, Ghesquière d’E, editors. Archéologie, histoire et anthropologie de la presqu᾽île de la hague (manche). Le Tourp: Communauté de Communes de la Hague. pp. 7–14. |
| 1. Jóhansen J (1982) Vegetational development in the Faroes from 10 kyr BP to the present. Danmarks Geologiske Undersogelser (Geological Survey of Denmark) Yearbook 1981 (1982): 111–136. |
| 1. Birks HH, Mathewes RW (1978) Studies in the vegetation history of scotland. V. Late Devensian and early Flandrian pollen and macrofossil stratigraphy at Abernethy forest, Inverness-shire. New Phytol 80: 455–484. |
| 1. Birks HH (1975) Studies in the vegetational history of Scotland. IV. Pine stumps in Scottish blanket peats. Philos Trans R Soc Lond B Biol Sci 270: 181–226. |
| 1. Watkins R, Scourse JD, Allen JRM (2007) The Holocene vegetation history of the Arfon Platform, North Wales, UK. Boreas 36: 170–183. |
| 1. Whittington G, Edwards KJ, Cundill PR (1991a) Late-and post-glacial vegetational change at Black Loch, Fife, eastern Scotland-a multiple core approach. New Phytol 118: 147–166. |
| 1. Kelly A, Charman DJ, Newnham RM (2010) A Last Glacial Maximum pollen record from Bodmin Moor showing a possible cryptic northern refugium in southwest England. J Quaternary Sci 25: 296–308. |
| 1. Waller MP, Marlow A (1994) Flandrian vegetational history of south-eastern England. Stratigraphy of the Brede valley and pollen data from Brede Bridge. New Phytol 126: 369–392. |
| 1. Mighall T, Chambers F (1995) Holocene vegetation history and human impact at Bryn y Castell, Snowdonia, north Wales. New Phytol 130: 299–321. |
| 1. Holland SM (1975) A pollen-analytical study concerning settlement and early agriculture in County Down. Thesis (Ph. D.) – Belfast: Queen's University. |
| 1. Pennington W (1975) A chronostratigraphic comparison of late Weichselian and late Devensian subdivisions illustrated by two radiocarbon dated profiles from western Britain. Boreas 4: 157–171. |
| 1. Waller MP, Hamilton S (2000) Vegetation history of the English chalklands: a mid-Holocene pollen sequence from the Caburn, East Sussex. J Quaternary Sci 15: 253–272. |
| 1. Whittington G, Buckland P, Edwards K, Greenwood M, Hall A M, Robinson M (2003) Multiproxy Devensian Late‐glacial and Holocene environmental records at an Atlantic coastal site in Shetland. J Quaternary Sci 18: 151–168. |
| 1. Bennett K, Boreham S, Sharp M, Switsur V (1992) Holocene history of environment, vegetation and human settlement on Catta Ness, Lunnasting, Shetland. J Ecol 80: 241–275. |
| 1. Innes J, Rutherford M, O'Brien C, Bridgland D, Mitchell W, et al. (2009) Late Devensian environments in the Vale of Mowbray, North Yorkshire, UK: evidence from palynology. Proc Geol Assoc 120: 199–210. |
| 1. Shotyk W (1996) Peat bog archives of atmospheric metal deposition: geochemical evolution of peat profiles, natural variations in metal concentrations, and metal enrichment factors. Environ Rev 4: 149–185. |
| 1. Shotyk W, Cheburkin AK, Appleby PG, Fankhauser A, Kramers JD (1997) Lead in three peat bog profiles, Jura Mountains, Switzerland: enrichment factors, isotopic composition, and chronology of atmospheric deposition. Water Air Soil Pollut 100: 297–410. |
| 1. Keatinge T, Dickson J (1979) Mid-Flandrian changes in vegetation on Mainland Orkney. New Phytol 82: 585–612. |
| 1. Fyfe RM (2007) The importance of local-scale openness within regions dominated by closed woodland. J Quaternary Sci 22: 571–578. |
| 1. Yeloff D, van Geel B, Broekens P, Bakker J, Mauquoy D (2007) Mid-to late-Holocene vegetation and land-use history in the Hadrian's Wall region of northern England: the record from Butterburn Flow. Holocene 17: 527–538. |
| 1. Brown AP (1972) Late-Weichselian and Flandrian vegetation of Bodmin Moor, Cornwall. Thesis (Ph. D.) – Cambridge: University of Cambridge. 214 p. |
| 1. Brown AP (1977) Late-Devensian and Flandrian vegetational history of Bodmin Moor, Cornwall. Philos Trans R Soc Lond B Biol Sci 276: 251–946. |
| 1. Hicks S (1969) The pollen-analytical evidence for the impact of agriculture on the vegetation of a Gritstone upland in North Derbyshire. Thesis (Ph. D.) – Leeds: University of Leeds.165 p. |
| 1. Hicks S (1971) Pollen-analytical evidence for the effect of prehistoric agriculture on the vegetation of North Derbyshire. New Phytol 70: 647–667. |
| 1. Hicks S (1972) The impact of man on the East Moor of Derbyshire from Mesolithic times. The Archaeological Journal 129: 1–21. |
| 1. Waller MP (1994) The Fenland Project No. 9: Flandrian environmental change in Fenland. Cambridge: East Anglian Archaeology. 353 p. |
| 1. Godwin H, Tallantire PA (1951) Studies of the Post-glacial history of British vegetation XII. Hockam Mere, Norfolk. J Ecol 39: 285–307. |
| 1. Bennett K, (1983) Devensian late-glacial and Flandrian vegetational history at Hockham Mere, Norfolk, England. I. Pollen percentages and concentrations. New Phytol 95: 457–487. |
| 1. Waller MP, Long A, Long D, Innes J (1999) Patterns and processes in the development of coastal mire vegetation: Multi-site investigations from Walland Marsh, Southeast England. Quat Sci Rev 18: 1419–1444. |
| 1. Bartley DD, Morgan AV (1990) The palynological record of the King's Pool, Stafford. New Phytol 116: 177–194. |
| 1. Hirons KR, Edwards KJ (1990) Pollen and related studies at Kinloch, Isle of Rhum, Scotland, with particular reference to possible early human impacts on vegetation. New Phytol 116: 715–728. |
| 1. Hulme P, Shirriffs J (1994) The Late-glacial and Holocene vegetation of the Lang Lochs Mire area, Gulberwick, Shetland: a pollen and macrofossil investigation. New Phytol 128: 793–806. |
| 1. Birks HJB (1973) Past and present vegetation of the isle of Skye: A palaeoecological study. London: Cambridge University Press. 427 p. |
| 1. Birks HJB, Madsen BJ (1979) Flandrian vegetational history of Little Loch Roag, Isle of Lewis, Scotland. J Ecol 67: 825–844. |
| 1. Walker MJC, Harkness DD (1990) Radiocarbon dating the Devensian Lateglacial in Britain: New evidence from Llanilid, south Wales. J Quaternary Sci 5: 135–144. |
| 1. Harkness DD, Walker MJC (1991) The Devensian Lateglacial carbon isotope record from Llanilid, south Wales. In: Lowe JJ, editor. Radiocarbon Dating: Recent Applications and Future Potential. Cambridge: Quaternary Proceedings 1, QRA. pp. 35–44. |
| 1. Lowe S (1981) Radiocarbon dating stratigraphic resolution in Welsh lateglacial chronology. Nature 293: 210–212. |
| 1. Lowe JJ, Lowe S, Fowler AJ, Hedges REM, Austin TJF (1988) Comparison of accelerator and radiometric radiocarbon measurements obtained from Late Devesian late-glacial lake sediments from Gwernan, north Wales. Boreas 17: 355–369. |
| 1. Lowe JJ, Lowe S (1989) Interpretation of the pollen stratigraphy of late Devensian late-glacial and early Flandrian sediments of Lhym Gwenan near Cader Idris, north Wales. New Phytol 113: 391–408. |
| 1. Fossitt J (1996) Late Quaternary vegetation history of the Western Isles of Scotland. New Phytol 132: 171–196. |
| 1. Edwards KJ, Berridge JMA (1994) The late-quaternary vegetational history of Loch Abhogaidh, Rinns of Islay SSSI, Scotland. New Phytol 128: 749–770. |
| 1. Pennington W (1977) The Late Devensian flora and vegetation of Britain. Philos Trans R Soc Lond B Biol Sci 280: 247–271. |
| 1. Williams W (1976) The Flandrian vegetational history of the Isle of Skye and the Morar Peninsula. Thesis (Ph. D.) – Cambridge: University of Cambridge. |
| 1. Birks HJB, Williams W (1983) Late-Quaternary vegetational history of the Inner Hebrides. Proc R Soc Edinb Biol 83: 269–292. |
| 1. Pennington W, Haworth EY, Bonny AP, Lishman JP (1972) Lake sediments in northern Scotland. Philos Trans R Soc Lond B Biol Sci 264: 191–294. |
| 1. Weiss D, Shotyk W, Boyle EA, Kramers JD, Appleby PG, et al. (2002) Comparative study of the temporal evolution of atmospheric lead deposition in Scotland and eastern Canada using blanket peat bogs. Sci Total Environ 292: 7–18. |
| 1. Stewart DA, Walker A, Dickson JH (1984) Pollen diagrams from Dubh Lochan, near Loch Lomond. New Phytol 98: 531–549. |
| 1. Dickson JH, Stewart DA, Baxter MS, Drndarsky ND, Thompson R, et al. (1978) Palynology, palaeomagnetism and radiometric dating of Flandrian marine and freshwater sediments of Loch Lomond. Nature 274: 548–553. |
| 1. Birks HH (1972) Studies in the Vegetational History of Scotland. III. A Radiocarbon-Dated Pollen Diagram from Loch Maree, Ross and Cromarty. New Phytol 71: 731–754. |
| 1. Edwards KJ, Whittington G, Robinson M, Richter D (2005) Palaeoenvironments, the archaeological record and cereal pollen detection at Clickimin, Shetland, Scotland. J Archaeol Sci 32: 1741–1758. |
| 1. Birks HH (1984) Late–Quaternary pollen and plant macrofossil stratigraphy at Lochan an Druim, north–west Scotland. In: Haworth E, Lund JWG, editors. Lake sediments and environmental history. Leicester: Leicester University Press. pp. 377–405. |
| 1. Edwards KJ, Whittington G (1997) A 12 000-year record of environmental change in the Lomond Hills, Fife, Scotland: Vegetational and climatic variability. Veg Hist Archaeobot 6: 133–152. |
| 1. Hall VA (1990) Recent landscape history from a County Down lake deposit. New Phytol 115: 377–383. |
| 1. Hall VA (1993) Landscape development in northeast Ireland over the last half millennium. Rev Palaeobot Palynol 82: 75–82. |
| 1. Chambers F, Price SM (1985) Palaeoecology of *Alnus* (alder): early post-glacial rise in a valley mire, north-west Wales. New Phytol 101: 333–344. |
| 1. Huntley B (1976) The past and present vegetation of Morrone Birkwoods and Caenlochan National Nature Reserves. Thesis (Ph. D.) – Cambridge: University of Cambridge. |
| 1. Huntley B, Birks HJB (1979) The past and present vegetation of Morrone Birkwoods National Nature Reseve, Scotland. I. A preliminary phytosociological survey. J Ecol 67: 417–446. |
| 1. Turner J, Innes J, Simmons I (1993) Spatial diversity in the mid-Flandrian vegetation history of North Gill, North Yorkshire. New Phytol 123: 599–647. |
| 1. Day S (1991) Post-glacial vegetational history of the Oxford region. New Phytol 119: 445–470. |
| 1. Preece R, Day S (1994) Special Paper: Comparison of Post-Glacial Molluscan and Vegetational Successions from a Radiocarbon-Dated Tufa Sequence in Oxfordshire. J Biogeogr 21: 463–478. |
| 1. Waller MP (1987) The Flandrian vegetational history and environmental development of the Brede and Pannel valleys; East Sussex. Ph.D. Thesis, Polytechnic of North London, UK. |
| 1. Waller MP (1993) Flandrian vegetational history of south-eastern England. Pollen data from Pannel Bridge, East Sussex. New Phytol 124: 345–369. |
| 1. Whittington G, Edwards KJ, Caseldine CJ (1991b) Late and Post-Glacial pollen-analytical and environmental data from a near-coastal site in northeast Fife, Scotland. Rev Palaeobot Palynol 68: 65–85. |
| 1. Bunting M (1994) Vegetation history of Orkney, Scotland; pollen records from two small basins in west Mainland. New Phytol 128: 771–792. |
| 1. Gearey B, Charman D, Kent M (2000) Palaeoecological evidence for the prehistoric settlement of Bodmin Moor, Cornwall, southwest England. Part II: Land use changes from the Neolithic to the present. J Archaeol Sci 27: 493–510. |
| 1. Day P (1996) Devensian Late-glacial and early Flandrian environmental history of the Vale of Pickering, Yorkshire, England. J Quaternary Sci 11: 9–26. |
| 1. Cayless S, Tipping R (2002) Data on mid-Holocene climatic, vegetation and anthropogenic interactions at Stanshiel Rig, southern Scotland. Veg Hist Archaeobot 11: 201–212. |
| 1. Beckett SC (1975) The late Quaternary vegetational history of Holderness. Thesis (Ph. D.) – United Kingdom: University of Hull. |
| 1. Beckett SC (1981) Pollen diagrams from Holderness, North Humberside. J Biogeogr 8: 177–198. |
| 1. Bartley DD, Chambers C, Hart-Jones B (1976) The vegetational history of parts of south and east Durham. New Phytol 77: 437–468. |
| 1. Edwards KJ, Whittington G (2010) Lateglacial palaeoenvironmental investigations at Wester Cartmore Farm, Fife and their significance for patterns of vegetation and climate change in east-central Scotland. Rev Palaeobot Palynol 159: 14–34. |
| 1. Brown A (1988) The palaeoecology of *Alnus* (alder) and the Postglacial history of floodplain vegetation. Pollen percentage and influx data from the West Midlands, United Kingdom. New Phytol 110: 425–436. |
| 1. Bottema S (1980) Palynological investigations on Crete. Rev Palaeobot Palynol 31: 193–217. |
| 1. Bottema S, Sarpaki A (2003) Environmental change in Crete: a 9000-year record of Holocene vegetation history and the effect of the Santorini eruption. Holocene 13: 733–749 . |
| 1. Bottema S (1974) Late Quaternary vegetation history of northwestern Greece. Thesis (Ph. D.) – Groningen: University of Groningen. |
| 1. Lawson I, Frogley M, Bryant C, Preece R, Tzedakis P (2004) The Lateglacial and Holocene environmental history of the Ioannina basin, north-west Greece. Quat Sci Rev 23: 1599–1625. |
| 1. Bottema S (1982) Palynological investigations in Greece with special reference to pollen as indicator of human activity. Palaeohistoria 24: 257–289. |
| 1. Turner J, Greig JRA (1975) Some Holocene pollen diagrams from Greece. Rev Palaeobot Palynol 20: 171–204. |
| 1. Lazarova M, Taylor S (2009) Boundaryless careers, social capital, and knowledge management: Implications for organizational performance. J Organ Behav 30: 119–139. |
| 1. Gerasimidis A, Athanasiadis N (1995) Woodland history of northern Greece from the mid Holocene to recent time based on evidence from peat pollen profiles. Veg Hist Archaeobot 4: 109–116. |
| 1. Kouli K, Dermitzakis MD (2010) Contributions to the European Pollen Database. 11. Lake Orestiás (Kastoria, northern Greece). Grana 49: 154–156. |
| 1. Jahns S (2005a) The Holocene history of vegetation and settlement at the coastal site of Lake Voulkaria in Acarnania, western Greece. Veg Hist Archaeobot 14: 55–66. |
| 1. Bottema S (1979) Pollen analytical investigations in Thessaly (Greece). Palaeohistoria 21: 19–40. |
| 1. Lawson I, Al-Omari S, Tzedakis P, Bryant C, Christaniss K (2005) Lateglacial and Holocene vegetation history at Nisi Fen and the Boras mountains, northern Greece. Holocene 15: 873–887. |
| 1. Grüger E (1996) Vegetation change. In: Chapman J, Shiel R, Batovic S, editors. The Changing Face of Dalmatia: Archaeological and Ecological Studies in a Mediterranean Landscape. Leicester: Leicester University Press. pp. 33–44. |
| 1. Schmidt R, Muller J, Drescher-Schneider R, Krisai R, Szeroczynska K, et al. (2000) Changes in lake level and trophy at Lake Vrana, a large karstic lake on the Island of Cres (Croatia), with respect to palaeoclimate and anthropogenic impacts during the last approx. 16,000 years. J Limnol 59: 113–130. |
| 1. Jahns S, van den Bogaard C (1998) New palynological and tephrostratigraphical investigations of two salt lagoons on the island of Mljet, south Dalmatia, Croatia. Veg Hist Archaeobot 7: 219–234. |
| 1. Juhász I (2002) Reconstitution palynologique de la végétation depuis le Tardiglaciaire dans la région de Zala. Thesis (Ph. D.) – Marseille: Aix-Marseille Université. |
| 1. Zatykó CS, Juhász I, Sümegi P (2007) Environmental archaeology in Transdanubia. Budapest: Archaeological Institute of the Hungarian Academy of Sciences. Varia Archaeologica Hungarica 20: 1–391. |
| 1. Szanto Z, Medzihradszky Z (2004) Holocene environmental changes in Western Hungary. Radiocarbon 46: 691–699. |
| 1. Juhász I, Drescher-Schneider RE, Andrieu V, de Beaulieu JL (2001) Anthropogenic indicators in a palynological record from Pölöske, Zala Region, Western Hungary. Universitätsforschungen zur Prähistorischen Archäologie Bonn 78: 29–38. |
| 1. Nagy-Bodor E, Cserny T, Nagy E (1995) Unpublished report on the study performed for the European Pollen Database. |
| 1. Wegmüller S, Lotter AF (1990) Palynostratigraphische Untersuchungen zur spät- und postglazialen Vegetationsgeschichte der nordwestlichen Kalkvoralpen. Bot Helv 100: 37–73. |
| 1. Lotter AF, Fischer J (1991) Vegetation und Flora im Gebiet des Aegelsees (Berner Oberland) im Wandel der Zeit: Vergangenheit, Gegenwart und Zukunft. Mitt Naturforsch Ges Bern 47: 77–103. |
| 1. Lotter AF, Eicher U, Birks HJB, Siegenthaler U (1992) Late-glacial climatic oscillations as recorded in Swiss lake sediments. J Quaternary Sci 7: 187–204. |
| 1. Lotter AF (1985) Amsoldingersee-Late glacial and Holocene environments of a lake at the southern edge of the Swiss plateau. Diss Bot 87: 185–208. |
| 1. Lotter AF, Heiri O, Hofmann W, Van Der Knaap WO, van Leeuwen JFN, et al. (2006) Holocene timber-line dynamics at Bachalpsee, a lake at 2265 m asl in the northern Swiss Alps. Veg Hist Archaeobot 15: 295–307. |
| 1. van der Knaap WO, van Leeuwen JFN (2001). Vegetationsgeschichte und menschlicher Einfluss in der Umgebung des Bibersees zwischen 2600 und 50 v. Chr. In: Gnepf Horisberger U, Hämmerle S, editors. Cham-Oberwil, Hof (Kanton Zug) – Befunde und Funde aus der Glockenbecherkultur und der Bronzezeit. Antiqua 33. Basel: Schweizerische Gesellschaft für Ur- und Frühgeschichte. pp. 181, 194–199. |
| 1. Markgraf V (1969) Moorkundliche und vegetationsgeschichtliche Untersuchungen an einem Moorsee an der Waldgrenze im Wallis. Bot Jahrb Syst Pflanzengesch Pflanzengeogr 89: 1–63. |
| 1. Wehrli M, Tinner W, Ammann B (2007) 16 000 years of vegetation and settlement history from Egelsee (Menzingen, central Switzerland). Holocene 17: 747–761. |
| 1. Roos-Barraclough F (2002) Retrospective analysis of net atmospheric mercury deposition rates and climatic change using peat cores. Thesis (Ph. D.) – Bern: University of Bern.166 p. |
| 1. Roos-Barraclough F, van der Knaap WO, van Leeuwen JFN, Shotyk W (2004) A Late-glacial and Holocene record of climate change from a Swiss peat humification profile. Holocene 14: 7–19. |
| 1. Heeb K, Welten M (1972) Moore und Vegetationsgeschichte der Schwarzenegg und des Molassevorlandes zwischen dem Aaretal unterhalb Thun und dem obern Emmental. Mitt Naturforsch Ges Bern 29: 1–54. |
| 1. Fäh J (1986) Erste Ergebnisse der Untersuchung zur Entwicklung eines Hangmoores im Oberen Toggenburg (Kanton St. Gallen). Telma 16: 23–30. |
| 1. Schneebeli M, Küttel M, Fäh J (1989) Die dreidimensionale Entwicklung eines Hanghochmoores im Toggenburg, Schweiz. Vierteljahrsschrift der Naturforschenden Gesellschaft in Zürich 134: 1–32. |
| 1. Wegmüller S, Welten M (1973) Spätglaziale Bimstufflagen des Laacher Vulkanismus im Gebiet der westlichen Schweiz und der Dauphiné (F.). Eclogae geologicae Helvetiae 60: 533–541. |
| 1. Welten M (1952) Über die spät- und postglaziale Vegetationsgeschichte des Simmentals. Veröffentlichungen des Geobotanischen Institutes Rübel in Zürich 26: 1–135. |
| 1. Finsinger W, Tinner W (2007) Pollen and plant macrofossils at Lac de Fully (2135 m asl): Holocene forest dynamics on a highland plateau in the Valais, Switzerland. Holocene 17: 1119–1127. |
| 1. Gobet E, Tinner W, Hochuli P, Leeuwen JFN, Ammann B (2003) Middle to Late Holocene vegetation history of the Upper Engadine (Swiss Alps): the role of man and fire. Veg Hist Archaeobot 12: 143–163. |
| 1. Ammann B (1985) Introduction and Palynology: vegetational history and core correlation at Lobsigensee (Swiss Plateau). In: Lobsigensee - Late-Glacial and Holocene environments of a lake on the central Swiss Plateau. Diss Bot 87: 127–170. |
| 1. Wehrli M, Mitchell EAD, van der Knaap W, Ammann B, Tinner W (2010) Effects of climatic change and bog development on Holocene tufa formation in the Lorze Valley (central Switzerland). Holocene 20: 325–336. |
| 1. Valsecchi V, Carraro G, Conedera M, Tinner W (2010) Late-Holocene vegetation and land-use dynamics in the Southern Alps (Switzerland) as a basis for nature protection and forest management. Holocene 20: 483–495. |
| 1. Mitchell EAD (1995) The postglacial developmental history of the Praz-Rodet Bog. Thesis (Ph. D.) – Neuchâtel: Université de Neuchâtel. |
| 1. Appleby PG, Shotyk W, Fankhauser A (1997) 210 Pb age dating of three peat cores in the Jura Mountains, Switzerland. Water Air Soil Pollut 100: 223–231. |
| 1. van der Knaap WO, van Leeuwen JFN, Fankhauser A, Ammann B (2000) Palynostratigraphy of the last centuries in Switzerland based on 23 lake and mire deposits: chronostratigraphic pollen markers, regional patterns, and local histories. Rev Palaeobot Palynol 108: 85–142. |
| 1. Lotter AF (1988a) Paläoökologische und paläolimnologische Studie des Rotsees bei Luzern. Pollen-, grossrest-, diatomeen- und sedimentanalytische Untersuchungen. Diss Bot 124: 1–189. |
| 1. Lotter AF (1988b) Past water-level fluctuations at lake Rotsee (Switzerland), evidenced by diatom analysis. In: Miller U, Robertsson AM, editors. Proceedings of the Nordic Diatomist Meeting, Stockholm, June 10–12, 1987. Stockholm: University of Stockholm. pp. 47–55. |
| 1. Ammann B, Lotter AF (1989) Late-Glacial radiocarbon- and palynostratigraphy on the Swiss Plateau. Boreas 18: 109–126. |
| 1. Lotter AF (1990) Die Entwicklung terrestrischer und aquatischer Ökosysteme am Rotsee (Zentralschweiz) im Verlauf der letzten 15000 Jahre. Mitteilungen der Naturforschenden Gesellschaft in Luzern 31: 81–97. |
| 1. Lotter AF (1999) Late-glacial and Holocene vegetation history and dynamics as shown by pollen and plant macrofossil analyses in annually laminated sediments from Soppensee, central Switzerland. Veg Hist Archaeobot 8: 165–184. |
| 1. Oeschger H, Welten M, Eicher U, Möll M, Riesen T, et al. (1980) 14C and other parameters during the Youger Dryas cold phase. Radiocarbon 22: 299– 310. |
| 1. Bradshaw RHW, McGee E (1988) The extent and time-course of mountain blanket peat erosion in Ireland. New Phytol 108: 219–224. |
| 1. Barnosky CW (1988) A late-glacial and post-glacial pollen record from the Dingle Peninsula, County Kerry. Proc R Ir Acad B 88: 23–37. |
| 1. Jessen K, Farrington A (1938) The bogs at Ballybetagh, near Dublin, with remarks on late-glacial conditions in Ireland. Proc R Ir Acad B 44: 205–262. |
| 1. Watts WA (1977) The late Devensian vegetation of Ireland. Philos Trans R Soc Lond B Biol Sci 280: 273–293. |
| 1. Cwynar LC, Watts WA (1989) Accelerator-mass spectrometer ages for late-glacial events at Ballybetagh, Ireland. Quaternary Res 31: 377–380. |
| 1. Craig AJ (1973) Studies on the ecological history of southeast Ireland. Using pollen influx analysis and other methods. Thesis (Ph. D.) – Dublin: Trinity College Dublin. |
| 1. Craig AJ (1978) Pollen percentage and influx analysis in southeast Ireland: a contribution to the ecological history of the Late-glacial period. J Ecol 66: 297–324. |
| 1. McKeever MH (1984) Comparative palynological studies of two lake sites in western Ireland and northwestern Spain. Thesis (Ph. D.) – Dublin: Trinity College Dublin. |
| 1. Mitchell FJG (1988) The vegetational history of the Killarney oak woods southwest Ireland: evidence from fine spatial resolution pollen analysis. J Ecol 76: 415–436. |
| 1. Smith A (1984) Newferry and the Boreal-Atlantic transition. New Phytol 98: 35–55. |
| 1. Smith AG, Goddard IC (1991) A 12500 years record of vegetational history at Sluggan Bog, County Antrim, Northern Ireland (incorporating a pollen zone scheme for the non-specalist). New Phytol 118: 167–189. |
| 1. Pini R, Ravazzi C, Donegana M (2009) Pollen stratigraphy, vegetation and climate history of the last 215 ka in the Azzano Decimo core (plain of Friuli, north-eastern Italy). Quat Sci Rev 28: 1268–1292. |
| 1. Hofstetter S, Tinner W, Valsecchi V, Carraro G, Conedera M (2006) Lateglacial and Holocene vegetation history in the Insubrian Southern Alps—New indications from a small-scale site. Veg Hist Archaeobot 15: 87–98. |
| 1. Noti R, van Leeuwen JFN, Colombaroli D, Vescovi E, Pasta S, et al. (2009) Mid-and late-Holocene vegetation and fire history at Biviere di Gela, a coastal lake in southern Sicily, Italy. Veg Hist Archaeobot 18: 371–389. |
| 1. Miola A, Favaretto S, Sostizzo I, Valentini G, Asioli A (2010) Holocene salt marsh plant communities in the North Adriatic coastal plain (Italy) as reflected by pollen, non-pollen palynomorphs and plant macrofossil analyses. Veg Hist Archaeobot 19: 513–529. |
| 1. Brugiapaglia E, de Beaulieu JL (1995) Etude de la dynamique végétale Tardiglaciaire et Holocène en Italie Centrale: le marais de Colfiorito (Ombrie). Comptes Rendu Academie Science Paris 321: 617–622. |
| 1. Seiwald A (1980) Beiträge zur Vegetationsgeschichte Tirols IV: Natzer Plateau - Villanderer Alm. Berichte des naturwissenschaftlich-medizinischen Vereins in Innsbruck 67: 31–72. |
| 1. Tinner W, Van Leeuwen JFN, Colombaroli D, Vescovi E, Van der Knaap W, et al. (2009) Holocene environmental and climatic changes at Gorgo Basso, a coastal lake in southern Sicily, Italy. Quat Sci Rev 28: 1498–1512. |
| 1. Brugiapaglia E (1996) Dynamique de la végétation tardiglaciaire et holocene dans les Alpes Italiennes nord-occidentales. Unpublished thesis, France, Marseille. |
| 1. Magri D (1999) Late Quaternary vegetation history at Lagaccione near Lago di Bolsena (central Italy). Rev Palaeobot Palynol 106: 171–208. |
| 1. Caroli I, Caldara M (2007) Vegetation history of Lago Battaglia (eastern Gargano coast, Apulia, Italy) during the middle-late Holocene. Veg Hist Archaeobot 16: 317–329. |
| 1. Vescovi E, Ammann B, Ravazzi C, Tinner W (2010) A new Late-glacial and Holocene record of vegetation and fire history from Lago del Greppo, northern Apennines, Italy. Veg Hist Archaeobot 19: 219–233. |
| 1. Gobet E, Tinner W, Hubschmid P, Jansen I, Wehrli M, et al. (2000) Influence of human impact and bedrock differences on the vegetational history of the Insubrian Southern Alps. Veg Hist Archaeobot 9: 175–187. |
| 1. Kaltenrieder P, Belis CA, Hofstetter S, Ammann B, Ravazzi C, et al. (2009) Environmental and climatic conditions at a potential Glacial refugial site of tree species near the Southern Alpine glaciers. New insights from multiproxy sedimentary studies at Lago della Costa (Euganean Hills, Northeastern Italy). Quat Sci Rev 28: 2647–2662. |
| 1. Drescher-Schneider R, de Beaulieu JL, Magny M, Walter-Simonnet AV, Bossuet G, et al. (2007) Vegetation history, climate and human impact over the last 15,000 years at Lago dell’Accesa (Tuscany, Central Italy). Veg Hist Archaeobot 16: 279–300. |
| 1. Colombaroli D, Vannière B, Magny CEM, Tinner W (2008) Fire-vegetation interactions during the Mesolithic-Neolithic transition at Lago delľ Accesa, Tuscany, Italy. The Holocene 18: 679–692. |
| 1. Wick L, Möhl A (2006) The mid-Holocene extinction of silver fir (*Abies alba*) in the Southern Alps: a consequence of forest fires? Palaeobotanical records and forest simulations. Veg Hist Archaeobot 15: 435–444. |
| 1. Valsecchi V, Finsinger W, Tinner W, Ammann B (2008) Testing the influence of climate, human impact and fire on the Holocene population expansion of Fagus sylvatica in the southern Prealps (Italy). Holocene 18: 603–614. |
| 1. Pini R, Ravazzi C, Reimer P (2010) The vegetation and climate history of the last glacial cycle in a new pollen record from Lake Fimon (southern Alpine foreland, N-Italy). Quat Sci Rev 29: 3115–3137. |
| 1. Kelly MG, Huntley B (1991) An 11000 year record of vegetation and environment from Lago di Mortignara, Latium, Italy. J Quaternary Sci 6: 209–224. |
| 1. Finsinger W, Bigler C, Krähenbühl U, Lotter AF, Ammann B (2006) Human impacts and eutrophication patterns during the past~ 200 years at Lago Grande di Avigliana (N. Italy). J Paleolimnol 36: 55–67. |
| 1. Sadori L, Narcisi B (2001) The Postglacial record of environmental history from Lago di Pergusa, Sicily. Holocene 11: 655–671. |
| 1. Magri D, Sadori L (1999) Late Pleistocene and Holocene pollen stratigraphy at Lago di Vico, central Italy. Veg Hist Archaeobot 8: 247–260. |
| 1. Watts WA (1985) A long pollen record from laghi di Monticchio, southern Italy. J Geol Soc London 142: 491–499. |
| 1. Watts W, Allen J, Huntley B (1996) Vegetation history and palaeoclimate of the last glacial period at Lago Grande di Monticchio, southern Italy. Quat Sci Rev 15: 133–153. |
| 1. Valsecchi V, Tinner W, Finsinger W, Ammann B (2006) Human impact during the Bronze Age on the vegetation at Lago Lucone (northern Italy). Veg Hist Archaeobot 15: 99–113. |
| 1. Watson CS (1996) The vegetational history of the northern Apennines, Italy: information from three new sequences and a review of regional vegetational change. J Biogeogr 23: 805–841. |
| 1. Belis CA, Finsinger W, Ammann B (2008) The late glacial-Holocene transition as inferred from ostracod and pollen records in the Lago Piccolo di Avigliana (Northern Italy). Palaeogeogr Palaeoclimatol Palaeoecol 264: 306–319. |
| 1. Lippi MM, Guido M, Menozzi BI, Bellini C, Montanari C (2007) The Massaciuccoli Holocene pollen sequence and the vegetation history of the coastal plains by the Mar Ligure (Tuscany and Liguria, Italy). Veg Hist Archaeobot 16: 267–277. |
| 1. Vescovi E, Ravazzi C, Arpenti E, Finsinger W, Pini R, et al. (2007) Interactions between climate and vegetation during the Lateglacial period as recorded by lake and mire sediment archives in Northern Italy and Southern Switzerland. Quat Sci Rev 26: 1650–1669. |
| 1. Pini R (2002) A high-resolution late-glacial–holocene pollen diagram from Pian di Gembro (Central Alps, Northern Italy). Veg Hist Archaeobot 11: 251–262. |
| 1. Lowe JJ, Watson C (1993) Lateglacial and early Holocene pollen stratigraphy of the northern Apennines, Italy: A contribution to the “North Atlantic seaboard programme” of IGCP-253, Termination of the Pleistocene. Quat Sci Rev 12: 727–738. |
| 1. Monegato G, Ravazzi C, Donegana M, Pini R, Calderoni G, et al. (2007) Evidence of a two-fold glacial advance during the last glacial maximum in the Tagliamento end moraine system (eastern Alps). Quaternary Res 68: 284–302. |
| 1. Ortu E, Peyron O, Bordon A, de Beaulieu JL, Siniscalco C, et al. (2008) Lateglacial and Holocene climate oscillations in the South-western Alps: An attempt at quantitative reconstruction. Quatern Int 190: 71–88. |
| 1. Di Rita F, Celant A, Magri D (2010) Holocene environmental instability in the wetland north of the Tiber delta (Rome, Italy): sea-lake-man interactions. J Paleolimnol **44:** 51–67. |
| 1. Giardini M (2007) Late Quaternary vegetation history at Stracciacappa (Rome, central Italy). Veg Hist Archaeobot 16: 301–316. |
| 1. Lucchi MR (2008) Vegetation dynamics during the Last Interglacial-Glacial cycle in the Arno coastal plain (Tuscany, western Italy): location of a new tree refuge. Quat Sci Rev 27: 2456–2466. |
| 1. Kabailiene M (1965) On the stratigraphy and palaeogeography of the Holocene in south-eastern Lithuania. Trudi Instituta geologiyi (Vilnius) 2: 302–335. |
| 1. Shulija KS, Lujanas VJ, Kibilda ZA, Banys JJ, Genutiene IK (1967) Stratigraphy and chronology of lacustrine and bog deposits of the Bebrukas lake hollow. Trudi Instituta Geologiyi, Vilnius 5: 231–239. |
| 1. Kabailiene M (1986) Methods of pollen and spores analysis. In: Istoriya ozior v SSSR, I. Tallinn. pp. 91–94. |
| 1. Stancikaite M, Baltrunas V, Sinkunas P, Kisieliene D, Ostrauskas T (2006) Human response to the Holocene environmental changes in the Birzulis Lake region, NW Lithuania. Quatern Int 150: 113–129. |
| 1. Stancikaite M, Sinkunas P, Seiriene V, Kisieliene D (2008) Patterns and chronology of the Lateglacial environmental development at Pamerkiai and Kasuciai, Lithuania. Quat Sci Rev 27: 127–147. |
| 1. Coûteaux M (1969) Recherches palynologiques en Gaume, au Pays d'Arlon, en Ardenne méridionale (Luxembourg belge) et au Gutland (Grand-Duché de Luxembourg). Acta Geographica Lovaniensia 8: 193. |
| 1. Gilot E (1968) Louvain natural radiocarbon measurements VI. Radiocarbon 10: 55–60. |
| 1. Heikkilä M, Fontana SL, Seppä H (2009) Rapid Lateglacial tree population dynamics and ecosystem changes in the eastern Baltic region. J Quaternary Sci 24: 802–815. |
| 1. Watts WA (1963) Late-glacial pollen zones in western Ireland. Ir Geogr 4: 367–376. |
| 1. Levkovskaya G (1987) Nature and man in the Mid-Holocene on the Lubanas Lowland. Riga: Zinatne. 93 p. |
| 1. Sergeeva L, Khomutova V, Trifonova I (1987) Paleogeographical stages in the development of Latgale lakes. In: Raukas A, Saarse L, editors. Palaeohydrology of the temperate zone III. Mires and lakes. Tallin: Valgus. pp. 154–163. |
| 1. Davydova N (1988) Diatom analyses of bottom deposits. In: Rational utilization of the natural resources in Belorussia "Ratsional'noe ispol'zovanie prirodnyh resursov v Belorusii". Leningrad. pp. 19–25. |
| 1. Sergeeva L, Trifonova I, Khomutova V (1988) Spore and pollen, lithological and pigment analyses of bottom deposits. In: Rational utilization of natural resources in Belorussia "Ratsional'noe ispol'zovanie prirodnyh resursov v Belorusii". Leningrad. pp. 14–19. |
| 1. Khomutova V (1989) Paleogeography and biostratigraphy of lake sediments from forest zone of European part of USSR by spore-pollen data. Thesis (Ph. D.) – Leningrad. 491 p. |
| 1. Tarasov PE, Harrison SP, Saarse L, Pushenko MYa, Andreev AA et al. (1994) Lake status records from the former Soviet Union and Mongolia: data base documentation. Paleoclimatology Publications Series Report No. 2. Colorado: World Data Center-A for Paleoclimatology, NOAA Paleoclimatology Program, Boulder. |
| 1. Wagner B, Lotter AF, Nowaczyk N, Reed JM, Schwalb A, et al. (2009) A 40,000-year record of environmental change from ancient Lake Ohrid (Albania and Macedonia). J Paleolimnol 41: 407–430. |
| 1. Bohncke SJP (1993) Lateglacial environmental changes in the Netherlands: spatial and temporal patterns. Quat Sci Rev 12: 707–717 . |
| 1. Bohncke SJP, Vandenberghe J, Huijzer AS (1993) Periglacial environments during the Weichselian late glacial in the Maas valley, the Netherlands. Geologie en Mijnbouw 72: 193–210 . |
| 1. Bos JAA, Huisman DJ, Kiden P, Hoek WZ, van Geel B (2005) Early Holocene environmental change in the Kreekrak area (Zeeland, SW-Netherlands): a multi-proxy analysis. Palaeogeogr Palaeoclimatol Palaeoecol 227: 259–289. |
| 1. Bohncke SJP, Wijmstra L (1988) Reconstruction of late-glacial lake-level fluctuations in the Netherlands based on palaeobotanical analyses, geochemical results and pollen density data. Boreas 17: 403–425 . |
| 1. Bohncke SJP, Wijmstra L, van der Woude J, Sohl H (1988) The lateglacial infill of three lake successions in the Netherlands: Regional vegetational history in relation to NW European vegetational developments. Boreas 17: 385–402 . |
| 1. Bohncke SJP, Vandenberghe J, Coope GR, Reiling R (1987) Geomorphology and palaeoecology of the Mark Valley (southern Netherlands): palaeoecology, palaeohydrology and climate during the Weichselian Late Glacial. Boreas 16: 69–85 . |
| 1. Vorren KD, Elverland E, Blaauw M, Ravna EK, Jensen CAH (2009) Vegetation and climate c. 12 300–9000 cal. yr BP at Andøya, NW Norway. Boreas 38: 401–420. |
| 1. Ramfjord H (1979a) On the late Weichselian and Flandrian shoreline displacement in Naeroy, Norway. Nor Geogr Tidsskr 62: 191–205. |
| 1. Ramfjord H (1979b) Vegetasjonsog klimahistorie gjennom de siste 9000 ar i Naeroy. Thesis (Ph. D.) – Trondheim: University of Trondheim. |
| 1. Mangerud J (1970) Late Weichselian vegetation and ice front oscillations in the Bergen district, Western Norway. Nor Geogr Tidsskr 24: 121–148. |
| 1. Bjune AE (2004) Holocene vegetation history and tree-line changes on a north-south transect crossing major climate gradients in southern Norway: evidence from pollen and plant macrofossils in lake sediment. Rev Palaeobot Palynol 133: 249–275. |
| 1. Bjune AE (2005) Holocene vegetation history and tree-line changes on a north-south transect crossing major climate gradients in southern Norway--evidence from pollen and plant macrofossils in lake sediments. Rev Palaeobot Palynol 133: 249–275. |
| 1. Eide W, Birks HH, Bigelow NH, Peglar SM, Birks HJB (2006) Holocene forest development along the Setesdal valley, southern Norway, reconstructed from macrofossil and pollen evidence. Veg Hist Archaeobot 15: 65–85. |
| 1. Bjune AE, Birks H, Seppä H (2004b) Holocene vegetation and climate history on a continental-oceanic transect in northern Fennoscandia based on pollen and plant macrofossils. Boreas 33: 211–223. |
| 1. Hyvärinen H (1976) Flandrian pollen deposition rates and tree-line history in northern Fennoscandia. Boreas 5: 163–175. |
| 1. Overland A, Hjelle KL (2009) From forest to open pastures and fields: cultural landscape development in western Norway inferred from two pollen records representing different spatial scales of vegetation. Veg Hist Archaeobot 18: 459–476. |
| 1. Odland A, Sivertsen S, Nordmark O, Botnen A, Brunstad B (1985) Stordalsvassdragei i Etne. Rapport 35. Bergen: Botanical Institute, University of Bergen. |
| 1. Paus A (2010) Vegetation and environment of the Rødalen alpine area, Central Norway, with emphasis on the early Holocene. Veg Hist Archaeobot 19: 29–53. |
| 1. Paus AA, Jevne OE (1987) Innerdalens historie belyst ved den pollenanalystyk methoden. Rapport arkeologisk serie 1987 1: 7–89. |
| 1. Jensen C, Vorren KD (2008) Holocene vegetation and climate dynamics of the boreal alpine ecotone of northwestern Fennoscandia. J Quaternary Sci 23: 719–743. |
| 1. Paus AA (1982) Paleo-okologiske undersokelser pà Froya. Thesis (Ph. D.) – Trondheim: University of Trondheim. |
| 1. Paus A, Velle G, Larsen J, Nesje A, Lie Ø (2006) Lateglacial nunataks in central Scandinavia: Biostratigraphical evidence for ice thickness from Lake Flåfattjønn, Tynset, Norway. Quat Sci Rev 25: 1228–1248. |
| 1. Barnett C, Dumayne-Peaty L, Matthews JA (2001) Holocene climatic change and tree-line response in Leirdalen, central Jotunheimen, south central Norway. Rev Palaeobot Palynol 117: 119–137. |
| 1. Jensen C (2004) The vegetation history of a coastal stone-age and iron-age settlement at 70 N, Norway. Veg Hist Archaeobot 13: 269–284. |
| 1. Zelikson EM (1971) Palynological investigation of a Holocene peat deposit from Spitsbergen. In: Neustadt MI, editor. Holocene palynology. For the Third International Palynologic Conference (Novosibirsk, USSR, 1971). Moscow: Academy of Sciences of the USSR. pp. 199–212. |
| 1. Vorren KD (2005) Stone Age settlements at Sørøya, sub-arctic Norway: impact on the vegetation. Veg Hist Archaeobot 14: 1–13. |
| 1. Bjune AE, Birks H (2008) Holocene vegetation dynamics and inferred climate changes at Svanåvatnet, Mo i Rana, northern Norway. Boreas 37: 146–158. |
| 1. Bjune AE, Bakke J, Nesje A, Birks HJB (2004a) Holocene mean July temperature and winter precipitation in western Norway inferred from palynological and glaciological lake-sediment proxies. Holocene 15: 177–189. |
| 1. Hyvärinen H (1985) Holocene pollen history of the Alta area, an isolated pine forest north of the general pine forest region in Fennoscandia. Ecol Mediterr 11: 69–71. |
| 1. Binka K, Madeyska T, Marciniak B, Szeroczynska K, Wieckowski K (1988) Bledowo lake (central Poland): history of vegetation and lake development during the last 12kyr. Bull Acad Pol Sci Biol 36: 147–158. |
| 1. Binka K, Ciesla A, Lacka B, Madeyska T, Marciniak B, et al. (1991) The development of Bledowo Lake (central Poland) - A palaeoecological study. Studia Geologica Polonica 100: 3–85. |
| 1. Wieckowski S, Szczepanek K (1963) Assimilatory pigments from subfossil needles (*Abies alba* Mid.). Acta Societatis Botanicorum Poloniae 32: 101–111. |
| 1. Szczepanek K (2001) Late Holocene vegetation history in the Dukla Pass region (Low Beskidy, Carpathians) based on pollen and macrofossil analyses. Acta Palaeobotanica 41: 341–353. |
| 1. Szczepanek K (2001) Anthropogenic vegetation changes in the region of the Dukla Pass, the Lower Beskid Mountains, Polska Akademia Umiejetnosci. Prace Komisji Prehistorii Karpat 2: 171–182. |
| 1. Szczepanek K (1971) Kras staszowski w swietle badan paleobotanicznych. Acta Palaeobotanica 12: 60–140. |
| 1. Latałowa M (1982a) Major aspects of the vegetational history in the Eastern Baltic coastal zone of Poland. Acta Palaeobotanica 22: 47–63. |
| 1. Latałowa M (1982b) Postglacial vegetational changes in the eastern Baltic coastal zone of Poland. Acta Palaeobotanica 22: 179–249. |
| 1. Ralska–Jasiewiczowa M, Latalowa M (1996) Poland. In: Berglund BE, Birks HJB, Ralska–Jasiewiczowa M, Wright HE, editors. Palaeoecological Events During the Last 15000 Years. Chichester, England: Wiley. |
| 1. Milecka K (1991) Pollen analysis of lake sediments in Giecz – the state of the investigation. In: Tobolski K, editor. Wstęp do paleoekologii Lednickiego Parku Krajobrazowego. Poznań: Wydawn Nauk Uniwersytetuim Adama Mickiewicza. pp. 147–150. |
| 1. Wacnik A (2009) From foraging to farming in the Great Mazurian Lake District: palynological studies on Lake Miłkowskie sediments, northeast Poland. Veg Hist Archaeobot 18: 187–203. |
| 1. Szczepanek K (1987) Late-Glacial and Holocene pollen diagrams from Jasiel in the low Beshid Mountains, the Carpathians. Acta Palaeobotanica 27: 9–26. |
| 1. Szczepanek K (1989a) Type region P-c: Low Beskidy Mountains. Acta Palaeobotanica 29: 17–23. |
| 1. Harmata K (1995) A Late Glacial and Early Holocene profile from Jaslo and recapitulation of the studies on the vegetational history of the Jaslo-Sanok Depression in the last 13000 years. Acta Palaeobotanica 35: 15–45. |
| 1. Zachowicz J, Przybylowska-Lange W, Nagler J (1982) The Late-Glacial and Holocene vegetational history of the Zulawy region, N. Poland. A biostratigraphic study of lake Druzno sediments. Acta Palaeobotanica 22: 141–161. |
| 1. Zachowicz J, Kepinska U (1987) The paleoecological development of Lake Druzno (Vistula Deltoic Area). Acta Palaeobotanica 27: 227–249. |
| 1. Tobolski K (1987) Holocene vegetational development based on the Kluki reference site in the Gardno Leba Plain. Acta Palaeobotanica 27: 179–222. |
| 1. Lauterbach S, Brauer A, Andersen N, Danielopol DL, Dulski P, et al. (2011) Multi-proxy evidence for early mid-Holocene environmental and climatic changes in northeastern Poland. Boreas 40: 57–72. |
| 1. Makohonienko M, Walanus A (1991) Analizy numeryczne wyników badań palinologicznych osadów Jeziora Lednica. In: Tobolski K, editor. Wstęp do paleoekologii Lednickiego Parku Krajobrazowego. Poznań: Wydawn Nauk Uniwersytetuim Adama Mickiewicza. pp. 71–79. |
| 1. Ralska-Jasiewiczowa M (1966) Bottom sediments of the Mikolajki Lake (Masurian Lake District) in the light of palaeobotanical investigations. Acta Palaeobotanica 7: 3–118. |
| 1. Ralska-Jasiewiczowa M (1989) Type Region P-x: Masurian Great Lakes District. Acta Palaeobotanica 29: 95–100. |
| 1. Rybak M, Rybak J, Zadrozna M (1987) Palaeolimnology of a small oligotrophic lake on Wolin Island, Baltic Sea, Poland. Hydrobiologia 146: 169–179. |
| 1. Rybak M (1987) Fossil chrysophycean cyst flora of Racze Lake, Wolin Island (Poland) in relation to palaeoenvironmental conditions. Hydrobiologia 150: 257–272. |
| 1. Latałowa M (1989c) Type region P-u: Baltic Shore, W Part Wolin Island. Acta Palaeobotanica 29: 115–120. |
| 1. Latałowa M (1992a) Forest changes at the Mesolithic/Early Neolithic transition and in the migration period on Wolin Island (northwest Poland). Paläoklimaforschung 8: 139–155. |
| 1. Latałowa M (1992b) Man and vegetation in the pollen diagrams from Wolin Island. Acta Palaeobotanica 32: 123–249. |
| 1. Latałowa M (1992c) The last 1500 years on Wolin Island in the light of palaeobotanical studies. Festschrift Prof. van Zeist. Rev Palaeobot Palynol 73: 213–226. |
| 1. Tobolski K (1990) Paläoökologische Untersuchungen des Siedlungsgebietes im Lednica Landschaftspark (Nordwestpolen). Offa 47: 109–131. |
| 1. Tobolski K (1991). Wstep Do Paleoekologii Lednickiego Parku Krajobrazowego. Poznań: Wydawn Nauk Uniwersytetuim Adama Mickiewicza. 157 p. |
| 1. Miotk-Szpiganowicz G (1992) The history of vegetation of Bory Tucholskie and the role of man in the light of palynological investigations. Acta Palaeobotanica 32: 39–122. |
| 1. Ralska-Jasiewiczowa M, Rzetkowska A (1987) Wyniki analizy pylkowej i makroskopowej kopalnych osadow jeziornych z Niechorza I na Wybrzezu Baltyckim. Acta Palaeobotanica 27: 153–178. |
| 1. Obidowicz A (1988) The Puscizna Rekowianska raised bog. In: Starkel L, Rutkowski J, Ralska–Jasiewiczowa M, editors. Lateglacial and Holocene environment changes: Vistula Basin 1988. Excursion Guide Book - Symposium. Cracow: AGH. pp. 87–90. |
| 1. Obidowicz A (1989) Type Region P-a: Inner West Carpathians- Nowy Targ Basin. Acta Palaeobotanica 29: 11–15. |
| 1. Obidowicz A (1990) Eine pollenanalytische und moorkundliche Studie zur Vegetationsgeschichte des Podhale-Gebietes (West-Karpaten). Acta Palaeobotanica 30: 147– 219. |
| 1. Obidowicz A (1993) Wahania gornej granicy lasu w poznym plejstocenie i holocenie w Tatrach. Dokumentacja Geograficzna 4–5: 31–43. |
| 1. Harmata K (1987) Late-Glacial and Holocene history of vegetation at Roztoki and Tarnowiec near Jaslo (Jaslo-Sanok Depression). Acta Palaeobotanica 27: 43–65. |
| 1. Drozdowski E (1974) Geneza Basenu Grudziadzkiego w świetle osadów i form glacjalnych. Prace Geograficzne, 104. Wroclaw: Zakład Narodowy im. Ossolońskich. 139 p. |
| 1. Szczepanek K (1961) Polanoglacjalna i holocenaska historia rosalinnoasci Gor swietokrzyskich. Acta Palaeobotanica 2: 3–45. |
| 1. Szczepanek K (1982) Development of the peat-bog at Slopiec and the vegetational history of the Swietokrzyskie (Holy Cross) Mountains in the last 10,000 years. Acta Palaeobotanica 22: 117–130. |
| 1. Szczepanek K (1989b) Type Region P-j: Swietokrzyskie Mountains (Holy Cross Mountains). Acta Palaeobotanica 29: 51–55. |
| 1. Szczepanek K (1992) The peat-bog at Slopiec and the history of the vegetation of the Gory Swietokrzyskie Mountains (Central Poland) in the past 10,000 years. Veröffentlichungen des Geobotanischen Institutes, Stiftung Rübel 107: 365–369. |
| 1. Gil E, Kotarba A, Szczepanek K (1972) The site II-3: T landslide at Szymbark-Kamionka. In: Excursion Guide Book, INQUA Holocene Symp., 1 (Poland). pp. 42–45. |
| 1. Gil E, Gilot E, Kotarba A, Starkel L, Szczepanek K (1974) An early Holocene landslide in the Niski Beskid and its significance for palaeogeographical reconstructions. Studia Geomorphologica Carpatho-Balcanica 8: 69–83. |
| 1. Latałowa M, Borówka RK (2006) The Allerød/Younger Dryas transition in Wolin Island, northwest Poland, as reflected by pollen, macrofossils, and chemical content of an organic layer separating two aeolian series. Veg Hist Archaeobot 15: 321–331. |
| 1. Latałowa M (1976) Diagram pylkowy osadow poznoglacjalnych i holocenskich z torfowiska w Wolbromiu. Acta Palaeobotanica 17: 55–80. |
| 1. Latałowa M (1989a) Type region P-h: The Silesia-Cracov Upland. Acta Palaeobotanica 29: 45–49. |
| 1. Latałowa M, Nalepka D (1987) A study of the Late-Glacial and Holocene vegetational history of the Wolbrom area (Silesian-Cracovian Upland-S. Poland). Acta Palaeobotanica 27: 75–115. |
| 1. Ciesla A, Ralska-Jasiewiczowa M, Stupnicka E (1978) Palaeobotanical and geochemical investigations of the lacustrine deposits at Woryty near Olsztyn (northeast Poland). Polskie Archiwum Hydrobiologii 25: 61–73. |
| 1. Pawlikowski M, Ralska-Jasiewiczowa M, Schönborn W, Stupnicka E, Szeroczynska K (1982) Woryty near Gietrzwald, Olsztyn Lake District, NE Poland - vegetational history and lake development during the last 12,000 years. Acta Palaeobotanica 22: 85–117. |
| 1. Noryskiewicz B, Ralska-Jasiewiczowa M (1989) Type Region P-w: Dobrzyn-Olsztyn Lake Districts. Acta Palaeobotanica 29: 85–93. |
| 1. Van der Knaap W, Van Leeuwen J (1997) Late Glacial and early Holocene vegetation succession, altitudinal vegetation zonation, and climatic change in the Serra da Estrela, Portugal. Rev Palaeobot Palynol 97: 239–286. |
| 1. Van der Knaap W, Van Leeuwen J (1995) Holocene vegetation succession and degradation as responses to climatic change and human activity in the Serra de Estrela, Portugal. Rev Palaeobot Palynol 89: 153–211. |
| 1. van der Knaap WO, van Leeuwen JFN (1984) Holocene vegetation, human impact, and climatic change in the Serra da Estrela, Portugal. Diss Bot 234: 497–536. |
| 1. Janssen CR, Wolderingh RE (1981) A preliminary radiocarbon dated pollen sequence from the Serra da estrela, Portugal. Finisterra 16: 299–309. |
| 1. van der Brink LM, Janssen CR (1985) The effect of human activities during cultural phases on the development of montane vegetation in the Serra da Estrela, Portugal. Rev Palaeobot Palynol 44: 193–215. |
| 1. Moe D, van der Knaap WO (1990) Transhumance in mountain areas: additional interpretation of three pollen diagrams from Norway, Portugal and Switzerland. Pact 31: 91–103. |
| 1. van der Schriek T, Passmore DG, Stevenson AC, Rolao J (2007) The palaeogeography of Mesolithic settlement-subsistence and shell midden formation in the Muge valley, Lower Tagus Basin, Portugal. Holocene 17: 369–387. |
| 1. Bodnariuc A, Bouchette A, Dedoubat J, Otto T, Fontugne M, et al. (2002) Holocene vegetational history of the Apuseni mountains, central Romania. Quat Sci Rev 21: 1465–1488. |
| 1. Tantau I (2003) Pollen analytic researches in the Eastern Romanian Carpathians. History of vegetation and human impact. Thesis (Ph. D.) – Marseille: Aix-Marseille III University and Babes-Bolyai of Cluj-Napoca University. |
| 1. Tantau I, Reille M, de Beaulieu JL, Farcas S (2006) Late Glacial and Holocene vegetation history in the southern part of Transylvania (Romania): pollen analysis of two sequences from Avrig. J Quaternary Sci 21: 49–61. |
| 1. Feurdean A, Wohlfarth B, Björkman L, Tantau I, Bennike O, et al. (2007b) The influence of refugial population on Lateglacial and early Holocene vegetational changes in Romania. Rev Palaeobot Palynol 145: 305–320. |
| 1. Tantau I, Reille M, de Beaulieu JL, Farcas S, Brewer S (2009) Holocene vegetation history in Romanian Subcarpathians. Quaternary Res 72: 164–173. |
| 1. Farcas S, de Beaulieu JL, Reille M, Coldea G, Diaconeasa B, et al. (1999) First 14C datings of Late Glacial and Holocene pollen sequences from Romanian Carpathes. C R Acad Sci III 322: 799–807. |
| 1. Tantau I, Reille M, de Beaulieu JL, Farcas S, Goslar T, et al. (2003) Vegetation history in the eastern Romanian Carpathians: pollen analysis of two sequences from the Mohos crater. Veg Hist Archaeobot 12: 113–125. |
| 1. Björkman L, Feurdean A, Cinthio K, Wohlfarth B, Possnert G (2002) Lateglacial and early Holocene vegetation development in the Gutaiului Mountains, northwestern Romania. Quat Sci Rev 21: 1039–1059. |
| 1. Feurdean A, Bennike O (2004) Late Quaternary palaeoecological and palaeoclimatological reconstruction in the Gutaiului Mountains, northwest Romania. J Quaternary Sci 19: 809–827. |
| 1. Feurdean A, Bennike O (2004) Late Quaternary palaeoecological and palaeoclimatological reconstruction in the Gutaiului Mountains, northwest Romania. J Quaternary Sci 19: 809–827. |
| 1. Feurdean A (2005) Holocene forest dynamics in northwestern Romania. Holocene 15: 435–446. |
| 1. Feurdean A, Klotz S, Mosbrugger V, Wohlfarth B (2008) Pollen-based quantitative reconstructions of Holocene climate variability in NW Romania, Palaeogeography, Palaeoclimatoligy. Palaeoecology 260: 494–504. |
| 1. Rösch M, Fischer E (2000) A radiocarbon dated Holocene pollen profile from the Banat mountains (Southwestern Carpathians, Romania). Flora 195: 277–286. |
| 1. Björkman L, Feurdean A, Wohlfarth B (2003) Late- Glacial and Holocene forest dynamics at Steregoiu in the Gutaiului Mountains, Northwest Romania. Rev Palaeobot Palynol 124: 79–111. |
| 1. Feurdean A, Bennike O (2008) Plant macrofossils analysis from Steregoiu NW Romania: taphonomy, representation, and comparison with pollen analysis. Studia Universitatis Babes-Bolyai. Geologia 53: 5–10. |
| 1. Feurdean A, Mosbrugger V, Onac BP, Polyak V, Veres D (2007a) Younger Dryas to mid-Holocene environmental history of the lowlands of NW Transylvania, Romania. Quaternary Res 68: 364–378. |
| 1. Kremenetski CV, Patyk-Kara NG (1997) Holocene vegetation dynamics of the southeast Kola Peninsula, Russia. Holocene 7: 473–479. |
| 1. Elina GA (1981) Principles and methods for reconstruction and mapping of Holocene vegetation. Leningrad: Nauka. 159 p. |
| 1. Elina GA, Filimonova LV (1996) Dynamics of vegetation and natural conditions in type regions Kc and Kg during the Holocene. In: Berglund BE, Birks HJB, Ralska–Jasiewiczowa M, Wright HE, editors. Palaeoecological Events During the Last 15000 Years. Chichester, England: Wiley. |
| 1. Andreev AA, Manley WF, Ingólfsson Ó, Forman SL (2001) Environmental changes on Yugorski Peninsula, Kara Sea, Russia, during the last 12,800 radiocarbon years. Glob Planet Change 31: 255–264. |
| 1. Jankovská V, Andreev AA, Panova NK (2006) Holocene environmental history on the eastern slope of the Polar Ural Mountains, Russia. Boreas 35: 650–661. |
| 1. Surova TG, Chernavskaya MM (1997) Paleobotanical description of peat bog Chistic in connection with climatic change in the Golotsen. In: Geophysical Committee: Data of meteorological studies 16. Moscow: Russian Academy of Science. pp 10–15. |
| 1. Solovieva N, Jones VJ (2002) A multiproxy record of Holocene environmental changes in the central Kola Peninsula, northwest Russia. J Quaternary Sci 17: 303–318. |
| 1. Filimonova LV, Elovicheva YaK (1988) Main stages of the development of forest and mire vegetation on the territory of the Kivach Nature Reserve. In: Bolotnye ekosistemy evropeiskogo Severa (Mire ecosystems of Northern Europe). Petrozavodsk. pp. 94–109. |
| 1. Elina GA, Filimonova LV (1987) Late–Glacial vegetation on the territory of Karelia. In: Raukas A, Saarse L, editors. Palaeohydrology of the temperate zone III. Mires and lakes. Tallinn: Valgus. pp. 53–69. |
| 1. Elina GA, Khomutova V (1987) Correlation of Holocene sequences of bottom sediments from Onega Lake and its old bays in terms of palynological data. In: Kabailene M, editor. Methods for the investigation of lake deposits: palaeoecological and palaeoclimatological aspects. Vilnius. pp. 193–203." |
| 1. Kremenetski CV, Vaschalova T, Goriachkin S, Cherkinsky A, Sulerzhitsky L (1997) Holocene pollen stratigraphy and bog development in the western part of the Kola Peninsula, Russia. Boreas 26: 91–102. |
| 1. Kremenetski KV, MacDonald GM, Gervais BR, Borisova OK, Snyder JA (2004a) Holocene vegetation history and climate change on the northern Kola Peninsula, Russia: a case study from a small tundra lake. Quatern Int 122: 57–68. |
| 1. Arslanov KA, Savelieva L, Klimanov V, Chernov S, Maksimov F, et al. (2001) New data on chronology of landscape-paleoclimatic stages in Northwestern Russia during the Late Glacial and Holocene. Radiocarbon 43: 581–594. |
| 1. Gunova VS (1975) Istoriya ozera Nero po palinologicheskim dannym. Moscow: Moscow State University. |
| 1. Aleshinskaya ZV, Gunova VS (1976) History of Nero as reflection on the surrounding landscape dynamics. In: Kalinin GP, Klige RK, editors. Problemy Paleohydrologii. Moscow: Nauka Press. pp. 214–222. |
| 1. Miettinen A, Savelieva L, Subetto DA, Dzhinoridze R, Arslanov K, et al. (2007) Palaeoenvironment of the Karelian Isthmus, the easternmost part of the Gulf of Finland, during the Litorina Sea stage of the Baltic Sea history. Boreas 36: 441–460. |
| 1. Wohlfarth B, Filimonova L, Bennike O, Bjorkman L, Brunnberg L, et al. (2002) Late-glacial and early Holocene environmental and climatic change at Lake Tambichozero, southeastern Russian Karelia. Quaternary Res 58: 261–272. |
| 1. Kremenetski CV, Vaschalova T, Sulerzhitsky L (1999b) The Holocene vegetation history of the Khibiny Mountains: implications for the post-glacial expansion of spruce and alder on the Kola Peninsula, northwestern Russia. J Quaternary Sci 14: 29–43. |
| 1. Gunova VS, Sirin AR (1995) Paleogeographical condition of bog development in Zapadnodvinskaya lowland during Holocene. In: Palynology in the Russia, Part 2. Moscow. pp. 27–36. |
| 1. Kremenetski K, Boettger T, MacDonald G, Vaschalova T, Sulerzhitsky L, et al. (2004b) Medieval climate warming and aridity as indicated by multiproxy evidence from the Kola Peninsula, Russia. Palaeogeogr Palaeoclimatol Palaeoecol 209: 113–125. |
| 1. Wohlfarth B, Tarasov P, Bennike O, Lacourse T, Subetto D, et al. (2006) Late glacial and Holocene palaeoenvironmental changes in the Rostov-Yaroslavl’area, West Central Russia. J Paleolimnol 35: 543–569. |
| 1. Gervais BR, MacDonald GM, Snyder JA, Kremenetski CV (2002) *Pinus sylvestris* treeline development and movement on the Kola Peninsula of Russia: pollen and stomate evidence. J Ecol 90: 627–638. |
| 1. Miettinen J, Grönlund E, Simola H, Huttunen P (2002) Palaeolimnology of Lake Piene-Kuuppalanlampi (Kurkijoki, Karelian Republic, Russia): isolation history, lake ecosystem development and long-term agricultural impact. J Paleolimnol 27: 29–45. |
| 1. Neustadt MI (1957) History of Usman'pine forest over the late Holocene. Voron Reserv Proc 5: 168–173. |
| 1. Surova TG, Krenke AN (1992) On the vegetation development and climate changes of Voronehz area during the Holocene. In: Geophysical Committee: Data of meteorological studies 15. Moscow: Russian Academy of Sciences. pp. 100–110. |
| 1. Paus A, Svendsen JI, Matiouchkov A (2003) Late Weichselian (Valdaian) and Holocene vegetation and environmental history of the northern Timan Ridge, European Arctic Russia. Quat Sci Rev 22: 2285–2302. |
| 1. Novenko EY, Volkova E, Nosova N, Zuganova I (2009) Late Glacial and Holocene landscape dynamics in the southern taiga zone of East European Plain according to pollen and macrofossil records from the Central Forest State Reserve (Valdai Hills, Russia). Quatern Int 207: 93–103. |
| 1. Trifonova I, Davydova N (1983) Diatoms in the plankton and sediments of two lakes of different trophic type. Hydrobiologia 103: 265–268. |
| 1. Davydova N, Subetto D, Khomutova V (1991) Paleolimnology of Vishnevskoe and Michurinskoe Lakes (Karelian Isthmus). In: Antropogennye izmeneniya sistem malyh ozer. Gidrometizdat. St. Petersburg: Gidrometizdat. pp. 195–198. |
| 1. Arslanov KhA, Davydova NN, Subetto DA, Khomutova VI (1992) Karelian Isthmus. In: Istoriya ozer Vostochno-Evropeiskoi ravniny (Lake history of East-European Plain). St. Petersburg. pp. 64–77. |
| 1. Andrič M (2004) Paleookolje v Sloveniji in severnemu delu hrvaške Istre v pozni prazgodovini. Arheološki vestnik 55: 509–525. |
| 1. Andrič M (2007) Holocene vegetation development in Bela krajina (Slovenia) and the impact of first farmers on the landscape. Holocene 17: 763–776. |
| 1. Andrič M, Massaferro J, Eicher U, Ammann B, Leuenberger MC, et al. (2009) A multi-proxy Late-glacial palaeoenvironmental record from Lake Bled, Slovenia. Hydrobiologia 631: 121–141. |
| 1. Andrič M, Kroflic B, Toman MJ, Ogrinc N, Dolenec T, et al. (2008) Late Quaternary vegetation and hydrological change at Ljubljansko barje (Slovenia). Palaeogeogr Palaeoclimatol Palaeoecol 270: 150–167. |
| 1. Rybníček K, Rybníčková E (1985) A palaecological reconstruction of precultural vegetation in the intermontane basins of the western Carpathians. Ecol Mediterr 11: 27–31. |
| 1. Jankovská V (1988a) A reconstruction of the Late-Glacial and Early-Holocene evolution of forest vegetation in the Poprad basin, Czechoslovakia. Folia Geobot 23: 303–320. |
| 1. Rybníčková E, Rybníček K (2006) Pollen and macroscopic analyses of sediments from two lakes in the High Tatra mountains, Slovakia. Veg Hist Archaeobot 15: 345–356. |
| 1. Wacnik A (1995) The vegetational history of local flora and evidences of human activities recorded in the pollen diagram from site Regetovka, northeast Slovakia. Acta Palaeobotanica 35: 253–274. |
| 1. Nilsson T (1964) Standard pollen diagramme und C14 datiengen aus dem Ageröds mosse in mittleren schonen. Lunds Universitets Årsskrift NF 59: 1–52. |
| 1. Bjelm L (1976) Deglaciation of the Smalard Highland, with special reference to deglaciation, ice thickness and chronology. Lundqua Thesis 2: 1–80. |
| 1. Björck S, Håkansson S (1982) Radiocarbon dates from Late Weichselian lake sediments in south Sweden as a basis for chronostratigraphic subdivision. Boreas 11: 141–150. |
| 1. Svensson N-O (1989) Late Weichseian and early Holocene shore displacement in the central Baltic. Thesis (Ph. D.) – Lund: Lund University. |
| 1. Hilldén A (1979) Deglaciationen i trakten av Berghemsmoränen om Göteborg. Lund: Lunds Universitet. 130 p. |
| 1. Berglund BE, Sandgren P, Barnekow L, Hannon G, Jiang H, et al. (2005) Early Holocene history of the Baltic Sea, as reflected in coastal sediments in Blekinge, southeastern Sweden. Quatern Int 130: 111–141. |
| 1. Digerfeldt G (1979) The highest shore-line on Hurneberg, southern Sweden. Geologiska Föreningens i Stockholm Förhandlinger 101: 49–64. |
| 1. Björck S, Digerfeldt G (1982a) Late Weichselian shore displacement at Hurneberg, southern Sweden, indicating complex uplift. Geologiska Föreningens i Stockholm Förhandlinger 104: 132–157. |
| 1. Björck S, Digerfeldt G (1982b) New 14C dates from Hurneberg supporting the revised deglaciation chronology of the Middle Swedish and moraine zone. Geologiska Föreningens i Stockholm Förhandlinger 103: 395–404. |
| 1. Hörnberg G, Bohlin E, Hellberg E, Bergman I, Zackrisson O, et al. (2006) Effects of Mesolithic hunter-gatherers on local vegetation in a non-uniform glacio-isostatic land uplift area, northern Sweden. Veg Hist Archaeobot 15: 13–26. |
| 1. Berglund BE, Gaillard MJ, Björkman L, Persson T (2008) Long-term changes in floristic diversity in southern Sweden: palynological richness, vegetation dynamics and land-use. Veg Hist Archaeobot 17: 573–583. |
| 1. Königsson LK (1984) Vegetationsgeschichte und Kultureinflüsse in der Landschaftsentwicklung der südschwedischen Gebirge in Härjedalen. Diss Bot 72: 177–189. |
| 1. Königsson L-K (1986) The Fjällnäs project: Natural and cultural components in landscape formation. In: Königsson L-K, editor. Nordic Late Quaternary biology and ecology. Uppsala Striae. 24: pp. 177–186. |
| 1. Björck S (1979) Late Weichselian stratigraphy of Blekinge. Thesis (Ph. D.) – Lund: Lund University. |
| 1. Björck S, Möller P (1987) Late Weichselian environmental history in southeastern Sweden during the deglaciation of the Scandinavian ice sheet. Quaternary Res 28: 1–37. |
| 1. Björck J (1987) The Allerød-Younger Dryas pollen zone boundary in an 800-year varve chronology from southeastern Sweden. GFF 121: 287–292. |
| 1. Björck J, Wastegård S (1999) Climate oscillations and tephrochronology in eastern middle Sweden during the last glacial–interglacial transition. J Quaternary Sci 14: 399–410. |
| 1. Yu SY, Berglund BE, Sandgren P, Fritz SC (2005) Holocene palaeoecology along the Blekinge coast, SE Sweden, and implications for climate and sea-level changes. Holocene 15: 278–292. |
| 1. Barnekow L (2000) Holocene regional and local vegetation history and lake-level changes in the Torneträsk area, northern Sweden. J Paleolimnol 23: 399–420. |
| 1. Digerfeldt G (1977) The Flandrian development of Lake Flarken. Regional vegetation history and palaeolimnology. University of Lund Department of Quaternary Geology. Report 13: 1–101. |
| 1. Carcaillet Ch, Hörnberg G, Zackrisson O (2012) Woody vegetation, fuel and fire track the melting of the Scandinavian ice-sheet before 9500 cal yr BP. Quaternary Res 78: 540–548. |
| 1. Yu SHIY, Andren E, Barnekow L, Berglund BE, Sandgren P (2003) Holocene palaeoecology and shoreline displacement on the Biskopsmåla Peninsula, southeastern Sweden. Boreas 32: 578–589. |
| 1. Digerfeldt G (1982) The Holocene development of Lake Sambosjon. 1. The regional vegetation history. Lundqua Report 23: 1–24. |
| 1. Wastegård S, Björck J, Risberg J (1998) Deglaciation, shore displacement and early-Holocene vegetation history in eastern middle Sweden. Holocene 8: 433–441. |
| 1. Göransson H (1991) Vegetation and man around Lake Bjärsjöholmssjön during prehistoric time. Lundqua Report 31: 1–44. |
| 1. Björck S, Digerfeldt G (1989) Lake Mullsjön - A key site for understanding the final stage of the Baltic Ice Lake east of Mt. Bellinger. Boreas 18: 209–219. |
| 1. Robertsson AM (1995) Palaeoenvironment during Preboreal-Boreal in Närke, south central Sweden. Quatern Int 27: 103–109. |
| 1. Björck S, Digerfeldt G (1991) Allerod-Younger Dryas sea level charges in southwestern Sweden and their relation to the Baltic Ice Lake development. Boreas 20: 115–133. |
| 1. Hammarlund D, Velle G, Wolfe BB, Edwards TWD, Barnekow L, et al. (2004) Palaeolimnological and sedimentary responses to Holocene forest retreat in the Scandes Mountains, west-central Sweden. Holocene 14: 862–876. |
| 1. Antonsson K, Brooks SJ, Seppä H, Telford RJ, Birks HJB (2006) Quantitative palaeotemperature records inferred from fossil pollen and chironomid assemblages from Lake Gilltjärnen, northern central Sweden. J Quaternary Sci 21: 831–841. |
| 1. Barnekow L, Bragée P, Hammarlund D, St Amour N (2008) Boreal forest dynamics in north-eastern Sweden during the last 10,000 years based on pollen analysis. Veg Hist Archaeobot 17: 687–700. |
| 1. Antonsson K, Seppä H (2007) Holocene temperatures in Bohuslän, southwest Sweden: a quantitative reconstruction from fossil pollen data. Boreas 36: 400–410. |
| 1. Bigler C, Barnekow L, Heinrichs ML, Hall RI (2006) Holocene environmental history of Lake Vuolep Njakajaure (Abisko National Park, northern Sweden) reconstructed using biological proxy indicators. Veg Hist Archaeobot 15: 309–320. |
| 1. Bottema S (1987) Chronology and climatic phases in the near east from 16,000 to 10,000 BP. In: Aurenche O, Evin J, Hours F, editors. Chronologies in the Near East: relative chronologies and absolute chronology 16.000–4.000 B.P. C.N.R.S. International symposium, Lyon (France) 24–28 November 1986, BAR International Series 379. Oxford. pp. 295–310. |
| 1. van Zeist W, Woldring H, Stapert D (1975) Late quaternary vegetation and climate of southwestern Turkey. Palaeohistoria 17: 53–144. |
| 1. Kremenetski CV (1995) Holocene vegetation and climate history of southwestern Ukraine. Rev Palaeobot Palynol 85: 289–301. |
| 1. Artushenko AT, Arap RYa, Bezusko LG (1982a) History of vegetation of western areas of Ukraine in Quartenary period. Kiev: Naukova dumka. 136 p. |
| 1. Artushenko AT, Arap RYa, Bezusko LG, Il'ves EO, Kajutkina TM, et al. (1982b) Razvitie prirody tyerritorii SSSR v pozdnem pleiystoytsyene i golotsenye (New data of Holocene vegetation of Ukraine). Moscow: Nauka. pp. 173–179. |
| 1. Bezusko LG, Klimanov VA, Sheliag–Sosonko Yu (1988) Climatic conditions in Ukraine in the Late Post–glacial and Holocene. In: Paleoklimaty golotsena Evropeiyskoy territorii SSSR. Moscow. pp. 125–135. |
| 1. Chernavskaya MM, Fogel GA (1991) Reconstruction of Polec'e and Carpations climate based on palinological data. Izvestiy Academy of Sciences 2: 98–105. |
| 1. Chernavskaya M (1988) Climatic changes in the historical past from stratigraphical sections of moors on the Russian plain. In: Data of meteorological studies. Academy of Sciences of the USSR, Soviet Geophysical Committee 14. pp. 76–85. |
| 1. Chernavskaya MM, Fogel GA (1989) Climatic change in the south-western part of the USSR during Golocen. Dokl Akad Nauk Soiuza Sov Sotsialisticheskikh Resp 307: 1474–1477. |
| 1. Fogel GA, Chernavskaya MM (1990) Information derived from indirect climatic indicators and methods of quantitative reconstruction. In: Brazdil R, editor. Climatic change in the historical and the instrumental periods. Brno: Masaryk university Brno. pp. 126–129. |
| 1. Bezusko LG, Kajutkina TM, Kovalukh NN (1982) YIII sjezd Ukrainskovo botanicheskogo obschestva. Kiev: Naukova dumka. 399 p. |
| 1. Bezusko LG, Kajutkina TM, Kovalukh NN, Artushenko AT (1985) Paleobotanical and radiological studies of deposits from bog Starniki (Maloe Polessie). Ukr Bot Z Ukr Bot Tovarystvo 42: 27–30. |
| 1. Bezusko LG, Klimanov VA (1987) Climate and vegetation of the plain part of the Ukrainian SSR West in the Late Post-glacial period. Ukr Bot Z Ukr Bot Tovarystvo 43: 54–58. |
| 1. Klimanov VA, Bezusko LG (1981) Climate and vegetation of small Polessie in Holocene. Ukr Bot Z Ukr Bot Tovarystvo 38: 24–26. |
| 1. Sheliag-Sosonko YR, Bezusko LG (1987) The oak- forests of Maloye Polessie in the Late Post-Glacial period. Ukr Bot Z Ukr Bot Tovarystvo 71: 4. |
| 1. Khotinsky NA, Bezusko LG, Cherkinsky AE (1994) Changes in vegetation in central and western regions of the Russian Plain. In: Velichko AA, Stakkel A, editors. Paleogeographical basis of the modern landscapes. Moscow: Nauka Press. pp. 111–118. |
| 1. Bezusko LG, Il'ves EO, Kajutkina TM (1980) New data on vegetation of small Polessie in Holocene. Ukr Bot Z Ukr Bot Tovarystvo 38: 24–26. |
| 1. Klimanov VA, Bezusko LG (1981) Climate and vegetation of small Polessie in Holocene. Ukr Bot Z Ukr Bot Tovarystvo 38: 24–26. |
| 1. Huhmann M, Kremenetski K, Hiller A, Bruckner H (2004) Late quaternary landscape evolution of the upper Dnister valley, western Ukraine. Palaeogeogr Palaeoclimatol Palaeoecol 209: 51–72. |
| 1. Bortenschlager S (1970) Waldgrenz- und Klimaschwankungen im pollenanalytischen Bild des Gurgler Rotmooses. Mitteilungen der Ostalpin-Dinarischen Gesellschaft für Vegetationskunde 11: 19–26 . |
| 1. Marinova E, Thiebault S (2008) Anthracological analysis from Kovacevo, southwest Bulgaria: woodland vegetation and its use during the earliest stages of the European Neolithic. Veg Hist Archaeobot 17: 223–231. |
| 1. Gaigalas AI, Dvaretskas VV, Banis YY, Davaynis GA, Kibilda ZA, et al. (1981) Radiocarbon age of river terraces in the southern Baltic. In: Isotopic and geochemical methods in biology, geology and archeology. Estonia: Tartu. pp. 28–32. |
| 1. Voznyachuk LN, Valchik MA (1978) Morphology, structure and history of the valley of the Neman in Pleistocene and Holocene. Minsk: Nauka i Tekhnika. 211 p. |
| 1. Willis K, van Andel TH (2004) Trees or no trees? The environments of central and eastern Europe during the Last Glaciation. Quat Sci Rev 23: 2369–2387. |
| 1. Amon L, Heisalu A, Veski S (2010) Late glacial multiproxy evidence of vegetation development and environmental change at Solova, southeastern Estonia. Est J Earth Sci 59: 151–163. |
| 1. Punning YA-MK, Ilomets MA, Koff TA, Rayamyae RA, Petersen I, et al. (1985) Complex stratigraphic-paleogeographic studies of lake and marsh sediments in the hollow Vällamägi (southeastern Estonia). Tallinn. |
| 1. García-Amorena I, Morla C, Rubiales JM, Manzaneque FG (2008) Taxonomic composition of the Holocene forests of the northern coast of Spain, as determined from their macroremains. Holocene 18: 819–829. |
| 1. Walker M, Bell M, Caseldine A, Cameron N, Hunter K, et al. (1998) Palaeoecological investigations of middle and late Flandrian buried peats on the Caldicot Levels, Severn Estuary, Wales. Proc Geol Assoc 109: 51–80. |
| 1. Hughes PDM, Mauquoy D, Barber KE, Langdon PG (2000) Mire-development pathways and palaeoclimatic records from a full Holocene peat archive at Walton Moss, Cumbria, England. Holocene 10: 465–481. |
| 1. Bush MB, Hall AR (1987) Flandrian *Alnus*: Expansion or Immigration?. J Biogeogr 14: 479–481. |
| 1. Gobet E, Tinner W, Bigler Ch, Hochuli PA, Ammann B (2005) Early-Holocene afforestation processes in the lower subalpine belt of the Central Swiss Alps as inferred from macrofossil and pollen records. Holocene 15: 672–686. |
| 1. Neustadt MI, Fedorova RV, Khotinskyi NA, Devirc AL, Dobkina EI (1965) Osechenskoe swamp (Kalinin region). Palaeogeography and chronology of Upper Pleistocene and Holocene according to radiocarbon method. Moscow: Nauka Press. pp. 81–87. |
| 1. Shulija KS, Lujanas VJ, Kibilda ZA, Banys JJ, Genutiene IK (1967) Stratigraphy and chronology of lacustrine and bog deposits of the Bebrukas lake hollow. Trudi Instituta Geologiyi, Vilnius 5: 231–239. |
| 1. Ilves E, Liiva A, Punning JM (1974) Radiocarbon dating in the Quaternary geology and archaeology in Estonia. Tallinn: Academy of Sciences of the Estonian SSR. |
| 1. Kullman L (1988) Holocene history of the forest-alpine tundra ecotone in the Scandes Mountains (central Sweden). New Phytol. 108: 101–110. |
| 1. Serebryannyi LR, Punning YA-M (1969) Results of pollen and radiohronometricheskogo isstedovaniya buried Holocene peat in Gorelovo-Koyerovo near Leningrad. In: Neustadt MI, editor. Holocene. Moscow: Nauka Press. pp 101–110. |
| 1. Kessel H, Punning JM (1976) On the age of maximal transgression of the Litorina Sea in Leningrad District. 23(3): 222–229. |
| 1. Liiva AA, Ekman IM, Rinne TS (1977) List of radiocarbon dating of the Institute of Zoology and Botany, Estonian Academy of Sciences. Post VII. Proc Estonian Acad Sci Biol Ecol 26: 325–336. |
| 1. Zarrina EP, Spiridonova FA, Arslanov HA, Kolesnikova TD, Simonova GF (1973) New section srednevaldayskih otlozheniy at village Shenskoe (Mologo Sheksninskaya-plane). In: History and Pleistocene stratigraphy klimaticheskaya, L., S. pp. 160–167. |
| 1. Oksanen PO, Kuhry P, Alekseeva RN (2003) Holocene development and permafrost history of the Usinsk mire, northeast European Russia. Géographie Physique et Quaternaire 57: 169–187. |
| 1. Kremenetski CV, Böttger T, Junge F, Tarasov A (1999a) Late-and postglacial environment of the Buzuluk area, middle Volga region, Russia. Quat Sci Rev 18: 1185–1205. |
| 1. Kullman L (1998) Non-analogous tree flora in the Scandes Mountains, Sweden, during the early Holocene - macrofossil evidence of rapid geographic spread and response to palaeoclimate. Boreas 27: 153–161. |
| 1. Greisman A (2006) Fire, forest and cultural landscape history during the last 11 000 years in Småland-a case study at Stavsåkra. The ESS Bulletin 4: 21–43. |
